# A multiscale model of the role of microenvironmental factors in cell segregation and heterogeneity in breast cancer development

**DOI:** 10.1371/journal.pcbi.1011673

**Published:** 2023-11-22

**Authors:** J. Roberto Romero-Arias, Carlos A. González-Castro, Guillermo Ramírez-Santiago

**Affiliations:** 1 Instituto de Investigaciones en Matemáticas Aplicadas y en Sistemas, Universidad Nacional Autónoma de México, Ciudad de México, Mexico; 2 Instituto de Matemáticas, Universidad Nacional Autónoma de México, Juriquilla Querétaro, Mexico; University of Southern California, UNITED STATES

## Abstract

We analyzed a quantitative multiscale model that describes the epigenetic dynamics during the growth and evolution of an avascular tumor. A gene regulatory network (GRN) formed by a set of ten genes that are believed to play an important role in breast cancer development was kinetically coupled to the microenvironmental agents: glucose, estrogens, and oxygen. The dynamics of spontaneous mutations was described by a Yule-Furry master equation whose solution represents the probability that a given cell in the tissue undergoes a certain number of mutations at a given time. We assumed that the mutation rate is modified by a spatial gradient of nutrients. The tumor mass was simulated by means of cellular automata supplemented with a set of reaction diffusion equations that described the transport of microenvironmental agents. By analyzing the epigenetic state space described by the GRN dynamics, we found three attractors that were identified with cellular epigenetic states: normal, precancer and cancer. For two-dimensional (2D) and three-dimensional (3D) tumors we calculated the spatial distribution of the following quantities: (i) number of mutations, (ii) mutation of each gene and, (iii) phenotypes. Using estrogen as the principal microenvironmental agent that regulates cell proliferation process, we obtained tumor shapes for different values of estrogen consumption and supply rates. It was found that he majority of mutations occurred in cells that were located close to the 2D tumor perimeter or close to the 3D tumor surface. Also, it was found that the occurrence of different phenotypes in the tumor are controlled by estrogen concentration levels since they can change the individual cell threshold and gene expression levels. All results were consistently observed for 2D and 3D tumors.

## Author summary

We introduce and analyze in detail a 2D and 3D quantitative multiscale model that describes the growth and epigenetic evolution of an avascular breast tumor. We obtain series of microarrays that describe the activation/inhibit levels of a group of genes that respond to the occurrence of oxygen and estrogen concentration gradients. We identify three cell phenotypes: normal, precancer and cancer, that are crucial to understand the tumor structure, the spatial distribution of mutations and the tumor heterogeneity. Finally, we found that the estrogen concentration gradients are related to the variation of gene expression levels and phenotypes occurrence. These findings strongly suggest that it is possible to develop epigenetic cancer treatment alternatives to either stop or reverse tumor evolution.

## Introduction

In the past decades the number and variety of quantitative models for cancer evolution has increased significantly. They have been aimed at addressing important aspects of cancer development such as tumor initiation and progression, tumor-structure, intra-tumor-heterogeneity as well as genetic evolution [1–6]. Nonetheless, the high complexity of cancer poses challenges and new opportunities for novel approaches and more elaborated quantitative models. Quantitative modeling has the ability to reveal unknown and/or unexpected biological as well as physical features and predictions that can be validated experimentally and clinically. Currently there is no consensus over how cancer is initiated, however, it is known that tumor growth happens in several different stages. The general accepted view is that a cell must undergo series of gene mutations before it becomes cancerous. That is, cancer development is the result of the gradual accumulation of mutations that enhance cell proliferation rate and inhibit cell death leading to tumor progression [7–9]. The detailed factors that drive these mutations are unknown; nonetheless, it is generally accepted that environment and heredity play important roles in cancer initiation. The external environmental effect upon genes can dramatically influence cell behavior and phenotype that can be inherited. Epigenetics is the study of changes in organisms due to the modification of gene expression that do not involve alterations in the DNA sequence. That is, epigenetic changes can influence gene expression without a change in genotype, the complete set of genes in an organism, and determine which proteins are transcribed. While different genotypes can give rise to different phenotypes, the microenvironment in which the cell or organism develops can also change the expressed phenotype. It has been observed that even genetically identical individuals growing in the same microenvironment can be very different [10].

Phenotypic plasticity is the ability of an organism to change in response to stimuli or inputs from the environment. Some studies suggest that cell adaptation to specific microenvironment is achieved by regulating the expression of genes that encode the enzymes and proteins needed for survival. In multicellular animals, the same principle is observed in cascades of genes that control the organism shape. Every time a cell divides, it gives rise to two cells; however, they may differ in the genes that are activated in spite of the fact that their complete genome is the same. Frequently, a “self-sustaining feedback loop” ensures that a cell maintains its identity and transmits it to its descendants.

The mechanisms that integrate signal transduction and cell metabolism are largely conserved between normal and cancer cells. Nonetheless, normal cells require a structured mechanism that involves extracellular stimulation, growth factors and downstream signaling pathways, for proliferation and conserved gene expression as well as cell physiology. Meanwhile, cancer cells can increase metabolic autonomy and frequently undergo mutations that chronically enhance these pathways, allowing them to maintain a metabolic phenotype of biosynthesis independently of normal physiologic constraints. A more complete understanding of the metabolic phenotype of cell proliferation is still to be discovered.

The most important epigenetic factors in breast cancer development are: metabolism [11–16] and estrogen production [17–21]. The former contributes to reprogramming several metabolic pathways that are essential for cancer cell survival and tumor growth while the latter affects significantly the cell proliferation process. However, there are other features that allow tumor cells to take up abundant nutrients and use them to produce ATP, generate biosynthetic precursors, and tolerate stresses associated with malignancy, for instance, redox stress and hypoxia. In addition, there is an emerging class of reprogrammed pathways that involve those allowing cancer cells to tolerate lack of nutrients by catabolizing macromolecules from inside or outside the cell, for example, autophagy, macropinocytosis, and lipid scavenging. This reprogramming may be regulated intrinsically by tumorigenic mutations in cancer cells or extrinsically by the influence of the microenvironment [16]. On the other hand, estrogens play a major role in promoting the proliferation of both, normal and neoplastic breast epithelium. However, there is no clear understanding of the mechanisms through which estrogen causes cancer [18]. The most widely acknowledged mechanism of estrogen carcinogenicity is its binding to its specific nuclear receptor alpha (ER-*α*) for exerting a potent stimulus on breast cell proliferation through its direct and/or indirect actions on the enhanced production of growth factors [18, 22, 23].

The incorporation of epigenetics and estrogen production into a quantitative model of cancer evolution allows the integration of both intrinsic (biochemical) and extrinsic (microenvironmental) signals into the genome dynamics during the development of breast cancer. This paper proposes and analyzes a quantitative model that incorporates these two processes by considering some metabolic aspects and the role of estrogen during cell proliferation. The former are believed to be the biochemical triggers of many signaling pathways during breast cancer evolution while the latter promotes proliferation of normal and neoplastic cells. To the best of our knowledge, up to now, there is no quantitative model that considers such integration in breast cancer evolution.

In the present paper we simulate the growth of the tumor mass by means of a cellular automata and consider a model that describes the epigenetic dynamics during the growth and evolution of an avascular tumor. The model also involves a set of genes that are believed to play an important role in breast cancer development and assembled them in a Gene Regulatory Network (GRN). The GRN dynamics is modeled with a set of nonlinear kinetic equations that describe the interactions of estrogen, and oxygen with the genes as well as the interactions between genes themselves. To understand the tumor diversity that drives cancer development, we analyze the role of the intrinsic and extrinsic cell plasticity. We also describe the gene mutation dynamics by using the Tau-Leaping Gillespie Algorithm. The simulations and analysis of the tumor growth and the mutation dynamics take place in two and three spatial dimensions to illustrate that the malignant cells are located on the tumor periphery. We also demonstrate that the results are fully consistent regardless of the simulated tumor spatial dimensionality.

## Materials and methods

The growth and development of an avascular tumor is analyzed by considering a cellular automaton together with a set reaction-diffusion equations [24, 25] that describe the transport of essential nutrients: glucose, oxygen and estrogens. It is assumed that tissue is made of four types of cells, namely: normal, precancer, cancer, and tumor necrotic cells that live in either a 2D square lattice or a 3D cubic lattice. With the aim at emulating the crowding of cancer cells and cell migration we considered the possibility that cancer cells could pile up in one lattice cell. According to this, normal and necrotic cells may occupy one lattice site; however, more than one precancer or cancer cell can accumulate at a given lattice site. Thus, three state variables are defined at each lattice site: $\sigma_{\text{n}}\left(\vec{r},t\right),\;\sigma_{\text{d}}\left(\vec{r},t\right)\in 0,1$, for normal and necrotic cells. For precancer or cancer cells $\sigma_{\text{c}}\left(\vec{r},t\right)\;\in \;N$. The vector $\vec{r}$ has two integer components, (*i*, *j*) in 2D or three integer components (*i*, *j*, *k*), in 3D with 0 ≤ *i*, *j*, *k* ≤ *L*. They denote the cell position coordinates in the lattice. An initial cancer cell is placed at about the middle of the lattice and a nutrient supply –horizontal capillary vessel– is located at the upper boundary. It was assumed that essential nutrients, glucose, oxygen as well as estrogen, diffuse from the capillary vessel throughout the tissue. Essencial nutrients and estrogen are critical for DNA synthesis as well as for cell proliferation [18, 22, 26–28]. Thus, it can be considered that essential nutrients play the role of catalysts during the expression of genes whereas estrogen regulate the cell cycle and different signaling pathways. Additionally, it is assumed that there is a competition between normal and cancer cells for essential nutrients. The abundance of these substances around cells yields fluctuations and asymmetries in gene propensities, which in turn play a role in the development of heterogeneity. On the other hand, the growth of tumor cells is typically limited to a region of approximately 10 cells from a blood vessel that supplies the tumor. As a result, there are glucose, oxygen, and estrogen spatial gradients [29]. Taking this fact into consideration, the transport of essential nutrients and estrogen in the tissue is described by the following set of three dimensionless reaction-diffusion equations [24, 25]:

$$\begin{array}{ccc} \frac{\partial C_{l}\left(\vec{r},t\right)}{\partial t} & = & \nabla^{2}C_{l}\left(\vec{r},t\right)-\alpha_{l}^{2}[\sigma_{n}\left(\vec{r},t\right)+\lambda_{l}\sigma_{c}\left(\vec{r},t\right)]C_{l}\left(\vec{r},t\right),\\ & & \text{with}\;\;l=1,2,3. \end{array}$$ (1)

The quantities $C_{l}\left(\vec{r},t\right)$, represent the glucose (*l* = 1), oxygen (*l* = 2) and estrogen (*l* = 3) concentration, respectively. The parameters $\alpha_{l}^{2}$ and λ_*l*_ represent the cells glucose (*l* = 1), oxygen (*l* = 2), and estrogen (*l* = 3), consumption and supply rates, respectively. The ability of the normal and cancer cells to compete for glucose, oxygen, and estrogens are represented by the product, $\alpha_{l}^{2}\times \lambda_{l}$. A transport equation for estrogen has also been included in Eq (1) since we have assumed that estrogen enhances the production of growth factors promoting cell proliferation of both normal and neoplastic breast cells. The source term in Eq (1) is considered in the boundary condition as a capilar vessel. The processes of cancer cell division and death that depend on the local concentration of essential nutrients and estrogen, are described independently in the next subsections. The details of how the numerical solutions of Eq (1) were calculated and their coupling to the other parts of the model are presented in the section of simulation details and numerical integration.

### Oxygen and death

Over the past decades, evidence has been accumulated showing that 50%-60% of advanced solid tumors develop hypoxic and/or anoxic tissue regions that are heterogeneously distributed within the tumor mass. Oxygen-sensing mechanisms have been developed in mammals to maintain cell and tissue homeostasis, as well as to adapt to the chronic low-oxygen conditions found in cancer. It involves the capture, binding, transport, and delivery of molecular oxygen. One of the crucial features of this network is its ability to sense and respond to low-oxygen concentration conditions. The poor vasculature in the early tumor development –avascular tumor– alters its metabolism [30]. As a result there are too many tumor regions that undergo hypoxic stress because they are located at relatively large distances from blood vessels [31]. Therefore, tumor cells have to adapt their metabolism to this unusual and harsh microenvironment that contains very small concentrations of glucose and oxygen [32].

Under these conditions, hypoxia inducible factors (HIFs) activate for the maintenance of cellular oxygen homeostasis and hypoxia adaptation [33, 34]. Oxygen is crucial in controlling vascularization, glucose metabolism, survival and tumour spread. This pleiotropic action is orchestrated by HIF, which is a master transcriptional factor in nutrient stress signalling [35]. In a hypoxic environment accelerated glycolysis ensures ATP levels that are compatible with the demand of the fast proliferating tumor cells. This shift in cellular metabolism from mitochondrial respiration to glycolysis is linked to tumor malignancy. Sustained tumor hypoxia also gives rise to adaptations which allow cells to survive and even thrive. As a consequence, a more malignant phenotype may develop due to the following factors: (i) HIF-1*α*-mediated mechanisms favoring tumor growth and malignant progression, (ii) HIF-1*α*-independent up-regulation and down-regulation of genes, and (iii) effects via genome changes, that produce hypoxia-induced apoptosis resistance, genomics instability which in turn lead to clonal heterogeneity, and selection of resistant and/or aggressive clonal variants [36]. On the other hand, it has been found that the rate of cell death increases when supply of glucose and nutrients are very low and cellular ATP is increasingly depleted. The most striking proof of hypoxia-induced apoptosis is the suppression of the electron transport chain on the inner membranes of the mitochondria [37].

Taking these observations into consideration one can assume that in the regions where there is a high cancer cell population density and a small supply of oxygen, the probability of cell death, *P*_d_, increases significantly. As suggested in [24] cancer cell death due to hypoxia can be modeled with a Gaussian probability distribution which argument depends on the ratio of the local oxygen concentration to the local cancer cells concentration at a point $\vec{r}$ at time *t*.

$$\begin{array}{c} P_{\text{d}}(C_{2}\left(\vec{r},t\right))=\text{exp}[-{\left(\frac{C_{2}\left(\vec{r,t}\right)}{\theta_{\text{d}}\sigma_{c}\left(\vec{r,t}\right)}\right)}^{2}]. \end{array}$$ (2)

The variance of this probability distribution can be tuned by adjusting the steepness of the curve given by the value of *θ*_d_. Thus, for low local oxygen concentration and high local cancer cell density population this argument is small and the probability of cell death is: $P_{\text{d}}(C_{2}\left(\vec{r},t\right))\approx 1-[C_{2}\left(\vec{r,t}\right)/\theta_{\text{d}}\sigma_{c}\left(\vec{r,t}\right){]}^{2}$, close to one. On the contrary, for high local oxygen concentration and small local cancer cell density population this argument is much greater than one, and the the probability of cell death becomes negligible. Note that when a cancer cell dies, $\sigma_{c}\left(\vec{r},t\right)\to \sigma_{c}\left(\vec{r},t\right)-1$ and $\sigma_{d}\left(\vec{r},t\right)=1$ as an indication that a cancer cell has become a necrotic cell. By including the local oxygen concentration $C_{2}\left(\vec{r},t\right)$ at a given time in the argument of Eq (2) we establish an intrinsic coupling with the reaction-diffusion Eq (1), which solution describes the oxygen concentration at that location.

These assumptions may also explore the fact that exposure to cycling hypoxia leads to high levels of reactive oxygen species (ROS) from cell mitochondria. This is because several oxygen-dependent enzymes function as primary cellular oxygen sensors, and change in their activity during hypoxia, influencing important adaptive signaling pathways. In particular, several mitochondrial enzymes are capable of ROS production [38]. On the other hand, it has been demonstrated that reoxygenation induces significant amounts of DNA damage and malignant progression because of the presence of ROS during reoxygenation [39, 40]. Thus, ROS influences the tumor microenvironment and is known to initiate cancer angiogenesis, metastasis, and survival at different concentrations [41]. However, the detailed molecular mechanisms of how cancer cells respond to oxidative stress are still to be determined.

### Estrogen and cell cycle

Estrogen gives rise to diverse biological effects as a result of its direct interaction with an intracellular receptor that activates the expression of genes encoding proteins with important biological functions [42–45]. In terms of the molecular mechanism estrogen induces gene expression and synthesis of specific proteins, activation of specific enzymes, and proliferation in certain cell types. All of these actions appear to require the binding of the hormone to a specific receptor protein [46]. In particular, one of the most notable estrogen effects is its potential mitogenic action in hormone sensitive breast epithelial tissues [47, 48]. Because of this, the establishment of estrogen concentration gradients is crucial for cell proliferation and cancer progression. By contrast, nutrient availability facilitates nutrient cell consumption which favors mutation rates, whereas limited or lack of nutrient leads to latent cell states with small mutation rates. This latter hypothesis was modeled in [25], as a stochastic process which was coupled to the nutrients transport reaction-diffusion equations. The dynamics described with this coupling yielded genetic spatial heterogeneity as a result of mutations accumulation during tumor growth, a hallmark of most cancers.

Clinical and animal studies suggest that risk factors associated with breast cancer reflect cumulative exposure of the breast epithelium to estrogen [49]. During the cell cycle estrogens regulate the expression and function of cyclins, c-Myc, cyclin D1 and cyclin E-Cdk2 which are considered important in the control of G1/S phase progression [50, 51]. Furthermore, Cyclin E shows a periodic expression pattern, being synthesized during the G1-phase of the cell cycle, with sharp increasing levels during the late G1 phase, followed by the accumulation of cyclin E protein and then down regulated in the S-phase. Other CDK complexes, such as cyclins A and B, undergo opposite periodic patterns to those of cyclin E [51]. Taking all the previous statements into consideration one can surmise that the period of the cell division cycle is mainly regulated by the local concentration of estrogen $C_{3}\left(\vec{r},t\right)$.

This antagonistic process leads to out–of phase non-linear oscillations with maxima related to the transitions of the cell cycle G phases [52]. Bearing this in mind, we assumed that cyclins E and B are the two key players responsible for the out of phase oscillations. To describe these periodic oscillation patterns of the relative concentrations of cyclins B and E that drive the cell cycle, we have chosen the following relatively simple non-dimensional Lotka-Volterra system of equations.

$$\begin{array}{c} \begin{array}{ccc} \frac{\partial u(\vec{r},t)}{\partial t} & = & u\left(\vec{r},t\right)[1-v(\vec{r},t)],\\ \frac{\partial v(\vec{r},t)}{\partial t} & = & \epsilon \left(\vec{r}\right)v\left(\vec{r},t\right)[u(\vec{r},t)-1], \end{array} \end{array}$$ (3)

where $u(\vec{r},t)$ and $v(\vec{r},t)$ represent the concentrations of cyclins E and B, of a cell located at point $\vec{r}$ at time *t*, respectively. The wave shape solution depends only on the local parameter $\epsilon (\vec{r})$ and on the boundary conditions for each cell. This system of equations describes out of phase oscillations with period, $T\left(\vec{r}\right)=2\pi /\sqrt{\epsilon (\vec{r})}$.

Experimental data has shown that the cell cycle is arrested if the estrogen concentration is below or above certain threshold values [53]. This allows one to assume that the growth rate depends on the estrogen consumption rate. This quantity is directly related to the reaction term in the reaction-diffusion equation for estrogen, third equation in Eq (1). For that reason the time scale of the oscillations can be related to the estrogens local concentration through the local parameter $\epsilon (\vec{r})$ which can be expressed as

$$\begin{array}{c} \epsilon \left(\vec{r}\right)\approx (\alpha_{3}^{2}\left[\sigma_{n}\left(\vec{r}\right)+\lambda_{3}\sigma_{c}\left(\vec{r}\right)\right]C_{3}\left(\vec{r}\right){)}^{2}. \end{array}$$ (4)

This equation makes a distinction of the cell cycle duration of a normal and a cancer cell. Note that when $\sigma_{n}\left(\vec{r}\right)=1$, then, $\sigma_{c}\left(\vec{r}\right)=0$, and vice versa. In principle, one can assume that normal and cancer cells follow a similar synchrony during the cell cycle. Notwithstanding, the cell proliferation rate depends on cell estrogen consumption rate. Therefore, as estrogen consumption rate increases, the cancer cells growth factor concentration increases as well.

### Cell lineage and microenvironment

A single cell cannot influence significantly its immediate surroundings. Nonetheless, a relatively large number of cells can change significantly their microenvironment through the expression of cooperative phenotypes [54]. Tumors are made of multiple subpopulations of cells with different phenotypes, some of them are able to self renew, seed, maintain tumors, and provide a reservoir of resistant cells [55]. In this way many cell phenotypes contribute to the growth and division processes of nearby cells by changing their local environment [53].

In cancer, there are two major phases of phenotypic plasticity: initiating and maintaining plasticity. Initiating plasticity refers to the influence of the cell of origin and the specific driver mutations that occur during tumor formation. Maintaining plasticity is the result of genetic evolution and hierarchical and plastic interconversion between cellular phenotypes. These two forces collaborate to generate the tumor phenotypes that are diverse even within the same tissue [56]. Furthermore, cellular mechanisms that govern lineage proliferation and survival during development might also underlie tumorigenic mechanisms. Somatic genetic alterations show lineage-restricted patterns across human tumors, which indicate that genetic changes in cancer might be conditioned by the lineage programs embedded in the tumor precursor cells [57]. The interaction among genetically related individuals increases the propensity for cooperative phenotypes to evolve. In addition, different cell lineages segregate in space with the aim of cooperating and benefiting each other. Because of this, local populations of cancer cells often times aggregate in groups of progenitors that proliferate leading to large clusters with similar lineages [58].

Here, we apply the concept of lineage cell segregation that has been applied to analyze the evolution of bacterial colonies [54], as a measure of cell heterogeneity during tumor development. Considering that estrogen induces gene expression, synthesis of specific proteins, and activation of specific enzymes which require the binding of the hormone to a specific receptor protein, one can model the lineage production rate with a metabolic Michaels-Menten kinetics. In doing so, the segregation index, $\hat{\beta }$, at position ${\vec{r}}_{i}$ can be expressed as,

$$\begin{array}{ccc} \hat{\beta }\left({\vec{r}}_{i},t\right) & = & \frac{1}{m}\sum_{j=1}^{m}g(\vec{a}\left({\vec{r}}_{j},t\right))\times \frac{C_{3}\left({\vec{r}}_{j},t\right)}{C_{3}\left({\vec{r}}_{j},t\right)+\kappa_{\epsilon }},\\ & & \text{with}\;\;|{\vec{r}}_{i}-{\vec{r}}_{j}|\leq R_{o}, \end{array}$$ (5)

where $g(\vec{a}\left({\vec{r}}_{j},t\right))$ is the genetic activity defined by

$$\begin{array}{c} g(\vec{a}\left({\vec{r}}_{j},t\right))=\{\begin{array}{c} 0,\;\text{if}\;\vec{a}\left({\vec{r}}_{j},t\right)=\vec{a}\left({\vec{r}}_{i},t\right)\\ 1,\;\text{if}\;\vec{a}\left({\vec{r}}_{j},t\right)\neq \vec{a}\left({\vec{r}}_{i},t\right). \end{array} \end{array}$$ (6)

and $C_{3}\left({\vec{r}}_{j},t\right)$ is the local estrogen concentration, *κ*_*ϵ*_ is the saturation constant or the estrogen carrying capacity at the neighboring cells with positions ${\vec{r}}_{j}$ with *j* = 1, 2, 3, …‥, *m*, and *m* is the total number of neighboring cells within a given distance *R*_*o*_ from ${\vec{r}}_{i}$. Note that the genetic activity discourages the interaction between cells of the same genotype because they do not lead to genetic diversity. The vector $\vec{a}\left({\vec{r}}_{j},t\right)$ represents the genotype of the cell located at position ${\vec{r}}_{j}$ at time *t*, it is formed by the set of breast cancer genes that are believed to play a major role in cancer development. The gene set that defines the entries of vector $\vec{a}\left({\vec{r}}_{j},t\right)$ will be discussed in detail in the Gene Regulatory Network subsection. The form of the segregation index directly measures the spatial assortment of genetic cell lineages and ranges from 0 to 1, where 1 denotes complete lineage segregation within the spatial scale *R*_*o*_.

### Random mutations

The instability of the genome of cancer cells leads to a state that increases the spontaneous mutation rate that gives rise to a mutations cascade some of which enable cancer cells to bypass the regulatory processes that control cell location, division, expression, adaptation and death [59]. Mutations either arise from copying a repaired DNA damage or from errors that happen during DNA synthesis [60, 61]. To describe quantitatively the cascade of spontaneous mutations, we assumed a birth-and-death process that considers that cell population consists of cells within a genus where the creation of a new cell is due to mutations without considering the probability of dying out and the size of the species [62]. These processes are characterized by the property that whenever a transition occurs from one state to another, the transition happens to a neighboring state only. Thus, we modeled the mutation dynamics by means of a Yule-Furry Markovian process which is described by the following master equation [63].

$$\begin{array}{ccc} \frac{dP_{x}\left(\vec{r},t\right)}{dt} & = & -\gamma \left(\vec{r},t\right)xP_{x}\left(\vec{r},t\right)+\gamma \left(\vec{r},t\right)\left(x-1\right)P_{x-1}\left(\vec{r},t\right),\\ & & \text{with}\;\;x\geq 1. \end{array}$$ (7)

Here $P_{x}\left(\vec{r},t\right)$ is a geometric probability distribution with argument, $p(\vec{r},t)$ [63] and represents the probability that a given cell in the tissue at position $\vec{r}$ undergoes, *x* (*x* = 0, 1, 2, …) mutations at a given time *t* with a hopping probability $\gamma (\vec{r},t)>0$, that one new mutation, *x* = *x* + 1, will happen in the time interval [*t*, *t* + *dt*). It has been found that cancer cells remodel tissue microenvironment and specialized niches to their competitive advantage [64, 65]. Here we assume that the essential nutrients spatial gradients change the tissue microenvironment and cell niches somehow modifying the acquisition rate of new spontaneous mutations [66, 67]. For simplicity one can surmise a direct relationship between the glucose concentration and the hopping probability of acquiring new mutations, namely, $p\left(\vec{r},t\right)=\text{exp}\left(\int_{0}^{t}\gamma \left(\vec{r},\tau \right)d\tau \right)$. Therefore, one can propose the following *ansatz*, $p\left(\vec{r},t\right)=\text{exp}\left(\int_{0}^{t}\gamma \left(\vec{r},\tau \right)d\tau \right)=\text{exp}[-{\left(\frac{C_{1}\left(\vec{r},t\right)}{\theta_{\text{div}}}\right)}^{2}]$, where $C_{1}\left(\vec{r},t\right)$ represents the glucose concentration at the cell position $\vec{r}$ at time *t*, and $\theta_{\text{div}}$ is an adjustable parameter that controls the shape of the sigmoidal curve. Thus, there is an intrinsic nonlinear coupling between the master Eq (7) that describes the mutation dynamics of a given cell located at position $\vec{r}$ at time *t* with the first of the reaction-diffusion Eq (1) that describes the glucose concentration at that location.

On the other hand, spontaneous mutations can be randomly activated by one of the following mechanisms: structural alterations resulting from mutation or gene fusion, by juxtaposition of enhancer elements, or by amplification acquisition of random mutations. This random activation can be modeled with a Poisson probability distribution [26, 68–70]. Thus, the total probability distribution that a cell in the tissue located at $\vec{r}$ at time *t*, undergoes a mutation is written as the product:

$$\begin{array}{c} P_{rm}\left(\vec{r},t\right)=G(p\left(\vec{r},t\right),z_{j})N_{\lambda }\left(\lambda ,k_{j}\right). \end{array}$$ (8)

Here, $G(p\left(\vec{r},t\right),z_{j})$ is a geometric probability distribution with mean $(1-p(\vec{r},t))/p\left(\vec{r},t\right)$, where $p(\vec{r},t)$ is given by the *ansatz* indicated above, and *z*_*j*_ is the number of viable mutations of gene *j*. The Poisson probability distribution *N*_λ_(λ, *k*_*j*_) with mean λ represents the probability of occurrence of *k*_*j*_ mutations of gene *j* at a given cell. This factorization has proven to be very useful in the implementation of the stochastic simulations that describe tumor gene dynamics [25]. In the forthcoming subsection a brief description of the stochastic mutation dynamics is presented.

### Stochastic dynamics

To simulate the stochastic mutation dynamics we used the Tau-Leaping Gillespie algorithm [71] which has demonstrated its usefulness in the simulations of different processes in molecular biology. Let us assume that at a given time *t*, the state of the system formed by *N* genes is defined by the vector **x**(*t*) = (*x*_1_(*t*), …, *x*_*N*_(*t*)), in which each coordinate represents the number of mutations in each gene. Then, the change of this state vector in the time interval [*t*, *t* + *τ*) is given as

$$\begin{array}{c} \text{x}\left(t+\tau \right)\to \text{x}\left(t\right)+\sum_{j}{\text{k}}_{j}\nu_{j}, \end{array}$$ (9)

where **k**_*j*_ is a vector of random numbers generated from a Poisson distribution with mean **a**_*j*_ × *τ* and *ν*_*j*_ is the vector that changes the mutations of gene *j*, by 0, or 1. The selection of gene *j* is made by choosing a random number from a set of numbers distributed according to the negative binomial distribution. Let **a**_**j**_(**x**) be the propensity functions that represent the probability of having one mutation of gene *j* at time *t*. Since mutations occur with equal probability regardless of the chosen gene the values of **a**_**j**_(**x**) can be set equal to one for every gene. Therefore, the time *τ* required for the number of mutations to increase by one unit is:

$$\begin{array}{c} \tau =\frac{1}{{\text{a}}_{0}\left(\text{x}\right)}ln\left(\frac{1}{r_{j}}\right), \end{array}$$ (10)

where ${\text{a}}_{0}\left(\text{x}\right)=\sum_{j=1}^{\text{n}}{\text{a}}_{\text{j}}\left(\text{x}\right)$ and *r*_*j*_ is a random number uniformly distributed in the interval [0, 1] corresponding to gene *j*. Since tumor evolution dynamics is the result of the coupling of gene mutations, essential nutrient and estrogen dynamics, an extended version of the tau-leaping method was applied to obtain an effective sampling of the biophysical relevant quantities [72–75]. Thus, the change of the system’s state **x**(**t**) during a time *τ* occurs in accordance with the following equation

$$\begin{array}{c} \text{x}\left(t+\tau \right)\to \text{x}\left(\text{t}\right)+\sum_{\text{j}}\sum_{\text{i}}{\text{k}}_{\text{i}}{\text{z}}_{\text{i}}\nu_{\text{j}}, \end{array}$$ (11)

where **z**_**i**_ is a column vector formed with random numbers obtained from a geometric distribution, and **k**_**i**_ is a row vector with random numbers generated from a Poisson distribution. We chose these random numbers distributions because mutations dynamics is described by the product of these two probability distributions. See Eq (8).

### Mutations induced by microenvironmental factors

There is a growing evidence suggesting that tumor microenvironment per se constitutes a significant source of genetic instability. The induction of mutagenesis and numerous types of DNA damage, including DNA strand breaks and oxidative base damage are associated with the adverse and harsh conditions of the microenvironment [66, 76]. In this way, many solid tumors, including breast cancer, are composed of heterogeneous cell lineages that interact with the microenvironment through complex networks [77]. In the following paragraphs we consider the influence of essential nutrients and estrogen on mutations. According to the above proposed *ansatz*, the probability $P_{x}\left(\vec{r},t\right)=p\left(\vec{r},t\right)$, in the master Eq (7), represents the probability of occurrence of genetic mutations as a result of glucose concentration gradients. On the other hand, most of the estrogen actions on the normal and neoplastic mammary cells are mediated via estrogen receptors, mainly for controlling cell proliferation. The probability of mutations *P*(*B*) can be increased by a failure of estrogen receptors as a consequence of genotoxic estrogen metabolites driven by estrogen concentration gradients [78]. By assuming that $P_{x}\left(\vec{r},t\right)=P\left(A\right)$, we obtain the probability that glucose and estrogen concentration gradients influence mutations simultaneously but independently, that is, *P*(*A* ∪ *B*) = *P*(*A*) + *P*(*B*) − *P*(*A*) × *P*(*B*). Unfortunately, the mathematical expression for probability of mutations *P*(*B*) is unknown and it is very difficult to estimate. In spite of this limitation, here we explore its role by considering it as a varying parameter. To elaborate more on the probability *P*(*A* ∪ *B*) let us recall that tumor cells interact with diverse cellular lineages and regulate the hierarchy of tumorigenic cells [79]. Because of this, the local segregation index, $\hat{\beta }\left({\vec{r}}_{i},t\right)$, can be thought of as a probabilistic measure of the occurrence of mutations due to genetic instability induced by metabolic and micro-environmental conditions. In addition, estrogen controls cell proliferation so that it can be considered as the main source of cell lineage. Then the total probability of mutations can be written as the product: $\hat{\beta }\left(\vec{r},t\right)\times P\left(B\right)$. Therefore, the probability that segregation as well as glucose and estrogen concentration gradients lead to mutations can be written as:

$$\begin{array}{c} P\left(A\cup B\right)\equiv P_{rm}\left(\vec{r},t\right)=p\left(\vec{r},t\right)+\hat{\beta }\left(\vec{r},t\right)(1-p(\vec{r},t))\times P\left(B\right). \end{array}$$ (12)

Notice that when estrogen spatial gradients become negligible, *P*(*B*) = 0, then mutations are directly related to glucose spatial gradients as has been analyzed in a previous model [25].

### Gene Regulatory Network

Gene Regulatory Networks (GRN) are effective and useful models that are commonly used to study the complex regulatory mechanisms of a cell. A GRN is the collection of molecular species and their interactions which control gene-product abundance [80]. GRN’s encode the patterns of interacting signals responsible for the up-and downregulation of genes. GRN’s integrate internal and external signals to ensure that a cell develops an appropriate response for its current environment [81]. Due to the network interactions, each gene changes its expression level and the GRN dynamics changes as a whole leading to different expression patterns [82]. As GRN dynamics evolves, it eventually settles down into an equilibrium state that complies with all the regulatory interactions [83]. The most notable topological features of a GRN state space are its basins of attraction. A basin of attraction is the set of states that moves over time towards a region called an attractor. Attractor states represent stable equilibrium states analogous to the *lowest energy states* at the bottom of a potential well. Attractors encode specific *genetic programs* of the cell that are pre-programmed in the GRN, including those which produce a stable cell type-specific gene expression patterns that lead to cell phenotypes. The basins of attraction are separated by hills that represent unstable states. Transitions between two attractors represent a switch between two cell phenotypes. They are triggered by regulatory signals that change the expression status of a set of genes in a concerted manner or by gene expression noise which produces random fluctuations in the expression of the genes [83]. The detailed state space topography guides the production of distinct cell phenotypes at the right place and time, leading to the epigenetic landscape. Therefore, understanding the dynamics of GRN’s is crucial to comprehend the development and the occurrence of diseases such as cancer.

The interplay between regulatory networks and metabolism and how an organism adapts to its microenvironment can also be described by GRNs [80, 84]. In the epigenetic landscape not all attractors represent physiological cell phenotypes. These unphysiological attractors are byproducts of the complex dynamics of the GRN, and the majority of them are associated to abnormal, non-viable gene expression patterns that may be the result of conflicting signals. From this point of view, if an unphysiological attractor is associated with a viable proliferative phenotype it could represent cancer. Thus, cancer can be viewed as a set of abnormal gene expression patterns that are related to unphysiological attractors in the dynamics of a GRN. A first general description of this simple but profound idea was proposed fifty years ago by Kauffman [85].

*Kinetic model*– In this paper, we consider a kinetic model of molecular regulatory interactions between a set of ten genes that are believed to play a relevant role in breast cancer development since they control cell cycle progression, DNA repair, metabolism reprogramming and control growth factors and apoptosis signaling. The set of genes TP53, ATM, HER2, BRCA1, AKT1, ATR, CHEK1, MDM2, CDK2 and P21 with transcriptional positive and negative regulations can be assembled as a GRN at the level of a single cell [86]. Tumor suppressor gene TP53 controls the cellular genome’s integrity and is an important regulator of cell cycling, proliferation, apoptosis and metabolism. The loss of control over the cell cycle, results in the acceleration of cell proliferation and facilitates metabolic reprogramming, giving the pre-malignant cells numerous advantages over healthy cells. Genes ATR, ATM, CHEK1, AKT1, and CDK2 are kinases, their main function is to maintain genome integrity by regulating cell cycle progression and DNA repair. Gene ATM is part of many signaling networks that include cell metabolism and growth, oxidative stress, and chromatin remodeling, all of which favor cancer progression. Gene CHEK1 is important for the initiation of cell cycle checkpoints, cell cycle arrest, DNA repair and cell death to prevent damaged of cells from progressing through the cell cycle. Gene AKT1 regulates many processes including metabolism, proliferation, cell survival, growth, and angiogenesis by phosphorylating a range of downstream substrates in response to growth factor stimulation. Gene CDK2 regulates cell progression through the cell cycle. The activity of this gene is especially critical during the G1 to S phase transition. Oncogene BRCA1 controls cellular pathways that maintain the genome stability, including DNA damage-induced cell cycle checkpoint activation, as well as transcriptional regulation and apoptosis. Oncogene HER2 responds to the growth factors and their overexpression is important for stroma-to-epithelium signaling, increasing its responsiveness and creating many ligands that originate primarily in the stroma or in the tumor cells that eventually leads to malignant growth. Oncogene MDM2 regulates the transcriptional and post-translational levels. MDM2 phosphorylation leads to changes in protein function and stabilization of p53.Tumor suppressor gene P21 mediates cell cycle arrest in the G1 phase and cell senescence in response to several stimuli, including oncogene-induced proliferation. Gene P21 is responsible for a bifurcation in CDK2 activity following mitosis, cells with high P21 enter a G0/quiescent state, whilst those with low P21 continue to proliferate. P21 may inhibit apoptosis in response to replication fork stress, however, it does not induce cell death on its own. A more detailed description on the role of each gene is presented in the Section A in S1 Appendix.

**Fig 1:**
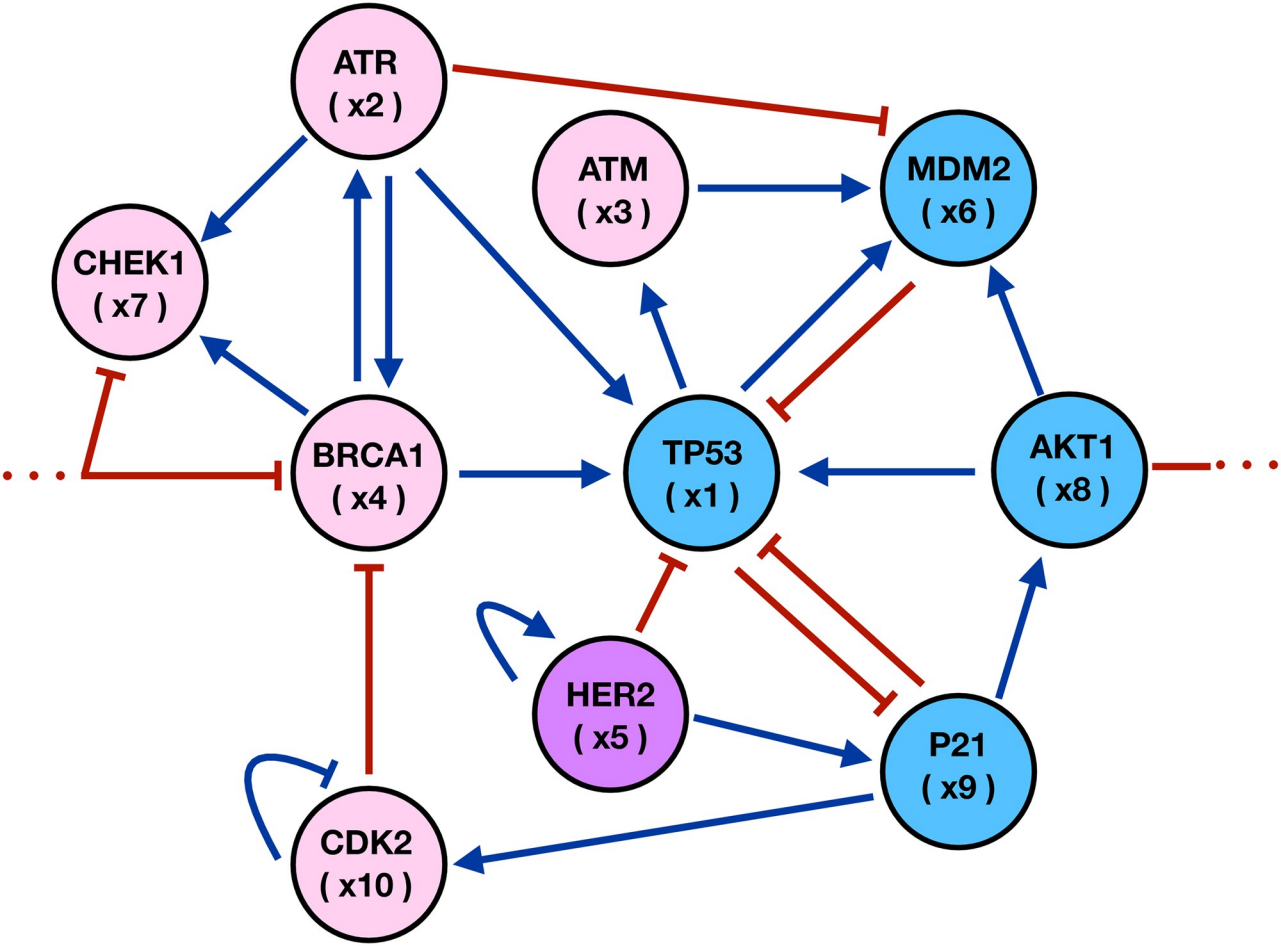
Gene regulatory network for breast cancer. Microenvironmental agents, estrogen (pink) and oxygen (blue) affect gene states transcription. The arrows indicate activation whereas the short bars represent inhibition interactions between genes.

The assembled GRN shown in Fig 1 with its regulatory links uncovers interesting macroscopic phenotypic dynamics at the physiological level such as the phenotypic equilibrium in populations of breast cancer cell lineages. The genes related to protein kinases, ATR, ATM, CHEK1, and CDK2 have a direct effect on estrogen production and on cell cycle. On the other hand, the BRCA1 and HER2 oncogenes can change their expression levels and modify the cell cycle whenever estrogen concentration gradients are established in the microenvironment. Additionally, tumor suppressor genes and oncogenes TP53, AKT1, MDM2 and P21 are associated to the oxidative stress as a result of oxygen concentration gradients in the microenvironment. The GRN includes the positive feedbacks: MDM2-TP53 which modulates metabolic regulation; ATR-TP53 that is necessary for oxidative stress; TP53-P21 which responds to pathogenesis of cancers, and HER2-TP53 that is related to the regulation of estrogen tolerance. On the other hand, assuming that the active and inactive parts of a gene state are important for the GRN dynamics, then we can write, *X*_*a*_ + *X*_*h*_ = *X*_0_ where *X*_*a*_ represents the gene active part and *X*_*h*_ is the gene inactive part, and *X*_0_ represents the full gene state. Then, the GRN evolution can be described in terms of relative variables (*X*_*a*_ + *X*_*h*_)/*X*_0_ = 1. By writing *x*_*a*_ = *X*_*a*_/*X*_0_ or equivalently *x*_*h*_ = *X*_*h*_/*X*_0_ we can express either *x*_*a*_ = 1 − *x*_*h*_ or *x*_*h*_ = 1 − *x*_*a*_ in the GRN dynamics. Therefore, the assembled GRN describes the interactions between genes as well as between genes and the microenvironmental agents. These interactions are described by the following nonlinear system of kinetic equations:

$$\begin{array}{ccc} \frac{dx_{i}}{dt} & = & \alpha_{g_{i}}(\frac{y_{i}}{\eta_{i}}-1)(1-x_{i})+R\left(x_{i};\lambda_{g_{i}};\delta_{i}\right)H\left({\tilde{x}}_{i};{\tilde{\beta }}_{i};{\tilde{\nu }}_{i};{\tilde{\gamma }}_{i}\right)\\ & + & \sum_{j}x_{i}H\left(x_{j};\beta_{j};\nu_{j};\gamma_{j}\right)-\mu_{i}x_{i}, \end{array}$$ (13)

The function *x*_*i*_ represents the state of gene *i* in Fig 1, with *i* = 1, 2, …10, while the parameters $\alpha_{g_{i}}$ represent the interaction rate constants between gene states and microenvironment and is the main temporal scale of system dynamics. The action of the microenvironmental agents estrogen and oxygen on gene activation is represented by *y*_*i*_. The quantity *η*_*i*_ represents the gene activation-inhibition (A-I) threshold parameter and *μ*_*i*_ is the self-degradation constant. The parameters of Eq (13) were estimated by constructing and analyzing the Boolean representation of the GRN. The details are presented in the Section B in S1 Appendix. In the same Section B in S1 Appendix, continuous model subsection, a summary of the specific values of these parameters together with a brief explanation about how and why these values were chosen is presented in a table already included there.

The self-regulatory positive and negative feedback genes interactions are given by the following equation,

$$\begin{array}{c} R\left(x_{i};\lambda_{g_{i}};\delta_{i}\right)=\{\begin{array}{c} \frac{\lambda_{g_{i}}}{x_{i}+\delta_{i}}x_{i},\;\text{positive}\;\text{feedback}\\ \;\;\;\;\\ \frac{\lambda_{g_{i}}}{x_{i}+\delta_{i}}\delta_{i},\;\text{negative}\;\text{feedback} \end{array} \end{array}$$ (14)

where $\lambda_{g_{i}}$ is the activation constant and *δ*_*i*_ is the activation threshold parameter. Finally, the genetic interaction strength between genes is modeled by the Hill function.

$$\begin{array}{c} H\left(x_{j};\beta_{j};\nu_{j};\gamma_{j}\right)=\beta_{j}\frac{{\left(\nu_{j}x_{j}\right)}^{\gamma_{j}}}{k_{j}+{\left(\nu_{j}x_{j}\right)}^{\gamma_{j}}}. \end{array}$$ (15)

where *β*_*j*_ is the A-I strength, *ν*_*j*_ is the activation rate at which gene *j* affects the state of gene *i*. On the other hand, *k*_*j*_ represents the threshold of the sigmoidal function, *γ*_*j*_ is the Hill coefficient which defines the steepness of the sigmoidal function representing the cooperativeness of the transcription factor that regulates the binding to the genes. In the present case *k*_*j*_ = 1/2 for all genes, this means that all genes have the same possibility to activate or inhibit each gene state. Moreover, the value of *γ*_*j*_ depends on the number of neighboring genes that affect the state of gene *j*.

*Microenvironmental action*– We consider that oxygen and estrogen are the main microenvironmental agents that affect the GRN dynamics. In this context the variables *y*_1_, *y*_6_, *y*_8_ and *y*_9_ are determined by the normalized oxygen concentrations, while the remaining ones are proportional to the estrogen expression levels as,

$$\begin{array}{c} C_{3}\left({\vec{r}}_{j},t\right)/\left(C_{3}\left({\vec{r}}_{j},t\right)+\kappa_{{}_{\epsilon }}\right), \end{array}$$ (16)

where *κ*_*ϵ*_ is one half the estrogen carrying capacity. The last equation emphasizes the presumption that changes in the GRN dynamics are the result of gene activation by estrogen concentration levels. In this case, *κ*_*ϵ*_ regulates the threshold at which genes are expressed. For the analysis wild type threshold values were considered.

*Intrinsic and extrinsic plasticity*– To understand the intratumoral diversity that drives cancer development, the known facets of the disease can be categorized into cell-intrinsic and cell-extrinsic components. Intrinsic cell components are the inherent properties of a cell that contribute to its oncogenic phenotype as a result of collective molecular changes, stochastic genetic alterations as well as selective pressures. Extrinsic cell components are features related to microenvironmental variations that influence its phenotype perturbing the course of neoplastic disease [87]. These intrinsic and extrinsic cell factors can be categorized as cell intrinsic and extrinsic plasticity [87, 88].

**Fig 2:**
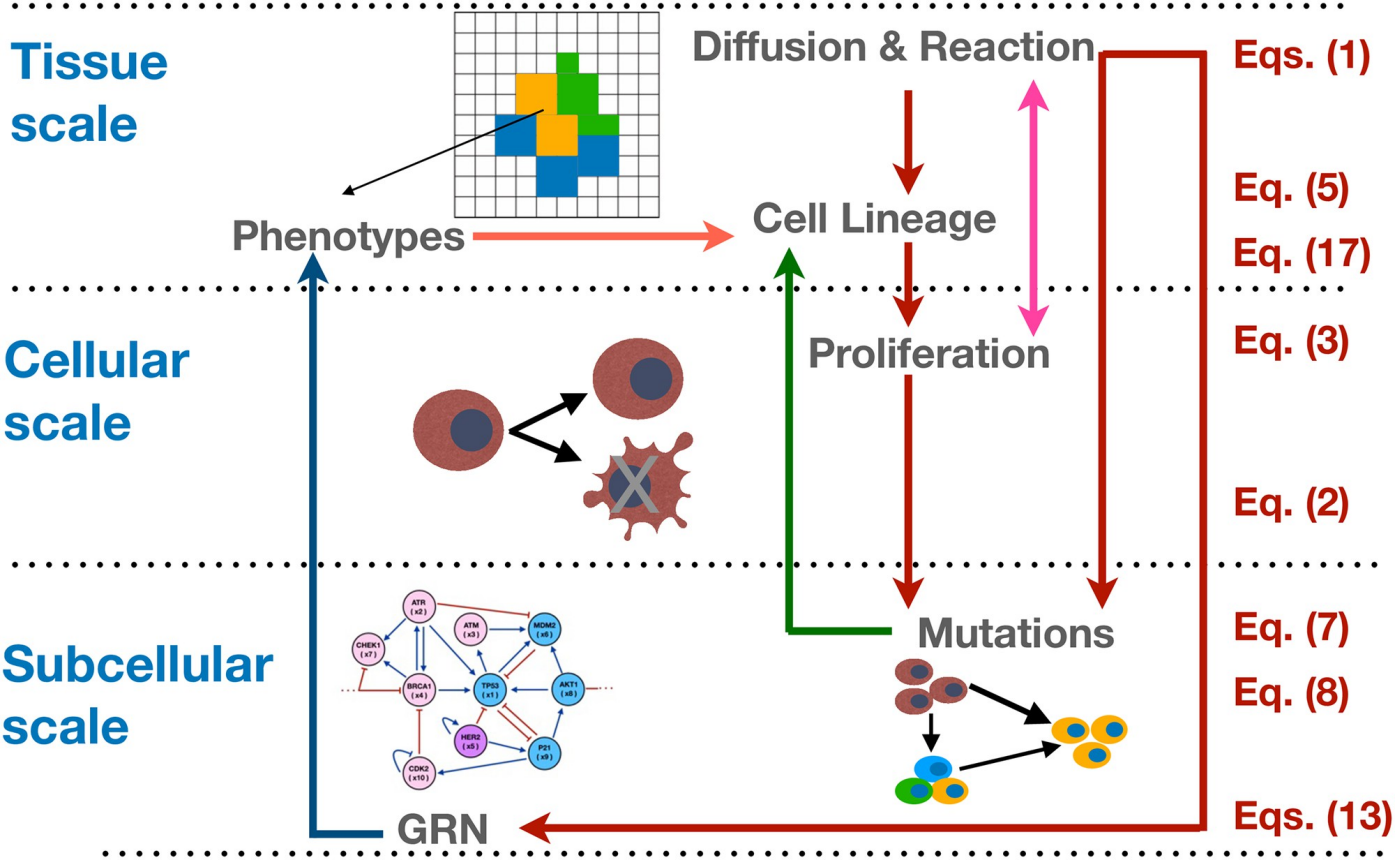
The structure of the multiscale model for breast cancer. The diagram illustrates the processes that take place at each spatial scale. The colored arrows illustrate the coupling between the scales. At each spatial scale the corresponding equations that were solved are indicated.

A cell lineage is the developmental history of a differentiated cell as traced back to the cell from which it arose. Thus, cell linage depends on the ability to active or inhibit genes as a result of these two types of plasticity. As a first estimation of cell plasticity one can propose that gene A-I is a linear superposition of the intrinsic and extrinsic plasticity. The former is proportional to the A-I parameter *η*_*i*_ while the latter is proportional to the segregation index $\hat{\beta }\left({\vec{r}}_{i},t\right)$. Thus, one can write down the following relationship for the variations of the threshold A-I parameter $\Delta \eta_{i}\left(\vec{r},t\right)$.

$$\begin{array}{ccc} \Delta \eta_{i}\left(\vec{r},t\right) & = & \eta_{i}\left(\vec{r},t\right)-\eta_{o_{i}}\left(\vec{r},t\right)\\ & = & -(\xi \left({\vec{r}}_{i},t\right)\eta_{o_{i}}\left(\vec{r},t\right)+\hat{\beta }\left({\vec{r}}_{i},t\right)\chi_{i}\left(\vec{r},t\right)), \end{array}$$ (17)

where $\eta_{o_{i}}\left(\vec{r},t\right)$ is initial threshold value, $\xi ({\vec{r}}_{i},t)$ and $\chi (\vec{r},t)$ are the intrinsic and the extrinsic (quorum sensing) susceptibilities, respectively. The minus sign in the right side of the equation indicates that there is certain opposition of the cell to change its plasticity and the minimum value of $\eta_{i}\left(\vec{r},t\right)$ is zero. Within this context Eq (17) accounts for the collective mutation changes and quorum sensing on the expression levels of a given gene. Consequently, the increase of cancer cells is the result of genetic heterogeneity which in turn increases the segregation index. Therefore, the positive feedback between cell lineage and cell proliferation modifies the development of mutations, as is indicated in the diagram of Fig 2. Eq (17) has two limits, (i) when intrinsic plasticity (linear heritage) dominates genetic dynamics so that *ξ* becomes important, and quorum sensing susceptibility becomes negligible, and (ii) when extrinsic plasticity (epigenetic inheritance) dominates genetic dynamics and segregation becomes important whereas intrinsic plasticity can be considered negligible. Here we study how the combination of both plasticities contribute to the development of breast cancer.

### Simulation details and numerical integration

The quantitative model that we analyze involves different types of nonlinear interactions that scale from the molecular level to subcelular to cellular and to tissue levels. The integration across spatial, temporal, and functional scales is highly nontrivial. Fortunately, it has been shown that multiscale computational models are very useful to gain insight into biological systems [89–92]. Many authors have used a combination of agent and cellular automata models together with ordinary and partial differential equations to model cells on the discrete scale as well as diffusible molecules on the continuous scale [93–96]. Here we implement and simulate a computational multiscale model that couples continuous, discrete and stochastic degrees of freedom that best capture tumor evolution across spatial and time scales including the positive feedbacks. The simulations describe the random mutations, cell proliferation, gene regulatory network, cell lineages and phenotypes as well as the transport of essential nutrients and estrogen during tumor evolution. The purpose of this approach is to obtain celular lineage and tumor spatial evolution as emergent properties. To this end, we introduced different positive feedbacks among some cancer hallmarks that lead to direct consequences in tumor development. To develop the numerical simulations a combination of different programming languages has been used. The complete multiscale evolution, where the equations were integrated, has been implemented in the programming language Julia version 16.1. The data processing to construct the microarray and average figures was carried out in the programming language Matlab version 2021. To characterize the attractor states of the network, we have used the Boolnet package in the programming language Rstudio version 3.6. Finally, the stability analysis of the GRN was also carried out using the programming language Rstudio language and the grind.R routine version 7-10-20. All the programming codes are allocated in github.com/jrromeroarias repository. In Fig 2, we illustrate schematically the coupling of the temporal and spatial scales of the model and the equations that were solved. The arrows among the scales indicate a dynamic update of the system variables. The GRN is crucial in this multiscale model since it contains the cell fate dynamics and the coevolution of the system. We analyzed the Boolean representation of the GRN to identify the attractors that are related with the normal, precancer and cancer phenotypes. Then, in order to comprehend the development and the occurrence of different cell phenotypes, we used the dynamics of GRN kinetic model with a combination of microenvironmental pressures to modulate the gene activation thresholds that yield the GRN Boolean attractors. A detail analysis is presented in Section B in S1 Appendix.

We consider that normal, cancer, and necrotic cells live on the sites of a 2*D* square lattice of size *L*^2^ = 500^2^ or on the sites of a 3*D* cubic lattice of size *L*^3^ = 225^3^. At the beginning of the simulations all cells are normal except for one that has developed cancer and is located at around the lattice center. We assumed that this initial cancer cell has suffered mutations in the gene TP53, which is the gene that plays a crucial role in tumor’s growth. Once this initial cancer cell begins to proliferate its descendants undergo mutations in all the other genes according to the probability distribution given in Eq (8). Nutrients, oxygen, and estrogens, were continuously supplied trough a capillary located at the top of the lattice, simulating the bloodstream. The solutions of the reaction-diffusion system, Eq (1), together with the probability of death, Eq (2) and cell cycle period for division obtained from Eq (4) were calculated numerically. Every time that a cell cycle for the cancer cell is completed, the system of Eq (13) for the dynamics of the GRN is solved.

The time scale of the Tau-Leaping Gillespie algorithm is of the order of tens of seconds while the GRN evolution happens in a temporal scale of the order of minutes. In addition, the temporal diffusion scale of essential nutrients and estrogen is at least of the order of hours. According to these time scales, essential nutrients were taken as field variables that change very slowly, so that we numerically calculated the stationary solutions of the reaction-diffusion equations and then performed the analysis of the GRN dynamics which determines kinetically the cell phenotypic states. The system of reaction-diffusion Eq (1), were integrated using zero flow boundary conditions at the left, right, and lower sides of the 2D domain and on the four vertical and bottom sides of the cubic domain. To avoid steep nutrient concentration gradients we carried out a homogenization diffusion procedure for essential nutrients by solving the equations locally. We used a grid of size 20^2^ units for 2*D* lattices, or a grid of size 20^3^ units for 3*D* lattices, with zero flow boundary conditions, and swept the lattice by randomly choosing the nodes populated with cancer cells until the steady state of the subsystem was attained. Once the homogenization procedure was carried out the reaction-diffusion equations were solved globally in 2*D* or 3*D* lattices until a simulation cycle was completed. A simulation cycle consists of a complete swap of the lattice, that is, once each site of the lattice has been visited.

Once a cell cycle is completed and cell division has happened, the daughter cell position is chosen randomly with equal probability as one of the four nearest neighbor sites in the 2*D* array or one of the six nearest neighbors sites in the 3*D* array. If the site is already occupied by a normal cell, it is replaced by the cancer cell; however, if the site is occupied by cancer cell, they pile up. Then, a random number *r* distributed uniformly in the interval [0, 1] is chosen, and it is compared with the probability $P_{rm}\left(\vec{r},t\right)$. A mutation happens whenever, $r>P_{rm}\left(\vec{r},t\right)$ otherwise no mutation occurs, afterwards, another cell is randomly chosen and the procedure for mutation dynamics described above is repeated. Because metastasis was not considered in the model, we ran simulations to estimate the time in which the tumor reached the domain border. To simulate the stochastic mutation dynamics for the probability, $P_{rm}\left(\vec{r},t\right)$ we used the Tau-Leaping Gillespie algorithm [71] which has demonstrated its usefulness in the simulations of different processes in molecular biology. In the present case we used the Tau-Leaping algorithm because the time scale of the random mutations is smaller than the time scale of cell proliferation. To figure out the statistical meaning of the results we calculated the time evolution of representative quantities of the system for 20 realizations. We found that the results for averages over 2, 4,6,…20 realizations. were very close to each other and followed a similar trend. These results will be shown in the following section with a brief discussion.

For 2*D* arrays the largest tumor size was 450^2^, whereas for 3*D* arrays the largest tumor size was 200^3^. According to this, it was found that the required time to obtain the largest tumor was *T*_*max*_ ≈ 800 simulation cycles in both, 2*D* and 3*D* lattices. By using Eq (4), one can estimate the length of a simulation cycle, *T* ≈ 12 − 15 hours. One should recall that during the cellular automaton evolution the cancer cell cycle increases as estrogen concentration grows until it reaches a value that drives the cell division. One should note that the biological cell cycle for cancer cells lasts 35 hours [97–99]. Once the biological cell proliferation happens the cellular automaton clock is reset to zero. In this way several cells can proliferate even during the cellular automaton cycle. This procedure mimics a more realistic approximation of cell proliferation. The values of the simulations parameters were chosen in accordance with [25]. That is, we considered, *θ*_d_ = 0.01, *θ*_div_ = 0.3, λ_*i*_ = {50, 100, 200} and *α*_*i*_ = {2/*L*, 4/*L*, 8/*L*}, with *L* the linear lattice size. By recalling that in the present model the probability *P*(*B*) is considered as a varying parameter, simulations were carried out for *P*(*B*) = 0.05, 0.1, 0.25, 0.5, 0.75, and 1.0. A summary of the model parameter values is presented in Table 1.

**Table 1:** Model Parameter values.

Name	Parameter	Values
Grid size in 2*D*	*L* ^2^	500^2^
Grid size in 3*D*	*L* ^3^	225^3^
Consumption rates	*α*_*i*_	{2/*L*, 4/*L*, 8/*L*}
Supply rates	λ_*i*_	{50, 100, 200}
Steepness of dead propability	*θ*_*d*_	0.01
Steepness of hopping probability	*θ*_div_	0.3
Estrogen carrying capacity	*κ*_*ϵ*_	{0.05, 0.1, 0.25, 0.5, 0.75, 1.0}
Intrinsic susceptibility	*ξ*	{0.1, 0.25, 0.5, 1.0, 2.0, 4.0, 8.0}
Extrinsic susceptibility	*χ*	{0.5, 1.0, 2.0, 4.0, 8.0, 12.0}
Local size neighborhood	*R*_*o*_	20
Probability of mutations by estrogen	*P*(*B*)	{0.05, 0.1, 0.25, 0.5, 0.75, 1.0}
Agent interaction rates	$\alpha_{g_{i}}$	{−1.0, 1.0}
Microenvironment agents	*y*_*i*_	[0, 1]
Activation-inhibition threshold	*η*_*i*_	[0.05, 1.0]
Agent self-degradation	*μ*_*i*_	0.2
Activation-inhibition strength	*β*_*i*_	{0.2, 2.0, 3.0, 9.0, 20.0}
Activation rates	*ν*_*i*_	{0.5, 1.0, 1.2, 1.5}
Agent carrying capacity	*k*_*i*_	0.5
Hill coefficients	*γ*_*i*_	{1.0, 2.0, 3.0, 4.06.0}
Agent self-activation	$\lambda_{g_{i}}$	0.4
Activation threshold	*δ*_*i*_	{0.1, 1.0}

## Results and discussion

**Fig 3:**
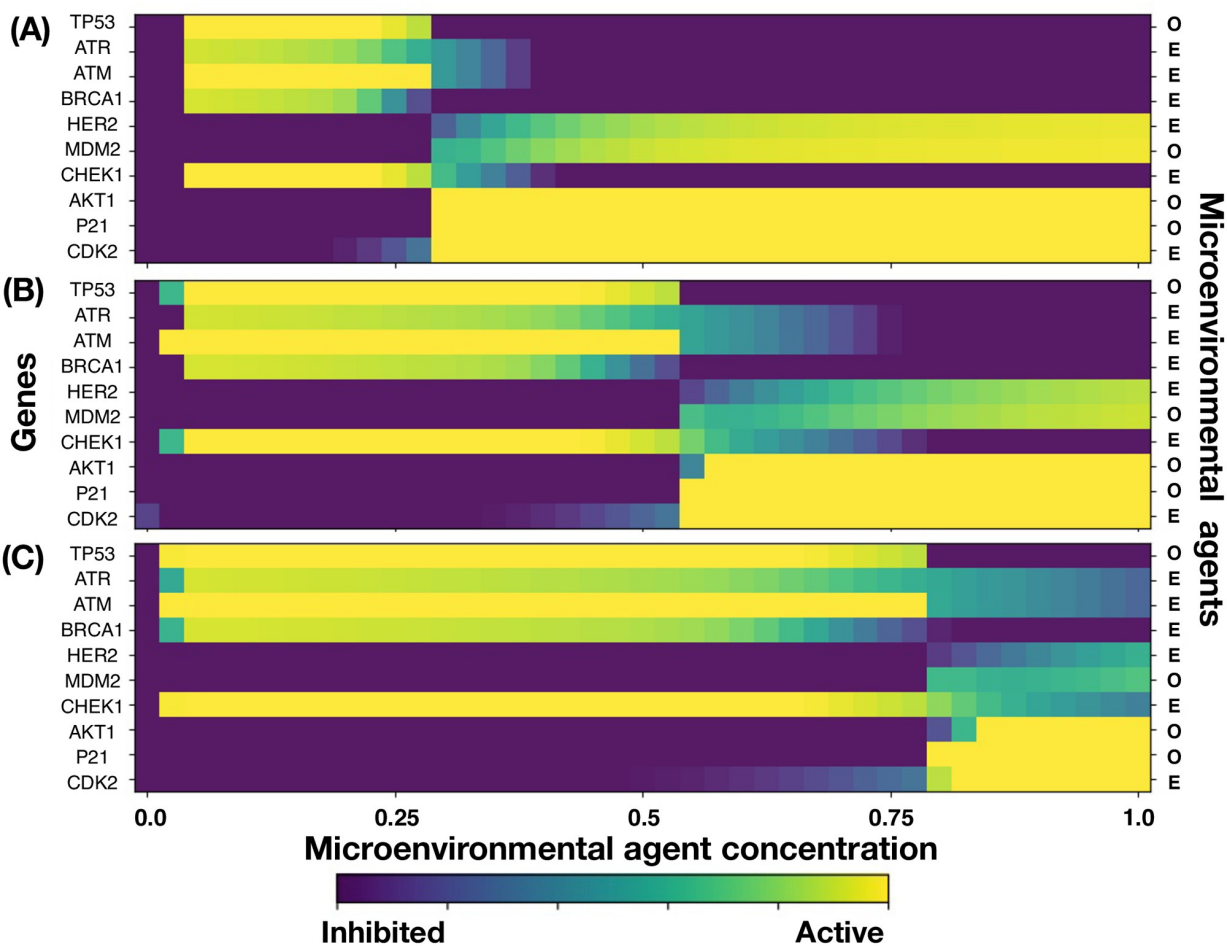
Gene expression levels in terms of the normalized concentrations of oxygen (O) and estrogen (E) for three initial values of the activation/inhibition (A-I) threshold parameters. (A) $\eta_{0_{i}}=0.25$ low, (B) $\eta_{0_{i}}=0.5$ medium, and (C) $\eta_{0_{i}}=0.75$ high.

Microarrays may be used to measure gene expression in many ways, but one of the most popular applications is to compare the expression of a set of genes from a cell maintained at a particular condition to the same set of genes from a reference cell maintained under normal conditions (wild type). Bearing this in mind, here we present our findings for gene expressions in terms of a series of microarrays that facilitate the characterization of different states of the GRN shown in Fig 1. These states were obtained from the numerical solutions of the set of differential Eqs (13)–(16) coupled to the transport Eq (1). An exhaustive analysis of the state space dynamics was carried out and the details are presented in Section B in S1 Appendix. As a starting point, we analyze the response of the GRN to three initial values of the activation-inhibition (A-I) threshold parameter: (A) *η*_*oi*_ = 0.25, low activation, (B) *η*_*oi*_ = 0.50, medium activation, and (C) *η*_*oi*_ = 0.75, high activation. The results are shown in Fig 3. The microarray left vertical axis shows the GRN genes while the right vertical axis indicates the microenvironmental agent, oxygen or estrogen, that leads to each gene expression. The microarray horizontal axis indicates the normalized concentration values of these microenvironmental agents. The minimum normalized concentration (NC) value of the microenvironmental agent needed for a gene to be expressed depends on which gene is being considered. For instance, in Fig 3A, genes HER2, MDM2, AKT1, P21 and CDK2 need a NC higher than 30% to be expressed. The NC values increase in about the same proportion as the initial A-I threshold parameter is increased. Observe that genes TP53, ATR, ATM and BRCA1 are expressed at relatively low values of the NC in a well defined concentration window which size increases as the initial A-I threshold parameter increases. Note that for $\eta_{o_{i}}=0.25$ genes AKT1, P21 and CDK2 are fully expressed for oxygen and estrogen NC values greater than 0.25, whereas for $\eta_{o_{i}}=0.50$ and 0.75 these genes are fully expressed for NC values greater than 0.50. Nonetheless, genes ATR, ATM, BRCA1, MDM2 and CHEK1, are expressed for oxygen and estrogen NC values smaller than 0.50. The expression of each gene is shown in the microarray of Fig 3B when the initial value of the threshold parameter is set at the middle saturation value $\eta_{o_{i}}=0.5$, for all genes. There, one sees that genes TP53, ATR, ATM, BRCA1, CHEK1 and CDK2 become active for oxygen and estrogen NC values less than 0.5. However, genes HER2 and MDM2 become active for estrogen and oxygen NC values greater than 0.75, respectively. Similarly, genes AKT1, P21 and CDK2 become activated for oxygen and estrogen NC values greater than 0.50. These results indicate that the first four genes dominate the GRN dynamics for $\eta_{o_{i}}=0.50$. On the other hand, the microarray of Fig 3C obtained for $\eta_{o_{i}}=0.75$ shows that genes TP53, ATR, ATM, BRCA1, CHEK1 and CDK2 become activated for oxygen and estrogens NC values less than 0.75. Nonetheless, genes HER2, MDM2, ATK1,P21 and CDK2 become activated for estrogen and oxygen NC values greater than 0.80, respectively. These results suggest that for high values of $\eta_{o_{i}}$ genes TP53, ATR, ATM, BRCA1 and CHEK1, dominate, once more, the GRN dynamics.

**Fig 4:**
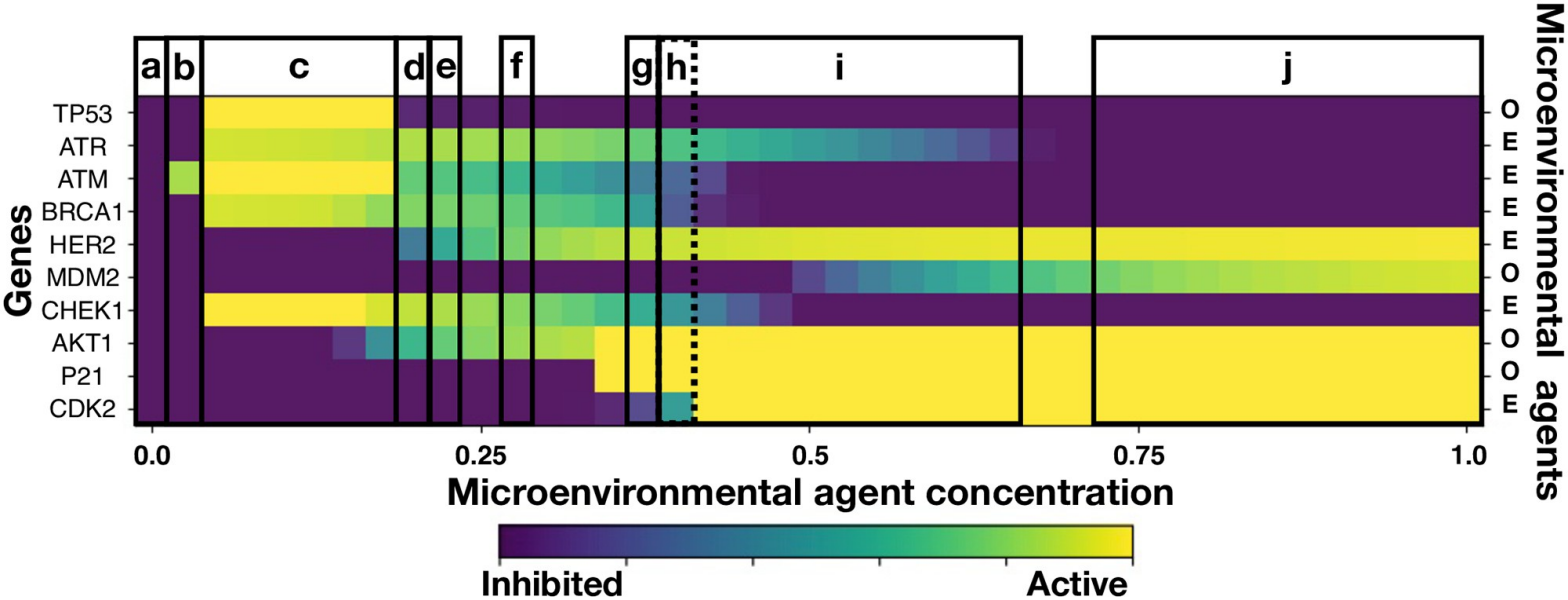
Microarray showing gene expression and cell phenotypes. Phenotypes were identified with attractors in the state space of the GRN dynamics. Normal cell phenotypes are represented by the states **a** and **c** while precancer phenotypes are represented by the states **b, d, e, f, g, h,** and **i**. Cancer phenotype was represented by the state **j**, which corresponds to the over-expression of genes HER2, ATK1, P21, and CDK2. The dotted line of the state **h** means that there is an overlap with state **i**. The initial threshold parameters for each of the ten genes from top to bottom are: *η*_*oi*_ = (0.3, 0.45, 0.15, 0.45, 0.15, 0.45, 0.3, 0.15, 0.3, 0.5). Phenotypes were identified through the analysis of the GRN boolean dynamics as well as from an analysis of the dynamics of a GRN continuous model. (For details see Section B in S1 Appendix).

Fig 4 shows microarrays associated to three cell phenotypes during cancer development: (i) normal, (ii) precancer, and (iii) cancer cells. These phenotypes were identified as attractors in the space states of the dynamics described by the GRN shown in Fig 1. GRN dynamics was analyzed with both, a continuous model, Eq (13), and a Boolean representation of the genes (see Section B in S1 Appendix) yielding consistent results. Attractor states **a** and **c** were identified with normal phenotypes because in **a** all genes are inhibited while in **c** only genes TP53, ATM and CHECK1 are over-expressed. However, attractor states **b, d, e, f, g, h,** and **i** were identified in the Boolean GRN representation with a precancer phenotype in which some genes are either over-expressed or inhibited permanently. Finally, the state **j** is the only attractor that was identified with cancer phenotype because genes HER2, ATK1, P21, and CDK2 are over-expressed. These results are in complete agreement with a previous GRN analysis [86]. On the other hand, we estimated the effect of estrogen on gene expression levels by varying proportionally the activation thresholds, *η*_*oi*_, for each gene and by making a variation of only the gene HER2 (see Section B in S1 Appendix). These results show that the precancer phenotype depend on the initial threshold value, $\eta_{0_{i}}$, associated to estrogen that occurs in gene HER2 rather than in the other genes. One observes that the variations in $\eta_{0_{i}}$ are proportional to the amount of precancer phenotypes that are represented by the length of the horizontal fringe in the microarrays. These results are in agreement with the speed up of tumorigenesis when HER2 is overexpressed as reported in Ref. [23]. They also reaffirm the importance of estrogen concentration in changing gene expression levels and the occurrence of different phenotypes as previous research has shown [17, 18, 20, 56, 76].

**Fig 5:**
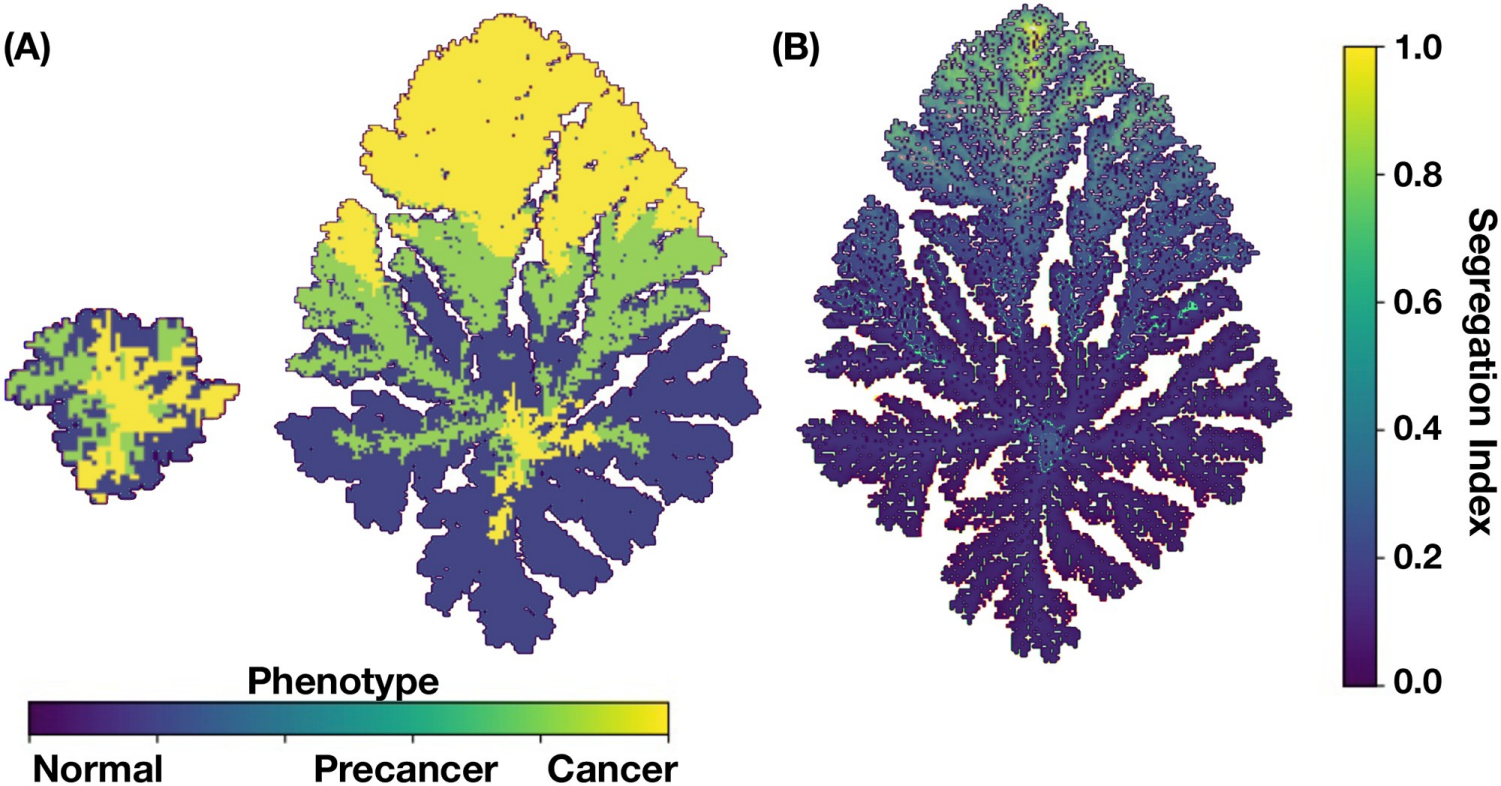
(A) Spatial distribution of cell phenotypes and (B) segregation index for a 2D tumor. These results were obtained for parameters values: *P*(*B*) = 1, *κ*_*ϵ*_ = 0.1, *ξ* = 0.5, *χ* = 4, *α*_1_ = *α*_2_ = *α*_3_ = 8 × 10^−3^, λ_1_ = 100, λ_2_ = 50, and λ_3_ = 200.

Fig 5 shows the spatial distribution of cell phenotypes obtained from simulations of a 2D tumor that incorporates the mutation dynamics described above, together with the GRN dynamics obtained with the A-I threshold values of all genes set at the medium saturation values $\eta_{0_{i}}=0.5$. The values of the kinetic parameters were carefully selected from an extensive and exhaustive analysis of the dynamics of the set of kinetic differential Eqs (13)–(16) (see Section B in S1 Appendix) so that three attractor states associated with the cell phenotypes were identified. In Fig 5A we observe tumor regions populated with: (i) cells in a normal state, purple regions, (ii) cells in a precancer state, green regions, and (iii) cells in a cancer state, yellow regions. Fig 5B shows the genetic cell lineage spatial distribution identified through their segregation index values. The color scale of the segregation index is shown on the right side of the figure. One should recall that the segregation index directly measures the spatial assortment of genetic cell lineage and ranges from 0 to 1, where 1 denotes complete lineage segregation on a given spatial scale. The segregation index $\hat{\beta }$ was calculated for 2D and 3D tumors using Eq (5) on grids of size 20^2^ and 20^3^, respectively. Notice that the tumor upper region is populated with cancer cells and it is precisely in this region where the segregation index values are greater than 0.8 as an indication of cancer cell segregation and genetic heterogeneity. On the contrary, the tumor lower region is mostly populated by normal cells with relatively low segregation index values, $\hat{\beta }⪅0.2$. Precancer cell phenotype appears for moderate values of the segregation index values, $\hat{\beta }\approx 0.25$; it is located at the middle of the tumor, occupying a smaller region as compared to the size of the regions populated with the other two cell phenotypes.

**Fig 6:**
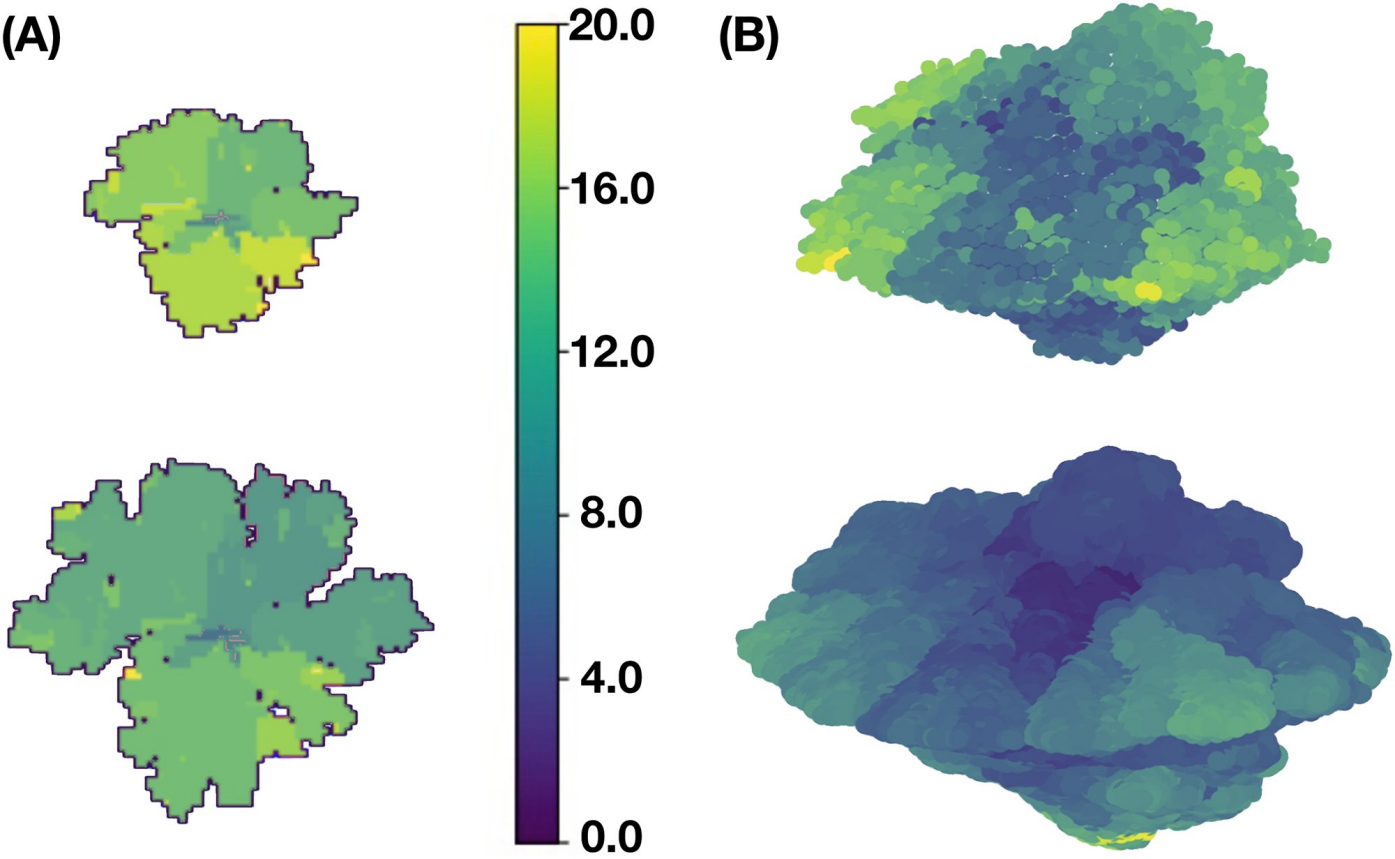
Spatial distribution of the number of mutations for (A) 2D and (B) 3D tumors. These results were obtained for the same parameters values as in Fig 5.

Fig 6 shows the spatial distribution of mutations for 2D –panel (A)– and for 3D –panel (B)– tumors at two times of their evolutionary stage. The upper figures of both panels represent tumors at about two months of development while the lower figures correspond to the same tumors after one year of evolution. Notice that cancer cells of both tumors lead a fractal-like structure at the early stages whereas they developed a solid-like structure at the later stages. This increase in fractal dimension is because the presence of cancer results in a higher cell replication rate and is consistent with recent findings [100] as well as with those found in a previous version of the present model [25].The color bar indicates the spatial distribution of the number of mutations in the cancer cells. One should also observe that genetic heterogeneity is more marked at the early stage of development as compared to the later stage. The regions where the number of mutations is maximum are relatively small and they occur at the tumor surface. These results were obtained using the same kinetic parameter values as in Fig 5.

**Fig 7:**
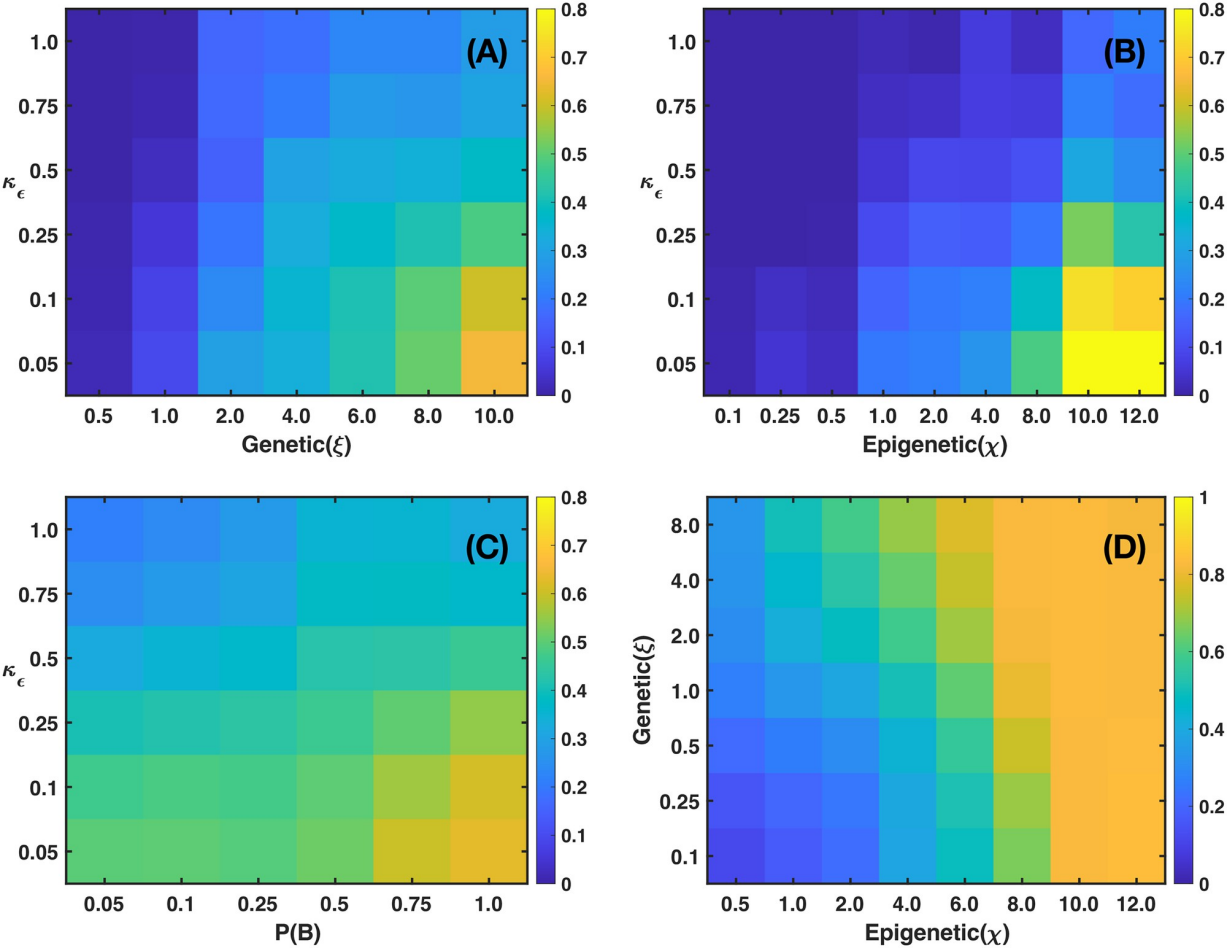
The microarrays show the fraction of cancer cells in a 2D tumor for (A) Estrogens expression levels versus genetic inheritance values. (B) Estrogens expression levels versus epigenetic inheritance values. (C) Estrogens expression levels versus estrogen receptors probability. (D) Genetic inheritance versus epigenetic inheritance. The microarrays were obtained with the following parameter values: (A) *P*(*B*) = 1 and *χ* = 0; (B) *P*(*B*) = 1 and *ξ* = 0; (C) *χ* = 4 and *ξ* = 0.5; (D) *P*(*B*) = 1 and *κ*_*ϵ*_ = 0.1. The consumption parameter values were *α*_1_ = *α*_2_ = *α*_3_ = 8 × 10^−3^, λ_1_ = 100, λ_2_ = 50, and λ_3_ = 200.

Fig 7 shows four microarrays that illustrate the fraction of cancer cells for a 2D tumor and estrogen carrying capacity *κ*_*ϵ*_ versus genetic inheritance *ξ* in panel (A), *κ*_*ϵ*_ versus epigenetic inheritance *χ* in panel (B), and *κ*_*ϵ*_ versus estrogen receptor probability *P*(*B*) in panel (C). It is also shown the genetic inheritance *ξ* versus epigenetic inheritance *χ* in panel (D). They were obtained by applying Eq (17) and from an analysis of the continuous dynamics of the GRN. See Section B in S1 Appendix. The microarray of Fig 7A shows that, for certain ranges of *κ*_*ϵ*_ and *ξ* there occurs a full estrogen expression that leads to an increasing fraction of cancer cells in the tumor. Nonetheless, for the complementary ranges of these parameters there is a full estrogen inhibition. The microarray of Fig 7B indicates that epigenetic inheritance, *χ*, follows a similar genetic heritage behavior as a function of *κ*_*ϵ*_. These results demonstrate that genetic and epigenetic inheritance may change the emergence of cancer phenotype. Because of this, one would expect that there exists a mechanism that can revert epigenetic changes. The microarray presented in Fig 7C, shows the importance of the probability *P*(*B*) of failure of estrogen receptors (ER-*α*) for different values of *κ*_*ϵ*_. This microarray suggests that if cells developed the ability to keep a high number of estrogen receptors activated, then emergence of cancer phenotype would be reduced. The microarray presented in Fig 7D suggests that the occurrence of cancer phenotype in the tumor is the result of epigenetic changes rather than genetic changes. Because of this, one may think of the possibility of decreasing epigenetic changes, and therefore, the number of cancer cells by manipulating the concentrations of microenvironmental substances –nutrients, estrogen and drugs–.

**Fig 8:**
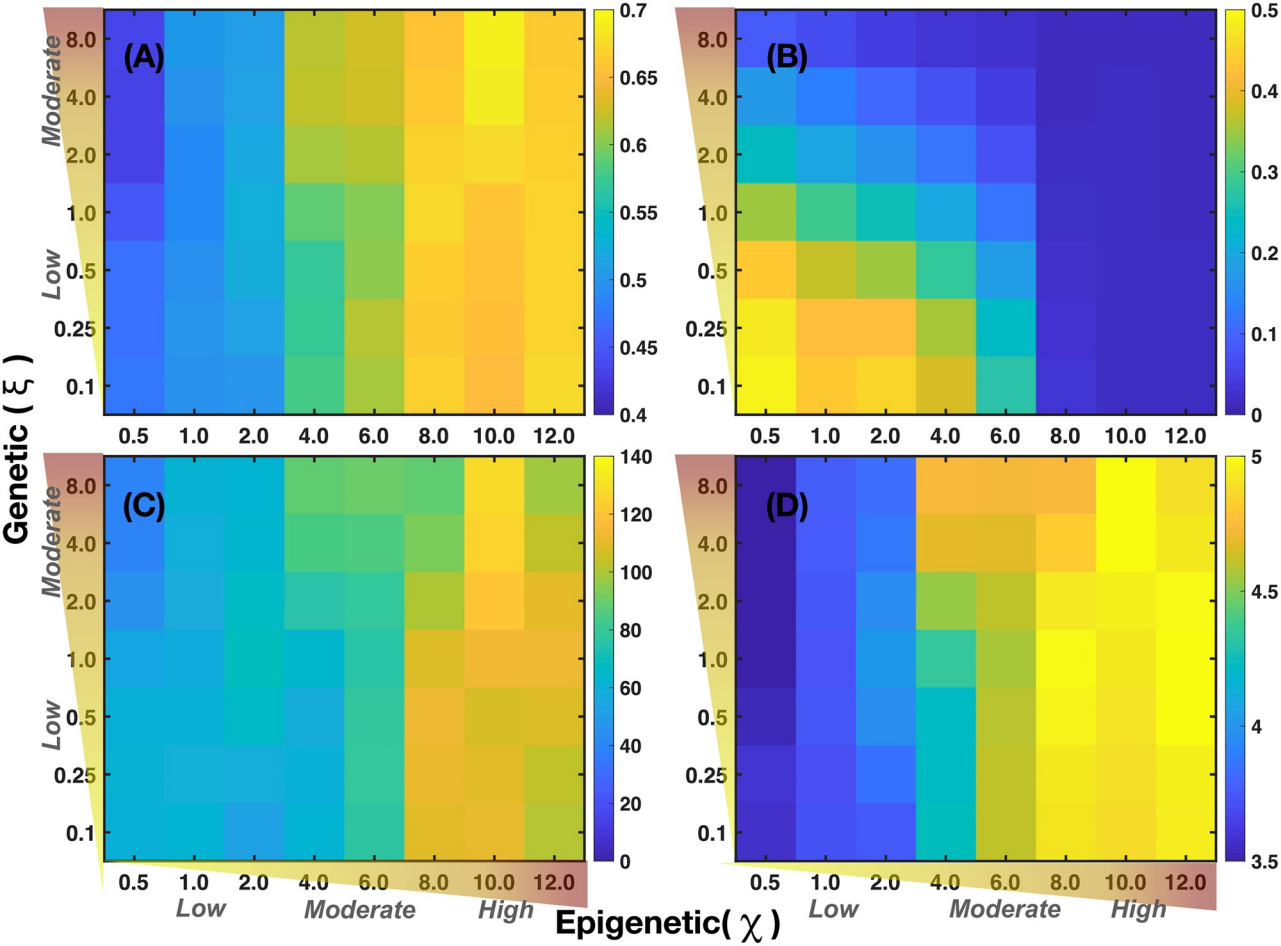
The microarrays corresponding to a 2D tumor and represent: (A) Mean segregation index. (B) Fraction of normal cells in a tumor, (C) Average number of mutations. (D) Shannon index that measures tumor heterogeneity. The axes show low to high gradient levels of the genetic and epigenetic contributions. They were obtained for the following parameter values: *P*(*B*) = 1, *κ*_*ϵ*_ = 0.1, *α*_1_ = *α*_2_ = *α*_3_ = 8 × 10^−3^, λ_1_ = 100, λ_2_ = 50 and λ_3_ = 200.

In Fig 8, the microarrays explore the importance of genetic versus epigenetic heritages in the development of 2D tumors. The microarray in Fig 8A shows the mean value of the segregation index when genetic and epigenetic inheritance change. The results of this microarray indicate that the changes in the segregation index are more susceptible to epigenetic heritages. This suggests that genetic expression thresholds are more sensible to changes in quorum sensing rather than to random mutations derived from glucose and estrogen concentration gradients. Fig 8B shows that the fraction of normal cells in a tumor is relatively high when epigenetic and genetic inheritances are small, suggesting that these inheritances play similar roles in the proliferation of normal cells. Microarrays in Fig 8C show the variations in the accumulation of mutations and heterogeneity for different values of the genetic and epigenetic heritages. One observes that epigenetic changes are more relevant for the occurrence of random mutations, in accordance to what is observed in Fig 8A. Microarray presented in Fig 8D shows the variations of the Shanon diversity index which measures intratumoral heterogeneity and can be used as a prognostic factor in breast cancer [101, 102]. The Shannon index is defined as $H=-\sum_{i}P_{i}lnP_{i}$, where *P*_*i*_ is the probability that *i* mutations occurred in the whole cancer tissue. To compute *P*_*i*_ we counted the number of cells that underwent one mutation, two mutations, three mutations, etc., and then, we divided this quantity by the total number of cancer cells. Therefore, high values of this index (H>3.5) indicate that diverse genes have underwent a relatively large number of mutations leading to genetic heterogeneity in the tumor. Genetic heterogeneity leads to the proliferation of somatic cells which favors malignant cancer phenotype [103–105]. The results shown in Fig 8D strongly indicate that variations in epigenetic inheritance define the degree of tumor malignancy rather than random mutations, in agreement with previous results [25]. These findings also suggest that it should be possible to reverse cancer phenotype by manipulating the microenvironmental agent concentrations, as suggested previously by several authors [17, 18, 20, 56, 76].

**Fig 9:**
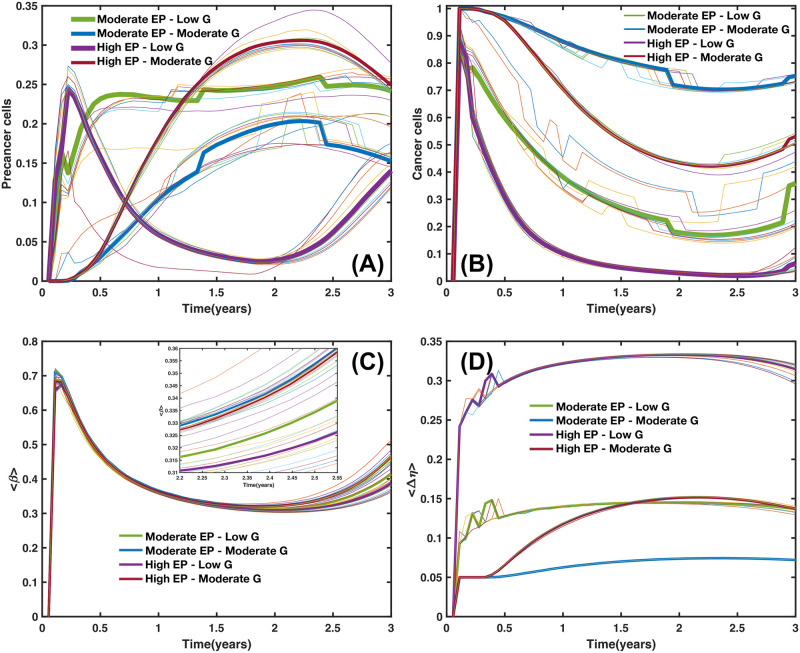
Averages carried out over various system realizations for different quantities as a function of time evolution for several values of the genetic and epigenetic inheritances parameter values that represent moderate epigenetic (Moderate EP) and high epigenetic levels (High EP) as well as low genetic (Low G) and moderate genetic levels (Moderate G). The remaining parameter values are the same as in Fig 8. (A) Percentage of precancer cells, (B) Percentage of cancer cells, (C) segregation index mean value, and (D) activation-inhibition threshold mean value for gene expression. Thin lines correspond to averages over 2,4,6…20 system realizations. Thick lines represent the averages over 10 system realizations.

In Fig 9 is shown the time evolution of different quantities averaged over 2,4,6…20 system realizations. In panel (A) is presented the percentage of precancer cells, in panel (B) is shown the percentage of cancer cells, in panel (C) is the segregation index mean value and in panel (D) the activation-inhibition threshold mean value for gene expression is plotted. These quantities were calculated for several values of the genetic and epigenetic inheritances parameters, while the remaining parameter values were kept as in Fig 8. Thin lines correspond to averages over 2,4,6…20 system realizations while thick lines represent the averages over 10 system realizations. It is observed that the average over 2 realizations follows a similar trend as the average results for 4,6…20 system realizations. This behavior suggests that the multiscale modeling yields a robust quantitative statistical representation of tumor evolution. The trend in the time evolution of the measured quantities suggests that statistical fluctuations about the average values are no significant. Therefore, there is no need to perform statistics with large samples to illustrate the behavior of the measured quantities. Panel (A) shows that by maintaining a low genetic inheritance level (*ξ* = 0.5) and varying from moderate to high epigenetic inheritance levels (*χ* = 2, 10) –green and purple lines—a minor percentage of precancer cells is obtained. On the contrary, for moderate levels of the genetic inheritance (*ξ* = 4.0) and varing the levels of the epigenetic inheritance from moderate to high levels(*χ* = 2, 10) –blue and red lines—the percentage of precancer cells increases. These results suggests that both genetic and epigenetic variations contribute significantly to the formation of precancer cells. In panel (B) we observe that by maintaining low the genetic inheritance level (*ξ* = 0.5) and varying the epigenetic inheritance values(*χ* = 2, 10) –green and purple lines—a major percentage of cancer cells is obtained. Similarly, when the genetic inheritance increases to a moderate value(*ξ* = 4.0) and the epigenetic inheritance is varied from moderate to high values (*χ* = 2, 10) –blue and red lines—the percentage of cancer cells increases. This suggests that genetic inheritance variations contribute significantly to the formation of cancer cells. In panel (C) we see that epigenetic and genetic inheritances follow a similar trend for evolution times less than two years. However, for larger times there appears an important difference since the lines representing the $<\hat{\beta }>$ tend to diverge as is shown in the inset of this panel. These results indicate that by maintaining the genetic inheritance (*ξ* = 0.5) and varying the epigenetic inheritance (*χ* = 2, 10) –green and purple lines—the segregation index $<\hat{\beta }>$ increases. Similarly, when the genetic inheritance increases (*ξ* = 4.0) and the epigenetic inheritance varies (*χ* = 2, 10) –blue and red lines—the segregation index $<\hat{\beta }>$ increases slightly. This means that the genetic inheritance contributes significantly to the tumor heterogeneity. On the other hand, the epigenetic changes can yield a reduction of heterogeneity and strongly suggest that a change from cancer to precancer phenotypes is feasible. In panel (D) we observe that by maintaining the genetic inheritance (*ξ* = 0.5) and varying the epigenetic inheritance (*χ* = 2, 10) –green and purple lines—a reduction of the activation threshold is achieved. Similarly, when genetic inheritance increases (*ξ* = 4.0) and the epigenetic inheritance is varied (*χ* = 2, 10) –blue and red lines—the activation threshold diminishes. This suggests that epigenetic inheritance conserves the activation threshold values as compared to the genetic inheritance. The results presented in Fig 9C and 9D indicate that the linear relationship proposed in Eq (17) between the intrinsic and extrinsic cell susceptibilities reinforce the idea of reducing cell genetic alterations by implementing epigenetic therapies, as we already pointed out in previous paragraphs. This is in agreement with findings reported in papers where epigenetic mechanisms are used as cancer therapies [17, 18, 20, 56, 76, 106]. Our findings clearly indicate that epigenetic variations control the activation threshold values and diminish the segregation index which lead to a decrease in the tumor heterogeneity.

**Fig 10:**
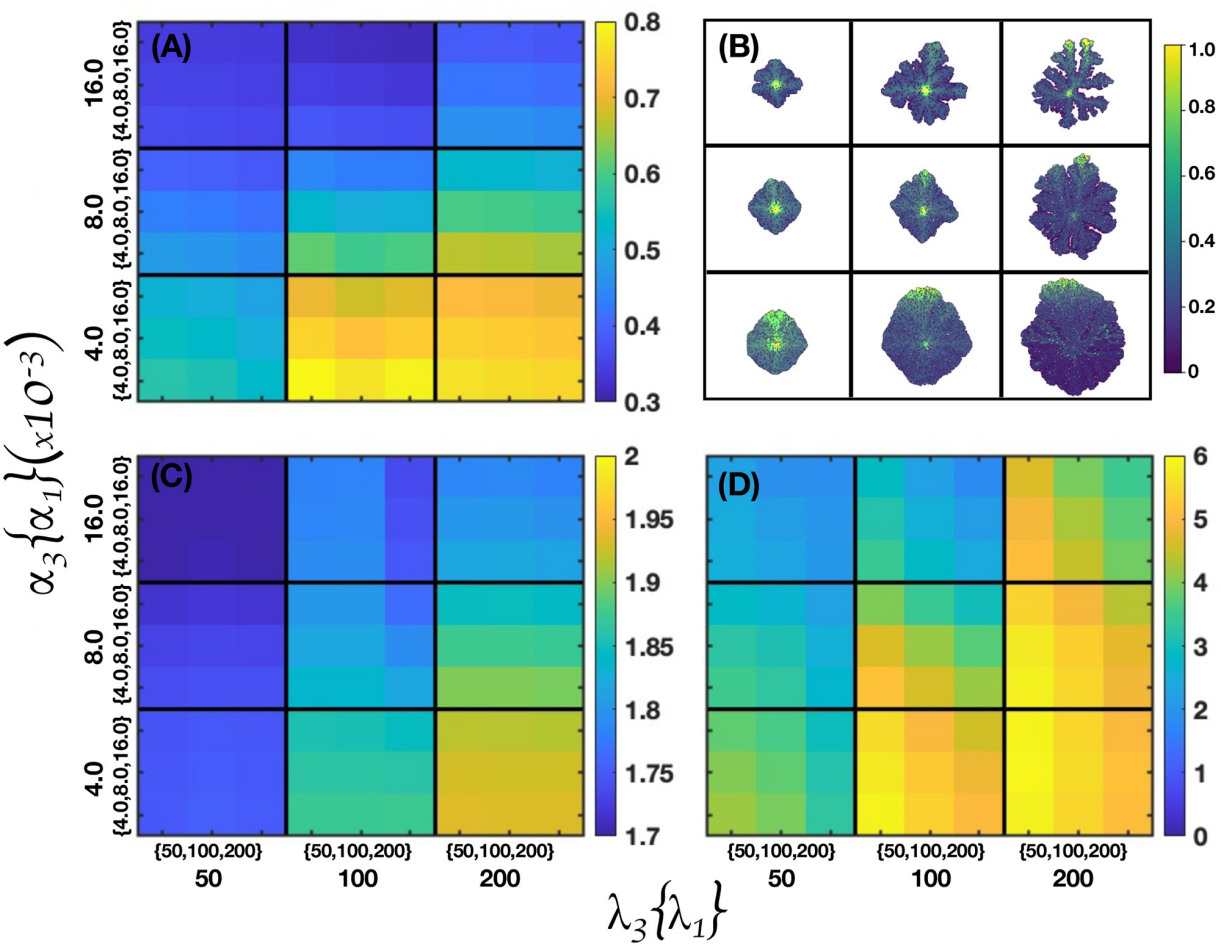
Microarrays corresponding to a 2D tumor. (A) Fraction of cancer cells in the tumor. (B) Tumor shape and spatial distribution of segregation index. (C) Fractal index, D) Shannon index. They were obtained for the following parameter values: *P*(*B*) = 1, *κ*_*ϵ*_ = 0.1, *ξ* = 0.5 and *χ* = 4.

Fig 10 shows the fraction of cancer cells, the segregation index spatial distribution, different tumor shapes, as well as the fractal and Shannon index values obtained for a 2D tumor. Microarrays (A), (C) and (D) show a main lattice (heavy lines) that indicates the estrogen consumption rate, *α*_3_ (vertical axis) and the estrogen supply rate, λ_3_ (horizontal axis). Inside each cell of the main lattice there is a sublattice that indicates the distribution of cancer cells in terms of nutrient consumption rate, *α*_1_ (vertical axis) and nutrient supply rate, λ_1_ (horizontal axis). Panel (A) shows how the fraction of cancer cells is distributed throughout the tumor. This microarray suggests that the cancer phenotype is mostly driven by estrogen consumption rather than glucose consumption. Nonetheless, the precancer phenotype happens for low (lower left square) and intermediate (centered square) values of estrogen consumption and supply rates. The normal phenotype is present for relatively high (upper left square) and intermediate (upper centered square) values of estrogen consumption and supply rates. Panel (B) shows the spatial distribution of the segregation index throughout the tumor as well as different tumor shapes that differ depending on the values of estrogen and glucose consumption and supply rates. It is observed that segregation is maximum in the regions where the estrogen gradient concentration is large. In addition, it can be observed that genetic diversity increases at the tumor center and contour. The parameter values in panel (B) correspond to the central values of the microarrays in panel (A). Note that the fractal tumor shapes shown in the third column, top and middle rows, correspond to *α*_1_ = 8 × 10^−3^, and λ_1_ = 100 and *α*_3_ = 8 × 10^−3^, 16 × 10^−3^ with λ_3_ = 200. The fractal dimension (FD) can be used to characterize the tumor structure and can be measured in histopathology slides of tissue samples using transmission optical microscopy. FD is an important quantity that is used in the diagnosis of cancer [107–110]. It has been found recently that in samples of pancreatic, breast, colon, and prostate cancer the FD increases with the progression of cancer through the different stages [100]. Because of these observations one can conclude that biological tissues in cancer progression develop intrinsic roughness. In addition, the change in texture or appearance of distortions in breast cancer tumors can be detected from mammograms by estimating the FD. In fact, it has been suggested a classification of benign and malignant tumors according to their FD values [111]. Taking these observations into account in panel (C) is shown the tumor FD distribution. It is observed that its higher values occur for *α*_1_ ∼ 4 × 10^−3^ and for 100 ≤ λ_3_ ≤ 200, pointing to a cancer phenotype, while the lower values happen for *α*_1_ ∼ 16 × 10^−3^ and for values of λ_3_ ∼ 50 suggesting the presence of a precancer phenotype. These results are consistent with those shown in panel (A) and those reported in [25]. The Shannon index distribution is shown in panel (D). The higher values of this index occur for *α*_1_ ∼ 4 × 10^−3^ and for 100 ≤ λ_3_ ≤ 200, pointing to a malignant or cancer cell phenotype. The results shown in this panel suggest that during cancer evolution the estrogen consumption is more relevant than the nutrient consumption rate which means that the increase in tumor diversity is mainly due to the presence of estrogen concentration gradients. The results in the sublattice are also consistent with those shown in panels (A)-(C) and those reported for nutrient consumption in [25].

**Fig 11:**
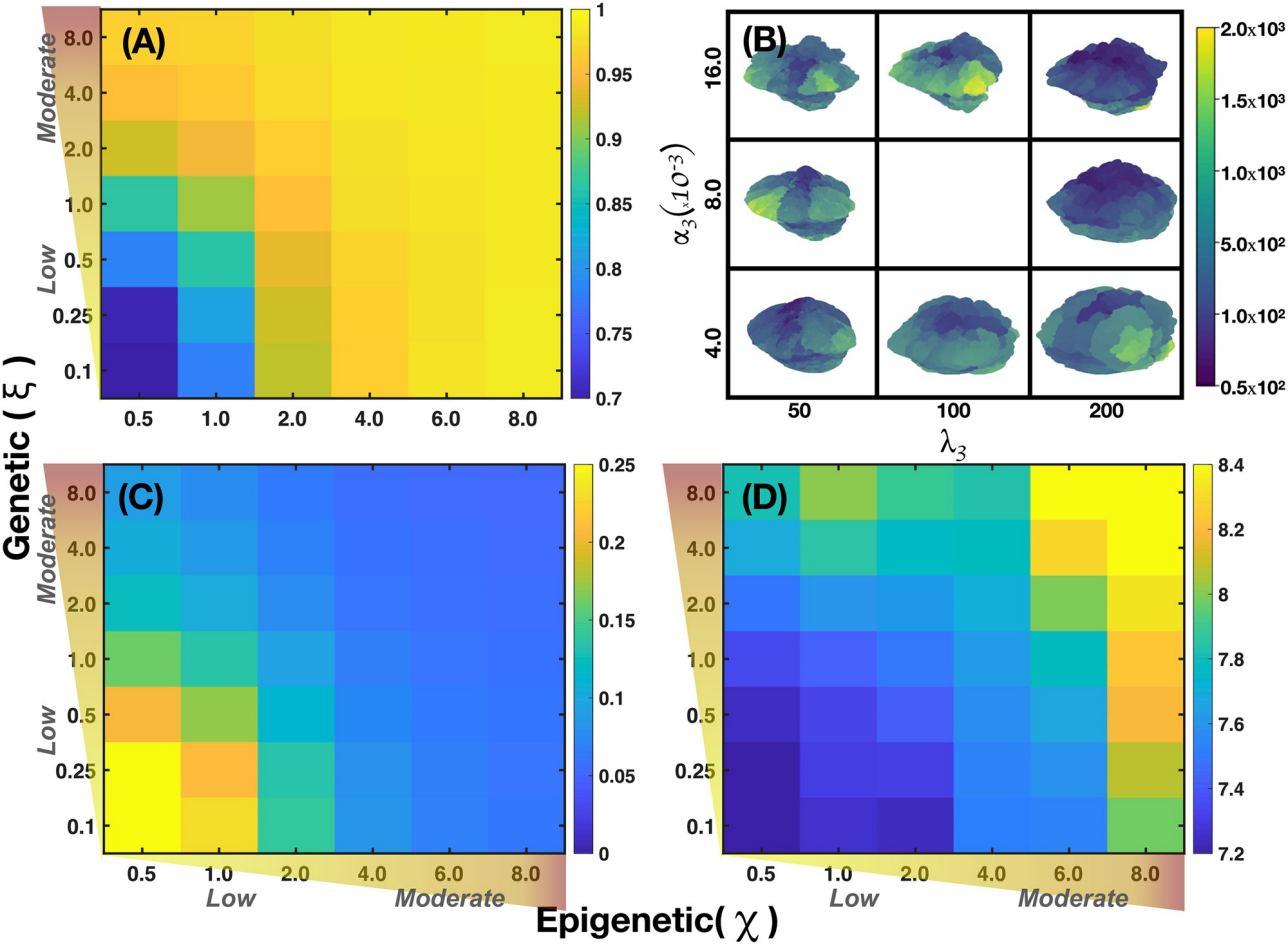
Microarrays obtained for 3D tumors that show: (A) Fraction of cancer cells in the tumor, (B) The spatial distribution of mutations for different values of *α*_3_ and λ_3_. (C) Average threshold values and (D) tumor heterogeneity. The axes show low to moderate gradient levels of the genetic and epigenetic contributions. The results shown in (A), (C) and (D) were obtained for the parameter values: *P*(*B*) = 1, *κ*_*ϵ*_ = 0.1, *α*_1_ = *α*_2_ = *α*_3_ = 8 × 10^−3^, λ_1_ = 100, λ_2_ = 50, and λ_3_ = 200.

Fig 11 presents four panels corresponding to three microarrays that explore the importance of genetic and epigenetic heritages, and the spatial distribution of random mutations in the development of 3D tumors. The microarray in Fig 11A shows that the increase in the fraction of cancer cells is correlated with epigenetic changes, suggesting that genetic expression is more susceptible to quorum sensing rather than to random mutations. Fig 11B shows the spatial distribution of random mutations in 3D tumors under different microenvironmental conditions defined by the estrogen consumption and supply rate values. There one sees that for *α*_3_ = 16 × 10^−3^ and λ_3_ = 50, 100, there are relatively small regions in the tumor where the number of mutations is of the order of one thousand. However, for *α*_3_ = 8 × 10^−3^ and 16 × 10^−3^ and λ_3_ = 200, there are large regions in the tumor where the number of mutations is of the order of hundreds. For *α*_3_ = 4 × 10^−3^ and λ_3_ = 100, 200, there are relatively large regions in the tumor that undergo between one thousand and fifteen hundred mutations. It was also found that cells that underwent more mutations were located close to the tumor surface. This result is consistent with that found for the 2D model in which most mutations were found at the perimeter of the tumor. One can also observe that the interior regions of the 2D and 3D tumors were mostly populated with necrotic cells. This is in agreement with the suggestion that HIF’s maintain cellular homeostasis and favor malignant progression leading to an increase in heterogeneity in the tumor periphery [33, 34].

The microarray in Fig 11C shows the effect of varying the genetic and epigenetic heritage threshold values whereas the panel in Fig 11D shows the distribution of the Shannon index values. It is seen that low values of the threshold value lead to an increase in mutations and as a consequence a larger Shannon index value. As seen in panel Fig 11A, epigenetic effects are more relevant, in agreement with what was found in panels (C) and (D) where a decrease in the epigenetic threshold values leads to an increase in tumor heterogeneity. By examining the features presented in Fig 8 for a 2D tumor and in Fig 11 for an equivalent 3D tumor, it can be concluded that all their characteristics are consistent with each other.

**Fig 12:**
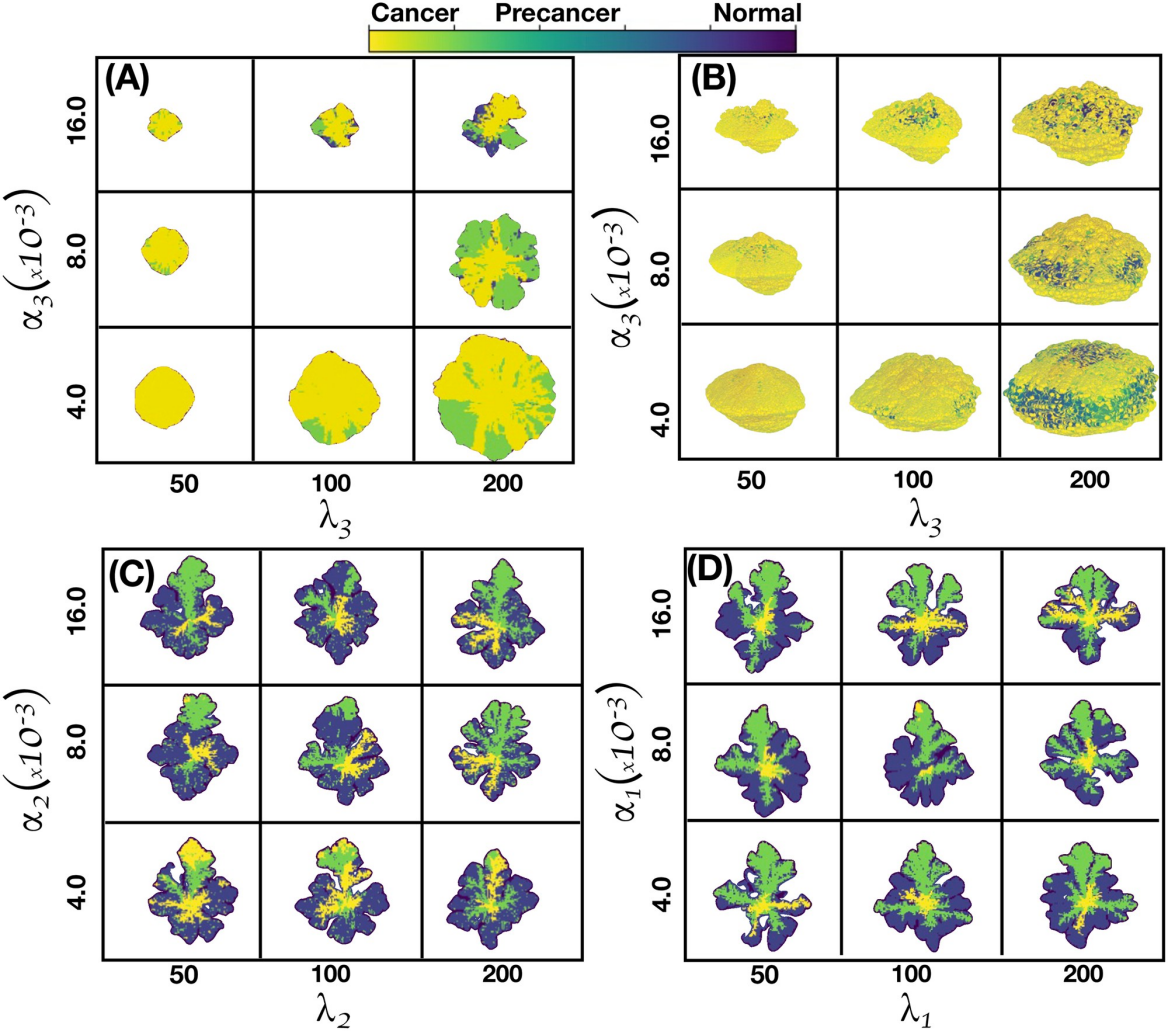
Spatial distribution of phenotypes. (A) 2D tumor and (B) 3D tumor for different estrogen consumption rate values *α*_3_ and supply rate values λ_3_. (C) 2D tumors representation for different values of oxygen consumption rate values and (D) 2D tumors for different glucose consumption rate values. In all figures we set the model parameters as in Fig 11, except in panel (C), where we set *α*_1_ = *α*_3_ = 16 × 10^−3^ and λ_1_ = λ_3_ = 100. In panel (D), we set *α*_2_ = *α*_3_ = 16 × 10^−3^ and λ_2_ = λ_3_ = 100.

Fig 12 presents the distribution of phenotypes, for a 2D tumor in panel (A) and for a 3D tumor in panel (B), for different values of the estrogen consumption *α*_3_ and supply λ_3_ rates, while the other parameters values are the same as in Fig 11. The first column of panel (A) shows that the tumor looks compact and its size increases as *α*_3_ decreases. In the second and third columns one observes that the tumor grows as *α*_3_ decreases. Precancer and cancer phenotypes can be observed as λ_3_ increases, and also the tumor develops a fractal-like structure. For the highest values of the estrogen consumption and supply rates normal, precancer and cancer phenotypes can be clearly observed. Panel (B) shows 3D tumor structures and phenotype spatial distributions for different values of the estrogen consumption *α*_3_ and supply rates λ_3_ which are consistent with those observed in panel (A). The third column corresponds to the highest value of λ_3_, and three values of *α*_3_, the distributions of normal, precancer and cancer phenotypes as well as the tumor fractal-like structure are more evident. The tumor surface is mostly populated by cancer and precancer phenotypes whereas smaller regions are populated by normal cells. In Fig 12C and 12D, we show the growth of 2D tumors by varying the consumption rates of oxygen and glucose, respectively. In panel (C) we observe that low oxygen consumption rates generate many cancer phenotypes while high oxygen consumption rates reduce cancer phenotypes and increase the production of precancer phenotypes. The previous results indicate that microenvironmental conditions with higher oxygen consumption rate favor the reduction of the amount of cancer cells. On the other hand, in panel (D) we show the influence of glucose consumption rate on tumor growth and different phenotypes formation. In all cases, we observe that higher glucose concentration leads to the spread out of the tumor mass. Also, a higher glucose consumption rate produces more cancer phenotypes as compared to low consumption rate. The combination of the panels results strongly suggests that the control of cancer phenotypes can be reached by lowering glucose and estrogen consumption rates and by increasing the oxygen consumption rate for cancer cells. Finally, note that precancer phenotype decreases while normal cells increase which indicates tumor remission could be achieved with the correct regulation of consumption rates.

### Summary

We have introduced and analyzed in detail a quantitative model that describes the growth and epigenetic evolution of an avascular tumor. The epigenetics was described through the dynamics of a GRN formed by the set of ten genes: TP53, ATM, HER2, BRCA1, AKT1, ATR, CHEK1, MDM2, CDK2, P21 that are believed to play an important role in breast cancer development. The GRN dynamics was analyzed using a Boolean representation of the genes and their interactions as well as by means of a continuous model described by ten kinetic equations that involve transcriptional positive and negative regulations. The tumor growth was simulated with a cellular automaton coupled to a set of reaction-diffusion equations that described the transport of the microenvironmental glucose, oxygen and estrogens that changed the genes expression levels. Random mutations were simulated by means of a Markovian process modeled with a master equation that involved the local concentrations of the microenvironmental agents. The role of cell segregation was also incorporated by modeling the lineage production rate –cell segregation index– with a metabolic Michaelis-Menten kinetic equation. The lineage production rate was introduced as a factor in the mutation probability associated to the genotoxic metabolites driven by estrogen concentration gradients. This approach led us to find three attractors in the GRN dynamics which are related to three phenotypes: (i) normal cells, (ii) precancer cells and (iii) cancer cells. With these ingredients the tumor structure, the spatial distribution of mutations and phenotypes for 2D and 3D tumors were calculated. From the simulations we obtained a series of microarrays that show the activation levels of each of the ten genes in response to the glucose and estrogen concentration gradients. In addition, we obtained the spatial distributions of: (i) the number of mutations, (ii) segregation index, (iii) phenotypes, (iv) shape and, (v) the Shannon index, that is a measure of the heterogeneity for 2D and 3D tumors. These quantities were also analyzed for different values of the estrogen consumption and supply rates. It was found that the regions where number of mutations maximize are relatively small and occur at the tumor surface. whereas genetic heterogeneity was more marked at the early stage of development. The segregation index spatial distribution throughout the tumor as well as the tumor shapes were different depending on the values of the estrogen and glucose consumption and supply rates. It was also found that segregation maximizes in the regions where estrogen gradient concentrations are large.

Tumors developed a fractal-like structure at the early stages whereas at later stages they tended to develop a solid-like structure. On the other hand, we studied the role of estrogen concentration in changing gene expression levels and the results reaffirm that the phenotypes can be controlled directly by estrogen concentration. All these results were found to be consistent for both, 2D and 3D tumors. The findings reported here strongly suggest that it is possible to develop epigenetic cancer treatment alternatives. Finally, we would like to emphasize that the results obtained for the fractal structure and heterogeneity of tumors are in complete agreement with those found in a previous paper [25].

## Supporting information

S1 Appendix Gene functions and epigenetic attractors. This appendix describes the functional actions of each of the ten genes that are part of the GRN for breast cancer. Also, it has a full description of Boolean and continuous GRN dynamics including multistability analysis.
(PDF)
