# Supplementary material for: A multiscale model of the role of microenvironmental factors in cell segregation and heterogeneity in breast cancer development
Source: PLoS Comput Biol. 2023 Nov 22;19(11):e1011673. doi: 10.1371/journal.pcbi.1011673 (PMC10699633; doi:10.1371/journal.pcbi.1011673)
Supplement: S1 Appendix — This appendix describes the functional actions of each of the ten genes that are part of the GRN for breast cancer. Also, it has a full description of Boolean and continuous GRN dynamics including multistability analysis. (PDF) [file pcbi.1011673.s001.pdf]

A multiscale model of the role of microenvironmental factors in cell segregation and heterogeneity in breast cancer development

J. Roberto Romero-Arias[1], Carlos A. González-Castro[2], Guillermo Ramírez-Santiago[2*]

1 Instituto de Investigaciones en Matemáticas Aplicadas y en Sistemas, Universidad Nacional Autónoma de México, Ciudad de México, Mexico
2 Instituto de Matemáticas, Universidad Nacional Autónoma de México, Juriquilla Querétaro, Mexico

* gramirez@im.unam.mx

S1 Appendix: Gene functions and epigenetic attractors

Section A. Gene functions

This section describes the functional actions (obtained from [1]) of each of the ten genes that are part of the GRN shown in Fig 1 in the main text. This set of genes is believed to play an important role in breast cancer evolution.

TP53(x_1): Tumor suppressor gene, It controls the cellular genome's integrity and is an important regulator of cell cycling, proliferation, apoptosis and metabolism. Mutations of TP53 or inactivation of its genetic product are among the first events that give rise to malignant cell transformation. The loss of control over the cell cycle, results in the acceleration of cell proliferation and facilitates metabolic reprogramming, giving the pre-malignant cells numerous advantages over healthy cells. Protein p53 becomes activated in response to countless stressors, for instance, DNA damage –induced by either UV, IR, or chemical agents such as hydrogen peroxide–, oxidative stress, osmotic shock, ribonucleotide depletion, and deregulated oncogene expression. The activation of protein p53 is characterized by two major events. (i) In stressed cells a drastic increase of the half-life of the protein p53 leads to a quick accumulation of p53. (ii) A conformational change in protein p53 drives its activation as a transcription regulator in the cell.

ATR(x_2): Kinase, It functions to maintain genome integrity by stabilizing replication forks and by regulating cell cycle progression and DNA repair. ATR is activated in response to persistent single-stranded DNA, which is a common intermediate formed during DNA damage detection and repair.

ATM(x_3): Kinase, It is a protein that coordinates DNA repair by activating enzymes that fix the broken strands. Efficient repair of damaged DNA strands helps maintain the stability of the cell's genetic information. A wealth of evidence has accumulated showing that ATM is part of many signaling networks that include cell metabolism and growth, oxidative stress, and chromatin remodeling, all of which favor cancer progression.

BRCA1(x_4): Oncogene, It controls cellular pathways that maintain the genome stability, including DNA damage-induced cell cycle checkpoint activation, DNA damage repair, as well as transcriptional regulation and apoptosis.

HER2(x_5): Oncogene, when HER2 is normally expressed, ligands that bind to the HER receptors form only a few HER2 heterodimers, and the responses to these growth factors are relatively weak, resulting in normal cell growth. However, when HER2 is over expressed as in cancer cells, many ligands originating primarily in the stroma or in the tumor cells themselves will recruit HER2 into heterodimers. The heterodimers stay longer at the cell surface, and their signaling is enhanced. This results in potent stroma-to-epithelium signaling, enhanced responsiveness to growth factors and, eventually, malignant growth.

MDM2(x_6): Oncogene, It is a key negative regulator of p53 protein and forms an auto-regulatory feedback loop with p53. MDM2 is highly regulated, the levels and function of MDM2 are regulated at the transcriptional, translational and post-translational levels. Following DNA damage, phosphorylation of MDM2 leads to changes in protein function and stabilization of p53.

CHEK1(x_7): Kinase, It is a protein kinase that has a central role in coordinating DNA damage response and cell cycle checkpoint response. Activation of CHEK1 results in the initiation of cell cycle checkpoints, cell cycle arrest, DNA repair and cell death to prevent damaged of cells from progressing through the cell cycle.

AKT1(x_8): Kinase, It is one of three closely related serine/threonine-protein kinases (AKT1, AKT2, and AKT3) that regulates many processes including metabolism, proliferation, cell survival, growth, and angiogenesis by phosphorylating a range of downstream substrates in response to growth factor stimulation.

P21(x_9): Tumor suppressor gene, It is a cyclin-dependent kinase inhibitor of the tumor suppressor p53. It mediates cell cycle arrest in the G1 phase and cell senescence in response to several stimuli, including oncogene-induced proliferation. However, p21 can be activated independently of p53 and has cancer-promoting properties, such as supporting tumor growth. P21 is responsible for a bifurcation in CDK2 activity following mitosis, cells with high P21 enter a G0/quiescent state, whilst those with low P21 continue to proliferate. P21 may inhibit apoptosis in response to replication fork stress, however, it does not induce cell death on its own.

CDK2(x_{10}): Kinase, This gene encodes a member of a family of serine/threonine protein kinases that participates in cell cycle regulation. The encoded protein is the catalytic subunit of the cyclin-dependent protein kinase complex, which regulates progression through the cell cycle. Activity of this protein is especially critical during the G1 to S phase transition.

Section B. Epigenetic attractors.

Boolean model of GRN

To identify, in the context of microenvironmental agents, how different genes interact with each other in the proposed breast cancer GRN, a Boolean network model was developed and analyzed. The interaction rules were constructed on the basis of the biophysical interactions reported by Chong Yu and Jin Wang [2] and are represented by the links depicted in Fig 1 in the main text. The Boolean functions used to analyze the network model are given in the logical Table 1.

A Boolean network model is the simplest formalism for studying the nonlinear interactions among the GRN genes and microenvironmental agents. The information that can inferred from its analysis provides a meaningful and useful qualitative description of the GRN dynamics. Because of this, this approach has been used to study different biological models [3–5]. The state of each node in the Boolean network is described by a discrete variable that takes the binary value: 0 (inhibited) or 1 (activated). In the present case, the nodes represent genes, nonetheless, they could also

Table 1. Network Boolean rules for breast cancer development.

Gene	Boolean rules
**TP53**(x_1)	$x_2 \wedge x_4 \wedge \neg\, x_5 \wedge \neg\, x_6 \wedge x_8 \neg\, x_9$
**ATR**(x_2)	x_4
**ATM**(x_3)	x_1
**BRAC1**(x_4)	$x_2 \wedge \neg\, x_8 \wedge \neg\, x_{10}$
**HER2**(x_5)	x_5
**MDM2**(x_6)	$x_1 \wedge \neg\, x_2 \wedge x_3 \wedge x_8$
**CHEK1**(x_7)	x_9
**AKT1**(x_8)	$x_2 \wedge x_4 \wedge \neg\, x_8$
**P21**(x_9)	$\neg\, x_1 \wedge x_5$
**CDK2**(x_{10})	$x_9 \wedge \neg\, x_{10}$

represent, proteins, RNA, morphogenes, etc. The links that join the nodes represent positive or negative regulations. The time evolution of the state of node x_i, is determined by the Boolean function Ψ_i that depends on the states of its regulators x_k, (with $k \neq i$), located at other nodes in the network as follows

$$x_i(t + \Delta t) = \Psi_i[x_1(t), x_2(t), ..., x_k(t)]. \quad (1)$$

The Boolean function Ψ_i is discrete and follows the algebraic rules of Boole's axiomatics. The stationary states of the dynamical mapping, called attractors, are determined by the condition $x_i(t) = x_i(t + \Delta t)$, for any $\Delta t > 0$ that lead to activation or inhibition patterns defining a homeostatic state of the system. In the present context these attractor states represent normal, precancer or cancer cellular phenotypes.

An exhaustive exploration of the attractors of the breast cancer GRN was carried out using the R BoolNet package (version 4.0.2). The Knock-Out (KO) and Over-Expression (OE) functions of each node were represented by a 0 (KO) or 1 (OE) value. The action of the OE and KO of each node was also explored. The tables that show all the identified system attractors are presented in Figs 1 and S2. Many repeating attractors were found which were classified in groups and arranged in a single table that is presented in Fig 1. These results are important because they facilitate the analysis of the GRN Boolean interactions and dynamics. Network attractors for breast cancer (see in Figs 1 and 2) can be summarized as follows: (a) The first attractor of Wild-Type character is identical for genes TP53-KO, ATR-KO, ATM-KO, BRCA-KO, HER2-KO, MDM2-KO, CHEK1-KO, AKT1-KO, P21-KO and CDK2-KO; (b) The second attractor of Wild-Type character is identical for genes TP53-KO, ATR-OE, ATM-KO, BRCA1-OE, HER2-KO, MDM2-KO, MDM2-OE, CHEK1-OE, AKT1-KO, P21-KO and CDK2-KO; (c) The third attractor of Wild-Type character is identical for genes TP53-KO, ATM-KO, HER2-KO, MDM2-KO, CHEK1-KO, AKT-KO, P21-KO and CDK2-KO; (d) The fourth, and last attractor of Wild-Type character is identical for genes TP53-KO, ATR-KO, ATM-KO, BRCA1-KO, HER2-OE, MDM2-KO, CHEK1-KO, CHEK1-OE, AKT1-OE and P21-OE; Finally, (e) the remaining attractors represent some other specific functions of some genes and are presented in the tables shown in Fig 2. The complete list of attractors is shown in the tables 4-42 at the end of this Supplementary Material. In each table, the R BoolNet package provided each attractor frequency of success. As indicated at the tables bottom, an attractor can show up with a given frequency, because of this, in some cases there is a higher probability that certain attractors appear over others; this is the reason for which the dynamics network shows only bistable states. These results are used as a guide to built up and

Wild-Type attractors						
	One state		Two states			
	Attr.(1)	Attr.(2)	Attr.(3)		Attr.(4)	
**TP53**	0	0	0	0	0	0
**ATR**	0	1	1	0	0	0
**ATM**	0	0	0	0	0	0
**BRCA1**	0	1	0	1	0	0
**HER2**	0	0	0	0	1	1
**MDM2**	0	0	0	0	0	0
**CHEK1**	0	1	0	0	0	0
**AKT1**	0	0	0	0	1	1
**P21**	0	0	0	0	1	1
**CDK2**	0	0	0	0	0	1

Fig 1. Wild Type GRN attractors (Attr) obtained from the Boolean representation.

understand the complex dynamics of a continuous model. In the next section we use the properties and information of the Boolean network attractors to tune up some of the continuous model parameter values.

TP53 - OE attractors								
	One state				Two states			
	Attr.(1)	Attr.(2)	Attr.(3)	Attr.(4)	Attr.(5)		Attr.(6)	
**TP53**	1	1	1	1	1	1	1	1
**ATR**	0	1	0	1	1	0	1	0
**ATM**	1	1	1	1	1	1	1	1
**BRCA1**	0	1	0	1	0	1	0	1
**HER2**	0	0	1	1	0	0	1	1
**MDM2**	0	0	0	0	0	0	0	0
**CHEK1**	0	1	0	1	0	0	0	0
**AKT1**	0	0	0	0	0	0	0	0
**P21**	0	0	0	0	0	0	0	0
**CDK2**	0	0	0	0	0	0	0	0

	ATM-OE				MDM2-OE		AKT1-KO					
	Attr.(3)		Attr.(4)		Attr.(3)		Attr.(3)		Attr.(4)		Attr.(5)	
**TP53**	0	0	0	0	0	0	0	0	0	0	0	0
**ATR**	1	0	0	0	1	0	1	0	0	0	1	0
**ATM**	1	1	1	1	0	0	0	0	0	0	0	0
**BRCA1**	0	1	0	0	0	1	0	1	0	0	0	1
**HER2**	0	0	1	1	0	0	0	0	1	1	1	1
**MDM2**	0	0	0	0	1	1	0	0	0	0	0	0
**CHEK1**	0	0	0	0	0	0	0	0	0	0	0	0
**AKT1**	0	0	1	1	0	0	0	0	0	0	0	0
**P21**	0	0	1	1	0	0	0	0	1	1	1	1
**CDK2**	0	0	0	1	0	0	0	0	0	1	0	1

Fig 2. Breast cancer attractors (Attr) considering Over-Expression (OE) and Knock-Out (KO) of specific genes. The upper table lists all the GRN attractors when gene TP53 is Over-Expressed (TP53-OE). On the other hand, the lower table lists the GRN attractors when genes ATM, MDM2 and AKT1 are either OE or KO.

Continuous model of GRN

Epigenetic mechanisms are essential for normal development and maintenance of tissue specific gene expression patterns. Disruption of these mechanisms may lead to altered cell functions. Because of this, epigenetic mechanisms play an important role in breast cancer development. It has been found that oxygen and estrogens distributed among

the tumor microenvironment change gene expression levels by activating or inhibiting genes which affect cell lineage [6]. To model the effect of estrogen and oxygen in the the GRN dynamics we have used the gene interaction kinetics introduced previously in [2]. –See Eqs (1) in the main text–. In this approach, the values of the state variables and parameters that control the GRN dynamics –shown in Fig 1 in the main text– vary continuously within a certain range as a result of the estrogen and oxygen concentrations gradients that lead to diverse genetic states. Here we assume that the active and inactive part of a gene state are important for the GRN dynamics, then we can write, $X_a + X_h = X_0$ where X_a represents the gene active part and X_h is the gene inactive part, and X_0 represents the gene state. Bearing in mind that that the gene state remains unchanged and is constant during the GRN evolution, then we can describe the gene state dynamics in terms of relative variables $(X_a + X_h)/X_0 = 1$. By writing $x_a = X_a/X_0$ or equivalently $x_h = X_h/X_0$ we can express either $x_a = 1 - x_h$ or $x_h = 1 - x_a$ in the GRN dynamics. This approach has been used to analyze the multistability of the dynamics of some genetic circuits [7–9]. Therefore, the temporal evolution of the state x_i of gene i is determined by its network kinetic interactions described by the following system of coupled ordinary differential equations:

$$\begin{aligned} \frac{dx_i}{dt} &= \alpha_i(\frac{y_i}{\eta_i} - 1)(1 - x_i) - \mu_i x_i + R(x_i; \lambda_i; \delta_i) H(\tilde{x}_i; \tilde{\beta}_i; \tilde{\nu}_i; \tilde{\gamma}_i) \\ &+ \sum_j x_i H(x_j; \beta_j; \nu_j; \gamma_j), \text{with } 1 \leq i \leq 10. \end{aligned} \tag{2}$$

The interaction rate constants between gene states and microenvironment are represented by the parameters α_i. The action of the microenvironmental agents on gene activation is represented by y_i while the activation-inhibition threshold parameter is represented by η_i and the self-degradation constant is μ_i. The self-regulatory positive and negative feedback gene interactions are described by the following equation,

$$R(x_i; \lambda_i; \delta_i) = \begin{cases} \frac{\lambda_i}{x_i + \delta_i} x_i, & \text{positive feedback} \\ \\ \frac{\lambda_i}{x_i + \delta_i} \delta_i, & \text{negative feedback} \end{cases} \tag{3}$$

where λ_i is the activation constant and δ_i is the activation threshold. The genetic strength is represented by the Hill function H:

$$H(x_j; \beta_j; \nu_j; \gamma_j) = \beta_j \frac{(\nu_j x_j)^{\gamma_j}}{k_j + (\nu_j x_j)^{\gamma_j}} = \frac{\beta_j}{k_j} \frac{(\nu_j x_j)^{\gamma_j}}{1 + (\nu_j x_j)^{\gamma_j}/k_j}. \tag{4}$$

where β_j is the activation-inhibition parameters, ν_j is the action rate at which gene j affects the state of gene i, the parameters k_j represent the sigmoidal threshold function, γ_j is the Hill coefficient which controls the steepness of the sigmoidal function and represents the cooperativeness of the regulatory transcription factor binding to genes. One should note that the Hill's function rewritten as in the second part of Eqs (4) suggests that the cooperativeness rate is regulated by the parameter k_j. Finally, the value of γ_j depends on the number of neighbor genes that contribute to the system cooperativeness to change the state of gene j. The system of differential Eqs (2) can be rewritten in terms of the dimensionless variables: $\hat{x}_i = \frac{\nu_i}{\hat{k}_i} x_i$ and $\hat{\delta}_i = \frac{\hat{k}_i}{\nu_i} \delta_i$, where $\hat{k}_i = k_i^{1/\gamma_i}$. Then, the dimensionless equation is written as

$$\begin{aligned} \frac{d\hat{x}_i}{dt} &= \alpha_i(\frac{y_i}{\eta_i} - 1)(\frac{\hat{k}_i}{\nu_i} - \hat{x}_i) - \mu_i \hat{x}_i + \lambda_i \tilde{\beta}_i R(\hat{x}_i; 1; \hat{\delta}_i) \hat{H}(\hat{\tilde{x}}_i; 1; 1; \tilde{\gamma}_i) \\ &+ \sum_j \beta_j \hat{x}_i \hat{H}(\hat{x}_j; 1; 1; \gamma_j), \text{with } 1 \leq i \leq 10, \end{aligned} \tag{5}$$

where $\hat{H}(\hat{x}_j; 1; 1; \gamma_j) = \hat{x}_j^{\gamma_j}/(1 + \hat{x}_j^{\gamma_j})$. This approach suggests that the stability of the system depends of fewer parameters. From this analysis we observe that there is a set of 52 parameters that are basic, but are not fully independent, that determine the dynamics system. However, this set of parameters can be further reduced if one considers the relevant time scales as: the gene interactions (β_i, λ_i) and degradation of proteins (μ_i), the coexistence between the active and inactive parts of the genes (κ_i), and the unitary action rate at which a given gene affects others $(\nu_i = 1)$. This leaves us with six free parameters to determine the multistability of the system which can be studied by following the standard bifurcation analysis. We also performed an extensive bifurcation analysis for the temporal scales represented by the parameters (α_i) and the threshold activation values (η_i) that are crucial to obtain the cellular phenotypes. In the following sections, we build up a multistability diagram of the dynamics by estimating the values of these parameters.

Parameters estimation

The analysis of the dynamics described by Eqs (2) was carried out by setting the temporal scale in terms of the parameters α_i since it is the main temporal scale that defines the interactions between relative gene states and microenvironment. For simplicity, we assumed that the dynamics of all GRN genes occurs at the same time scale, that is, $\alpha_i = \alpha_0$. Because of this, we set $\alpha_0 \pm 1$, where the positive value corresponds to a gene activation and the negative value corresponds to an gene inhibition. The local concentrations of estrogen and oxygen, are represented by y_i, and vary in the interval $[0, 1]$. One should recall that the transport of these microenvironmental agents occurs by diffusion and it is described by the set of reaction-diffusion Eqs (1) in the main text.

The microenvironment action thresholds, η_i, are chosen following some phenotypic plasticity rule and take values between 0 and 1. In this case, the values of η_i depend on the metabolic and genetic activity in the neighborhood of the cell and are measured with a cooperative enzymatic function. The relation of the intrinsic and the extrinsic plasticities with the microenvironment action thresholds, η_i is given by Eq (14) in the main text. To obtain the gene inhibition parameters values, μ_i, we first considered that the average lifetime of a protein and its transcription time is 20 minutes [10, 11] and that the average time of protein degradation is about 3 to 6 minutes [12, 13]. Thus, taking the proportion of these times and the values of α_i, it was found that the self-degradation rates, μ_i, should take values between 0.15 and 0.3. We chose the intermediate value $\mu_i = 0.2$ for all genes.

By assuming that the activation and inhibition of the GRN genes drive the system behavior, it is reasonable to consider that the values of the activation-inhibition parameters are of the same magnitude as the self-degradation rates.Thus, assuming a balance between these quantities and the degradation, all the kinetic parameters take the value $\beta_i = 0.2$, except for some of them that are related to the most outstanding multistability control circuits in the GRN [14–18], in which case the parameter β_i may change. On the other hand, to guarantee that the negative or positive self-regulation preserves the state of the gene, the value of the λ_i should double the value of β_i. Taking into account that only two genes HER2 and CDK2 present self-regulation, one can set $\lambda_5 = \lambda_{10} = 0.4$ and the remaining are set to zero. For breast cancer, it has been found that HER2 tends to remain active and overexpressed during cancer development [19–22], while CDK2 is partially degraded [2, 23]. It does not degrade completely because it must keep the cell cycle initiation pathway active [23, 24]. Therefore, we chose the values $\delta_5 = 1$ and $\delta_{10} = 0.1$. This choice of these values yields overexpression of HER2 and keeps CDK2 in a latent state.

Taking into account that the increase in estrogen receptor failure may be associated with a cooperative phenomenon [20, 22] the self regulation of gene HER2 was multiplied

by a Hill function. In this case, the Hill function models the gene affinity with itself and the possible binding sites under the cooperative action among other genes. Thus, we chose the values $\tilde{\beta}_5 = 1$, $\tilde{\nu}_5 = 0.5$ and $\tilde{\gamma}_5 = 1$. In the case of gene CDK2 no evidence has been found that there exist a self-cooperative mechanism that inhibits to itself. Because of this, the Hill function associated with CDK2 self regulation is $H(\tilde{x}_{10}; \tilde{\beta}_{10}; \tilde{\nu}_{10}; \tilde{\gamma}_{10}) = 1$. In all other cases, the capacities associated with the Hill functions are the same and take the value $k_j = 0.5$. This latter value guarantees that both, the active and the inhibit states of the gene have the same probability of being achieved. The Hill's functions vary in the range $0 \leq H(x_j; 1; \nu_j; \gamma_j) \leq 1$ when the activation-inhibition parameters β_j are normalized by the values of k_j as can be seen in the Eq (4). This allows us to analyze the dynamics without writing the equations in terms of dimensionless variables. Finally, the cooperativeness is quantified with the Hill coefficients by counting the number of input interactions that each gene has in the GRN (see Fig 1 in main text). Thus, we chose the values: $\gamma_2 = \gamma_3 = \gamma_5 = \gamma_8 = 1$; $\gamma_9 = \gamma_{10} = 2$; $\gamma_4 = \gamma_7 = 3$, $\gamma_6 = 4$ and $\gamma_1 = 6$.

For many genes in the GRN it is poorly understood how a variation of the action rate constant ν_i values modify their state. However, it is known that for some circuits, such as the TP53-MDM2-P21 the values of the action rate constants are crucial for the GRN behavior. Because of this, the action rate constants were chosen as $\nu_j = 1$ for most of the cases. Given the relevance of genes TP53, MDM2 and P21 in the appearance of cancer cells and apoptosis processes the values of these parameters were $\nu_1 = 1.5$, $\nu_6 = 1.2$ and $\nu_9 = 0.5$. [8, 14–18, 24]. These values take into consideration the fact that the TP53 agent, which is considered the guardian of the genome, has a greater influence than the other genes. Moreover, it has been shown [8, 14, 24] that the TP53-MDM2 circuit influences the network equilibrium states and cell fate, where the affinity of breaking the activation-inhibition of MDM2 over TP53 is estimated to be 80%. In addition, these kinetic values guarantee that in the TP53-P21 circuit the apoptosis process is not completed until the CDK2-P21 pathway is activated, as has been shown in [10, 24]. The choice of $\nu_9 = 0.5$ limits P21 processes while the value $\nu_1 = 1.5$ speeds up processes related to TP53.

Some studies have shown that genes can undergo changes in the average transcription factors of up to two orders of magnitude due to the action of some microenvironmental agents [24, 25]. In particular, it has been demonstrated that the transcription levels of MDM2 could be increased by up to two orders of magnitude [24]. This suggests that the TP53 circuit should speed up its transcription to maintain network stability. Furthermore, it is known that variations in the transcription levels of MDM2 should compensate to maintain CDK2 activity [8, 24], since it is essential for the control of the other parts of the network. Thus, the MDM2 and CDK2 parameter values were chosen to be $\beta_6 = 2.0$ and $\beta_{10} = 2.0$, one order of magnitude larger than the initial values, $\beta_i = 0.2$, indicated above. (See Table 2 for a summary of parameter values).

It is known that an excess of estrogen in the microenvironment increases the possibility of cancer development, since it does not only modify the cell cycle but also alters some signaling pathways [19–22]. Therefore, the magnitude of parameter values that drive the HER2 gene should be the largest in the network, so that, we set $\beta_5 = 20$. On the other hand, the HER2-TP53-P21 circuit participates in DNA repair, cell cycle arrest, and apoptosis [2]. Thus, it is expected that the HER2 repression towards TP53 and the activation of HER2-P21 must be compensated. Because of this, the TP53 activation parameter was set to about half of that corresponding to HER2 gene, that is, $\beta_1 = 9$, and the activation of gene P21 was set at $\beta_9 = 3$. These values are sufficient for the activation of P21, without affecting the expression of the gene TP53. A summary of the values of all parameters involved in Eqs (2) is presented in Table 2.

Table 2. Gene expression parameter values for the continuous model.

**Parameter(P)**	Value	**P**	Value	**P**	Value
α_1	-1.0	β_9	3.0	γ_7	3.0
α_2	-1.0	β_{10}	2.0	γ_8	1.0
α_3	-1.0	ν_1	1.5	γ_9	2.0
α_4	-1.0	ν_2	1.0	γ_{10}	2.0
α_5	1.0	ν_3	1.0	μ_j	0.2
α_6	1.0	ν_4	1.0	k_j	0.5
α_7	-1.0	ν_5	1.0	λ_5	0.4
α_8	1.0	ν_6	1.2	λ_{10}	0.4
α_9	1.0	ν_7	1.0	δ_5	1.0
α_{10}	1.0	ν_8	1.0	δ_{10}	0.1
β_1	9.0	ν_9	0.5	$\tilde{\nu}_5$	0.5
β_2	0.2	ν_{10}	1.0	$\tilde{\nu}_{10}$	1.0
β_3	0.2	γ_1	6.0	$\tilde{\gamma}_5$	1.0
β_4	0.2	γ_2	1.0	$\tilde{\gamma}_{10}$	1.0
β_5	20.0	γ_3	1.0	$\tilde{k}_5$	0.5
β_6	2.0	γ_4	3.0	$\tilde{\beta}_5$	1.0
β_7	0.0	γ_5	1.0		
β_8	0.2	γ_6	4.0		

Multistability

To analize the multistability of the dynamical system described by Eqs (2), we used a set of different algorithms described in [7, 9, 26–28]. They involve the analysis of two dimensional sections of the phase space as well as a numerical continuity approach by varying the values of the system parameters to find out the system states and their stability, that is, the basins of attraction in phase space . To this end we used the R-script Grind.R (`https://tbb.bio.uu.nl/rdb/grind.html` & `https://tbb.bio.uu.nl/rdb/grindR/grind.R` version 7-10-20). This script uses a powerful R routines package to analyze the solutions of a system of ordinary differential equations (ODEs). The results of this intensive and extensive analysis yielded all attractors as well as wild-type, KO and OE gene states that were found with the Boolean model. In Figs 3 and 4, are shown the evolution of a normal cell genetic states for different values of the microenvironmental threshold parameter η_i. It was found that for $\eta_i < 0.5$, with $i = 1, 2, ...10$, the genetic states are conserved, while for $\eta_i > 0.5$ there appear basins of attraction that are identified with precancer and cancer phenotypes. We also carried out a detailed analysis to estimate the values of the other parameters that led to a precancer phenotype. We found that for each one of the gene states the activation threshold value, should be in the range $0 < \delta_i < 0.8$. These results are in complete agreement with most of the thresholds activation values found in [2], where a stochastic model was analyzed and the thresholds were expressed as percentages.

On the other hand, it was found that for each one of the states, gene inhibition was obtained for a threshold value $\eta_i = 0$, whereas gene activation was obtained for $\eta_i > 0.8$. In Table 3, are identified the GRN Boolean attractors with those obtained with the continuous model. Gene activation/inhibition threshold parameter values are also indicated in Table 3. In the first column is listed each GRN gene whose expression leads to one of the three phenotypes that were identified as attractors in the dynamics of the set of Eqs (2), namely, (i) normal, (ii) precancer, and (iii) cancer.

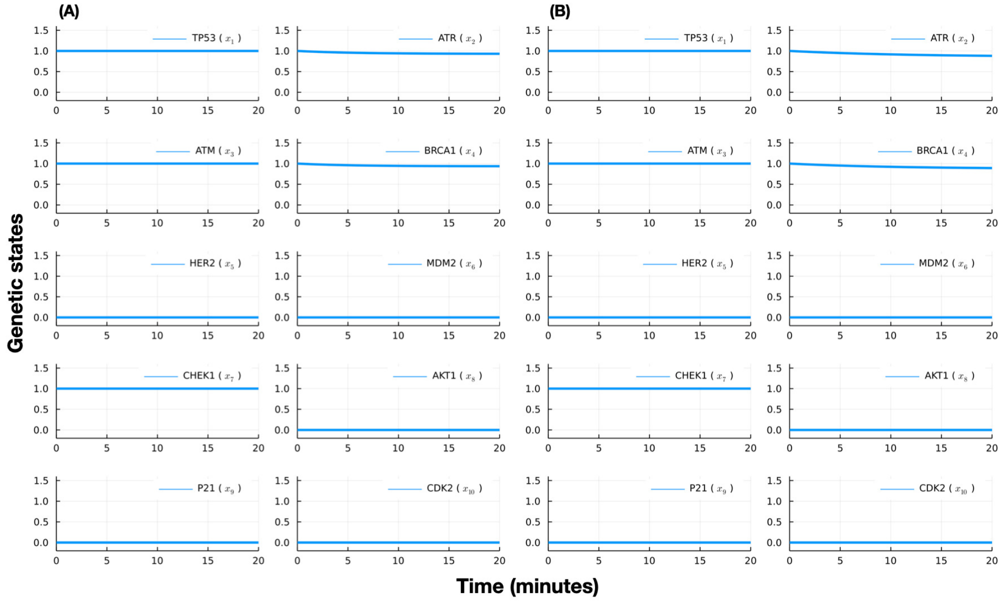

Fig 3. Evolution of genetic states for different values of the microenvironmental agent threshold values (A) $\eta_i = 0$ and (B) $\eta_i = 0.25$, with $i = 1, 2, ...10$. It starts with a genetic state that corresponds to a normal phenotype and ends with the same state.

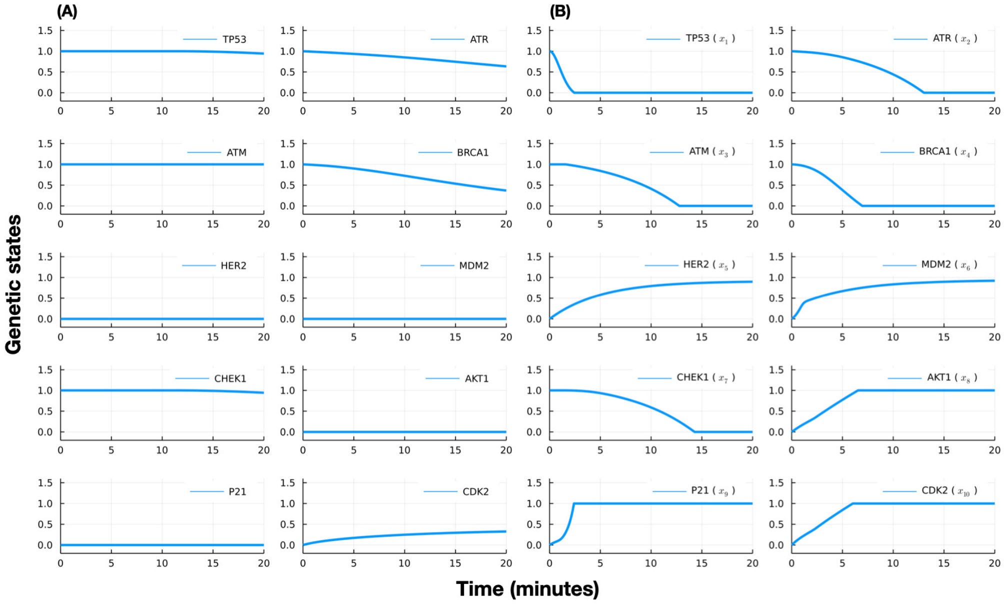

Fig 4. Genetic states evolution for different microenvironmental agent threshold values. (A) $\eta_i = 0.5$ and (B) $\eta_i = 0.75$ with $i = 1, 2, ...10$. It begins with a genetic state that corresponds to a normal phenotype and ends with precancer or cancer phenotype. We have considered that gene Over-Expression(OE) remains at value equal to one while Knock-Out (KO) stays at zero value. The sudden change in the genetic states behavior is originated from the OE and KO threshold values.

To analyze the multistability properties of the genetic states, variations and perturbations of the parameters were carried out. It was found that the stability

Table 3. Summary of gene states for Boolean and continuous models

	Boolean model		Continuous model		
**Gene**	Cancer	Normal	Cancer	Pre-cancer	Normal
**TP53(x1)**	0	1	0	<0.8≤	1
**ATR(x2)**	0	1	0	<0.8≤	1
**ATM(x3)**	0	1	0	<0.8≤	1
**BRAC1(x4)**	0	1	0	<0.8≤	1
**HER2(x5)**	1	0	1	≥0.8>	0
**MDM2(x6)**	1	0	1	≥0.8>	0
**CHEK1(x7)**	0	1	0	<0.8≤	1
**AKT1(x8)**	1	0	1	≥0.8>	0
**P21(x9)**	1	0	1	≥0.8>	0
**CDK2(x10)**	1	0	1	≥0.8>	0

depends on the activity of genes TP53(x_1)-MDM2(x_6), TP53(x_1)-P21(x_9) and ATR(x_2)-BRCA1(x_4), that undergo positive feedback in the GRN. Stability changes occurred only in some cases as will be described in the following paragraphs.

Fig 5 shows a two dimensional (2D) projection of the phase space trajectories onto the plane x_1-x_6 of the TP53(x_1)-MDM2(x_6) positive feedback equilibrium states. To obtain this projection, in Fig 5A we have considered variations of the coordinates x_1 and x_6 and have kept constant the values of the remaining variables x_i. This projection shows the points where the nullclines intersect identifying the steady states of the system obtained for parameters values fixed at $y_i = 0.2$ and $\eta_i = 0.5$ with $i = 1, 6$. The stability was determined by calculating the Jacobian and the corresponding eigenvalues. The squares represent unstable states while the circles correspond to stable states. Fig 5B shows the equilibrium points in a 2D projection of the phase space trajectories onto the plane x_1-x_6, and have kept constant the values of the remaining variables x_i, and chose a set of parameter values that led to a normal phenotype. Similarly, Figs 5C and 5D show the equilibrium points in a 2D projection of the phase space trajectories onto the plane x_1-x_6, that are identified with precancer and cancer phenotypes, respectively. The values of microenvironmental concentrations and threshold values were held at $y_i = 0.2$ and $\eta_i = 0.5$, for $i = 1, ...10$.

Figs 6A-6D show the projections of the phase space trajectories onto the plane x_1-x_6 to demonstrate how the stability of a normal cell genotype changes for different values of the concentrations of microenvironmental agents and the activation threshold. In Fig 6A the equilibrium states correspond to a normal genotype. For the concentration values of the microenvironmental agents $\eta_i = 0.25$ with $i = 1, ...10$, the unstable and stable states that occurred at $x_1 \approx 0.25$ and $x_1 \approx 0.35$, disappeared, while the unstable state at $x_1 = 1$, remains invariant as shown in Fig 6B. On the contrary, when the concentration values of the microenvironmental agents double their value ($y_i = 0.4$, with $i = 1, ...10$), the unstable state that occurred at low values of x_1 disappeared while the stable and unstable states at $x_1 \approx 0.35$, and at $x_1 = 1$, remain invariant as shown in Fig 6C. As the activation threshold is increased up to a value $\eta_i = 0.75$, the distance between the first unstable state and the stable state decreases, however, the unstable state at $x_1 = 1$ remains invariant again. These results demonstrate how the multistability of the system depends on the values of the parameters associated to the genetic and epigenetic characters of the system.

Figs 7-11 show the multistability diagrams where stable states are indicated with red

lines while unstable states are indicated with blue lines. They were obtained for parameter values that led to a normal cell phenotype. In what follows it is shown that multistability maintains under small perturbations. Fig 7 shows four multistability diagrams $x_1 - y_1$, $x_1 - \eta_1$, $x_6 - y_1$, and $x_6 - \eta_1$, for the genetic states of the TP53(x1)-MDM2(x6) circuit with parameters that led to a normal phenotype. Fig 8 also shows four multistability diagrams $x_1 - \beta_1$, $x_1 - \beta_6$, $x_6 - \beta_1$, and $x_6 - \beta_1$ for the genetic states of the TP53(x1)-MDM2(x6) circuit with parameters that led to a normal phenotype. As in previous figures the blue lines represent unstable states while the red lines correspond to stable states. These states correspond to the circles (stable state) and squares (unstable state), respectively, at the intersection of the nullclines shown in Fig 5B. Fig 9 shows the stability of the $TP53(x_1) - MDM2(x_6)$ circuit bifurcation diagram as a function of kinetic rate constants ν_1 and ν_6 for a normal phenotype. It was found that the stability range is short as indicated by the red lines. However, in the instability parameter region there may appear molecular processes such as inhibition of apoptosis or DNA damage that can change the cell phenotype. Fig 10 shows the ranges of Hill coefficients values $\gamma_i \geq 2$ for which the cooperative effect contributes to the system stability. Finally, as a preamble to future work, in Fig 11 the stability of the system is visualized as a function of the parameters α_i, with $i = 1, 6$. These analysis show that asynchronous interactions, $\alpha_i \neq \pm 1$, may modify the stability of the system.

In Fig 12-16 are shown the results of a similar analysis for the TP53(x_1)-P21(x_9) circuit. The analysis of the circuit stability led to similar findings as those described above. The bifurcation diagrams show one stable and one unstable states represented by two separate lines. This suggests that the TP53-MDM2 circuit contributes more to the GRN stability than the TP53-P21 circuit. Finally, in Fig 17, the ATR(x_2)-BRCA1(x_4) circuit is analyzed. In this case there is only one stable state which represents a normal phenotype. Therefore, it can be concluded that regardless of the parameter values the stability of the circuit ATR(x_2)-BRCA1(x_4) is robust.

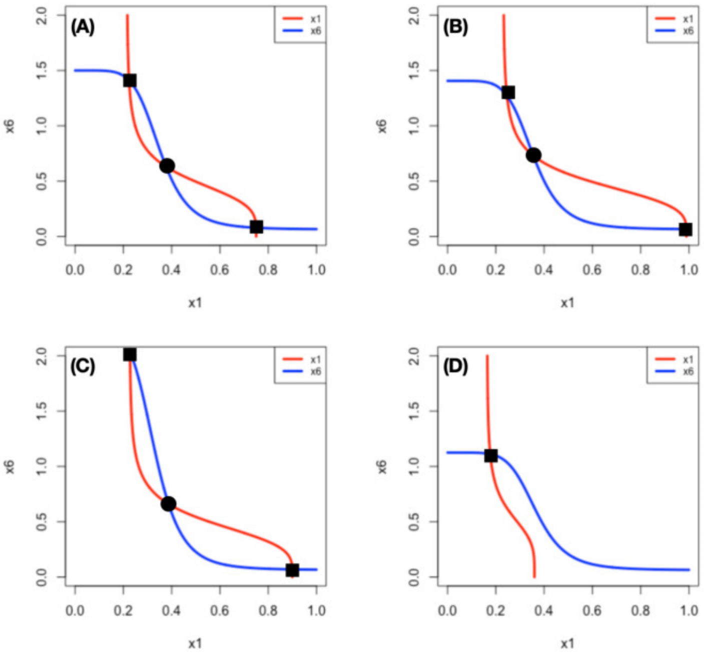

Fig 5. Projection of the phase space trajectories onto the $x_1 - x_6$ plane for the TP53(x_1)-MDM2(x_6) positive feedback equilibrium states for microenvironmental concentration values, $y_i = 0.2$ and thresholds values $\eta_i = 0.5$, with $i = 1, ...10$. The red and blue lines represent the nullclines of trajectories x_1 and x_6, respectively. Unstable states are represented by squares and stable states by the circles. (A) Trajectories $x_1 - x_6$ obtained by maintaining the other variables at a zero constant value. (B) Phase space trajectories onto the plane $x_1 - x_6$ and have kept constant the values of the remaining variables x_i, and chose a set of parameter values that led to a normal phenotype. (C) As in Fig (B) the trajectories correspond to a precancer phenotype, (D) Same as in previous panels but for a cancer phenotype.

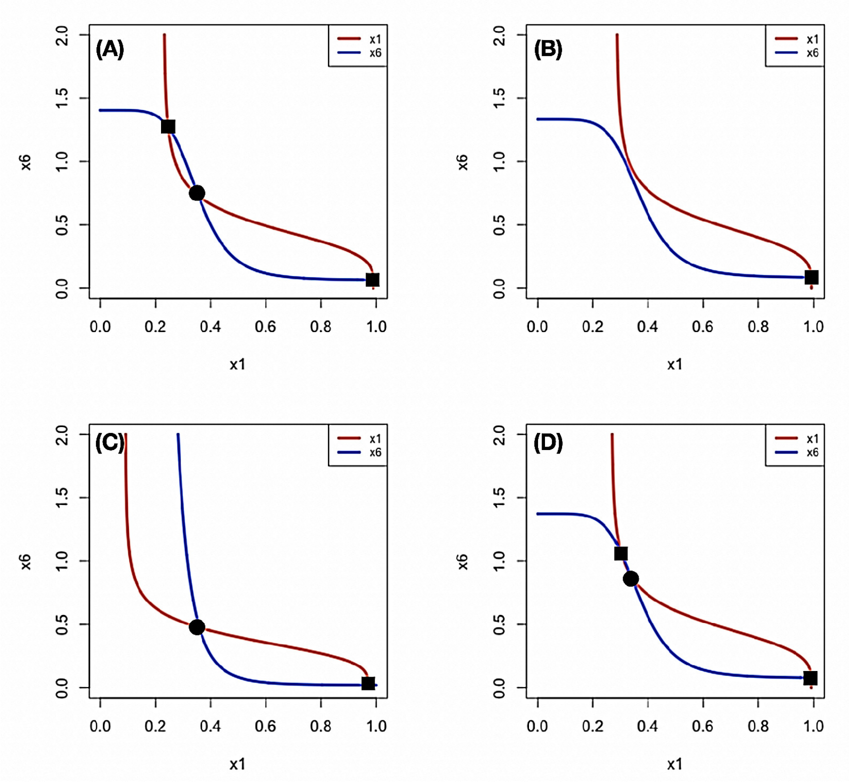

Fig 6. Phase diagrams x_6 versus x_1 for the TP53(x_1)-MDM2(x_6) circuit for a normal phenotype for different combination values of microenvironmental agent y_i and activation threshold parameter η_i. (A) $y_i = 0.2$ and $\eta_i = 0.5$, (B) $y_i = 0.1$ and $\eta_i = 0.5$, (C) $y_i = 0.4$ and $\eta_i = 0.75$, and (D) $y_i = 0.2$ and $\eta_i = 0.75$ with $i = 1, ...10$, in the four panels. Unstable states are represented with squares while the stable with circles. The red line represent the nullcline for variable x_1 and the blue line the nullcline for x_6.

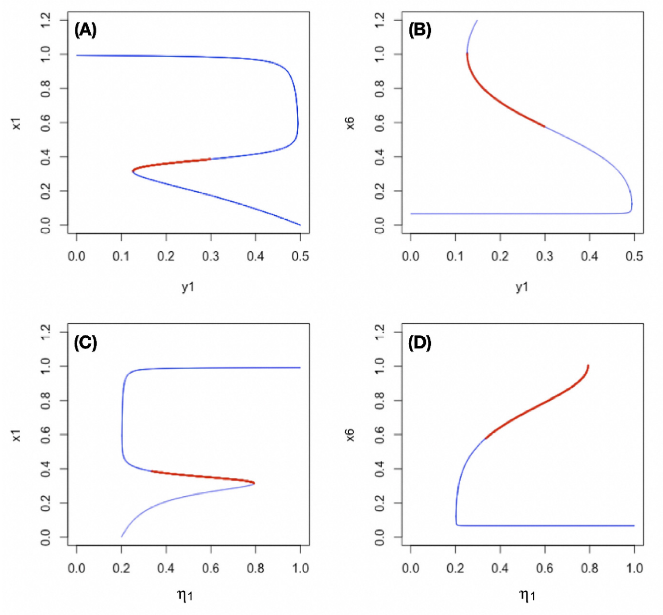

Fig 7. Multistability diagrams for the genetic states of the TP53(x_1)-MDM2(x_6) circuit for a normal phenotype. Panels (A) and (B) show the genetic states x_1 and x_6 as a function of microenvironmental agent y_1. Panels (C) and (D) show the genetic states x_1 and x_6 as a function of activation threshold parameter η_1. The blue lines indicate unstable states while the red lines represent stable states.

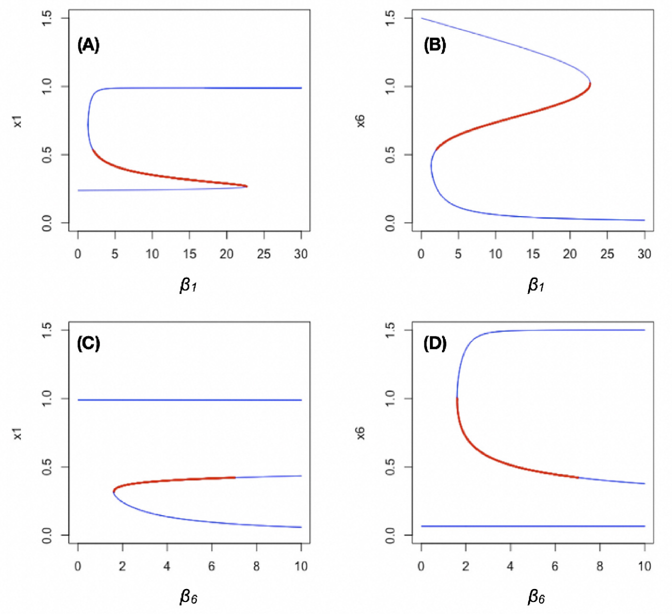

Fig 8. Multistability diagrams for the genetic states of the TP53(x_1)-MDM2(x_6) circuit for a normal phenotype, as a function of the β_1 parameter, panels (A) and (B), and β_6 parameter, panels (C) and (D). The blue lines indicate unstable states while the red lines represent stable states.

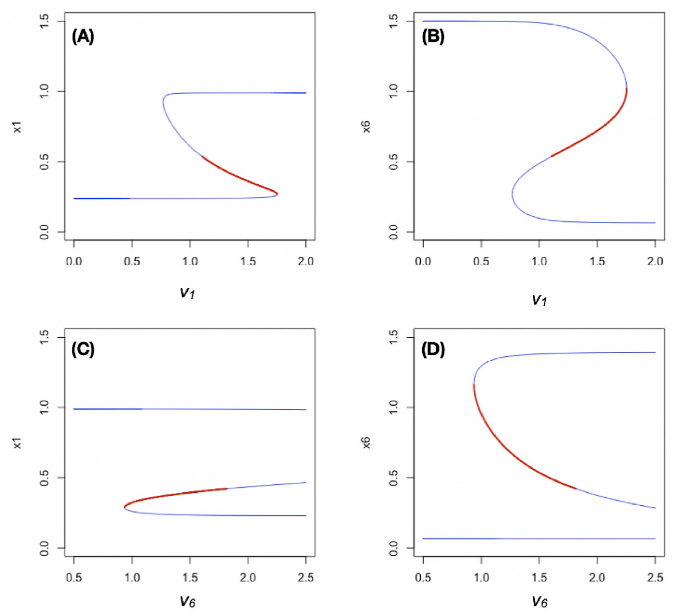

Fig 9. Multistability diagrams for the genetic states of the TP53(x_1)-MDM2(x_6) circuit for a normal phenotype, as a function of kinetic rate constants ν_1, panels (A) and (B) and ν_6, panels (C) and (D). The blue lines indicate unstable states while the red lines represent stable states.

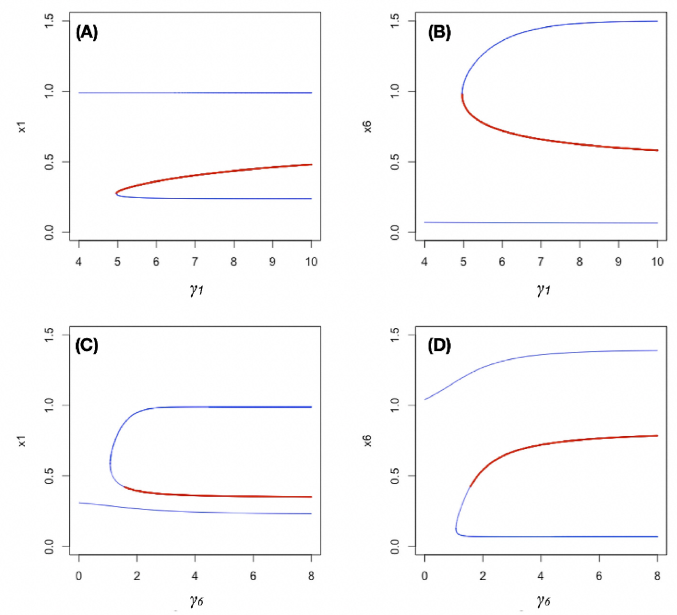

Fig 10. Multistability diagrams for the genetic states of the TP53(x_1)-MDM2(x_6) circuit for a normal phenotype. The blue lines indicate unstable states while the red lines represent stable states. (A) x_1 versus Hill coefficient γ_1. (B) x_6 versus γ_1. (C) x_1 versus γ_6 and (D) x_6 versus γ_6.

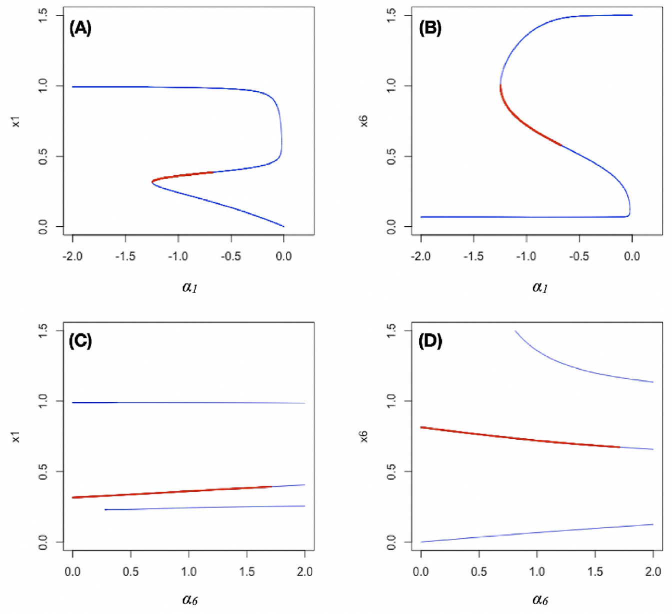

Fig 11. Multistability diagrams for the genetic states of the TP53(x_1)-MDM2(x_6) circuit for a normal phenotype. The blue lines indicate unstable states while the red lines represent stable states. (A) x_1 versus the interaction rate α_1. (B) x_6 versus α_1. (C) x_1 versus α_6 and (D) x_6 versus α_6.

Fig 18 shows a microarray that represents the attractor states or phenotypes obtained from the dynamics of the GRN continuous model shown in Fig 1 in the main text. An exhaustive and sensitive parameter analysis of the continuous model was carried out to reproduce the GRN Boolean attractors shown in Figs 1 and 2. It was found that the initial threshold values parameters η_{oi}, indicated in the horizontal scale, led to precancer phenotypes as shown in Fig 18 . Attractor states **a** and **c** can be identified with normal phenotypes, whereas attractor states **b, d, e, f, g, h,** and **i** can be identified with a precancer phenotype. This precancer identification is based on the fact that some genes are either over-expressed or inhibited permanently in the Boolean GRN representation (see Figs 1 and 2). Finally, the state **j** is the only attractor that can be identified with a cancer phenotype.

The microarrray shown in Fig 19 demonstrates the effect of increasing estrogen threshold values on the expression of all GRN genes. The initial threshold values η_{oi}, with $i = 1, ...10$ were increased in the following percentages: 10%, 25%, 50%, 75% and 100%, with respect to the values
$\eta_{oi} = (0.3,\ 0.45,\ 0.15,\ 0.45,\ 0.15,\ 0.45,\ 0.3,\ 0.15,\ 0.3,\ 0.5)$. It is observed that the gradual increase of η_{oi} changes de proportion of phenotypic states suggesting that the GRN is more adaptable to increasing values of η_{oi}. The microarray presented in Fig 20 shows the effect of increasing the estrogen initial threshold value η_{o5} in gene HER2 as indicated in the figure caption. By comparing these microarrays it is found that the HERB2 initial threshold value η_{o5} favors the a precancer phenotype. By going from top to the bottom in the microarrays we note that the increase in η_{o5} reduces the proportion of precancer phenotypes and favors the cancer phenotype. These results are consistent with findings reported in [29] where it was found that the OE of HER2 leads to cancer phenotype and accelerates tumorigenesis as well as the occurrence of different

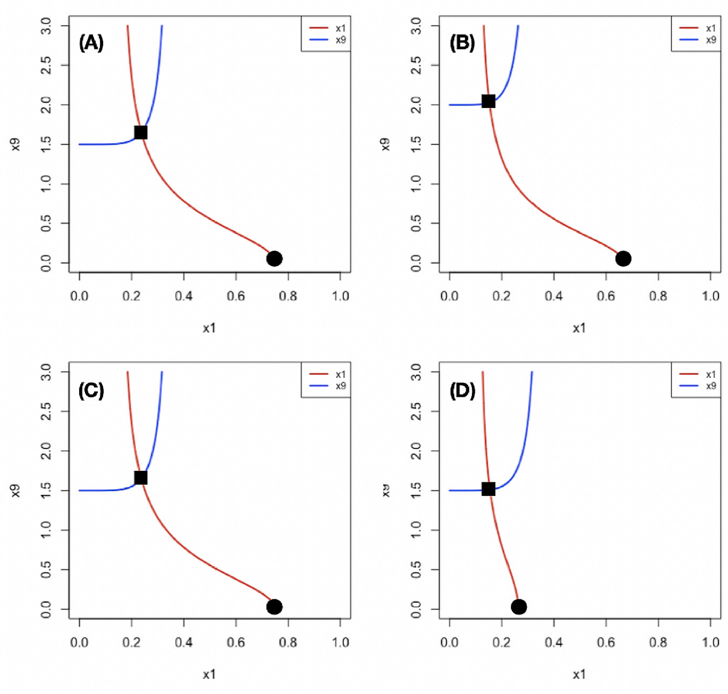

Fig 12. Positive feedback equilibrium states for the TP53(x_1)-P21(x_9) circuit. (A) 2D projection onto the plane $x_1 - x_9$ obtained by maintaining the other variables at a zero constant value. The red and blue lines represent the trajectories x_1 and x_9, respectively. The unstable state is represented by the square and the stable state is represented by the circle. (B) Equilibrium points for the variables x_1 and x_9 when the other gene actions take constant values that led to a normal phenotype. (C) Equilibrium states for a precancer phenotype and (D) Equilibrium states for a cancer phenotype. In the four panels the values of microenvironmental concentrations and thresholds were held at $y_i = 0.2$ and $\eta_i = 0.5$, with $i = 1, ...10$.

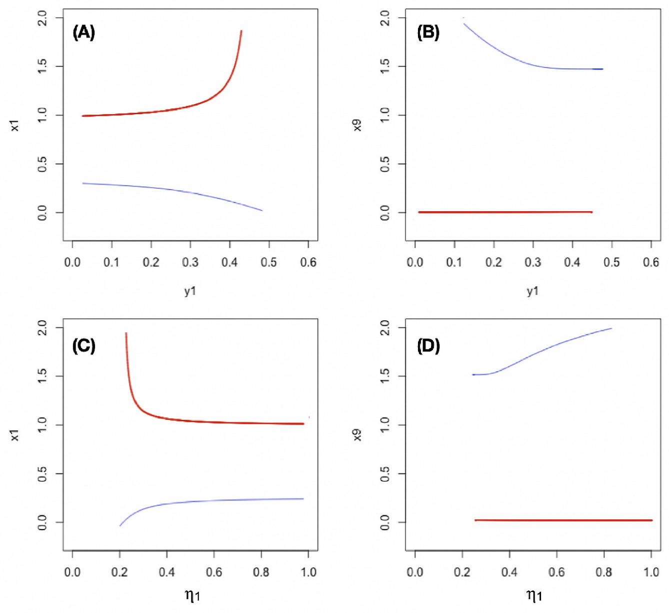

Fig 13. Multistability diagrams for the genetic states of the TP53(x_1)-P21(x_9) circuit for a normal phenotype. The blue lines indicate unstable states while the red lines represent stable states. (A) x_1 versus microenvironmental agent y_1. (B) x_9 versus microenvironmental agent y_1. (C) x_1 versus activation threshold η_1 and (D) x_9 versus activation threshold η_1.

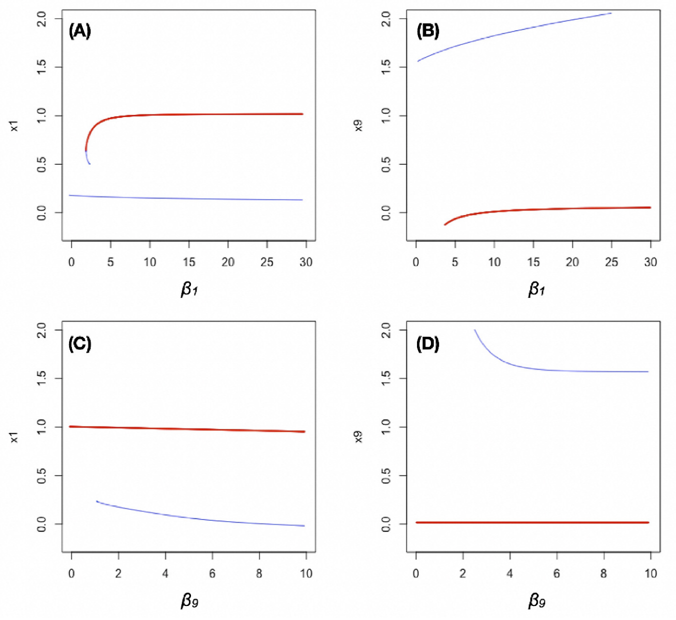

Fig 14. Multistability diagrams for the genetic states of the TP53(x_1)-P21(x_9) circuit for a normal phenotype. The blue lines indicate unstable states and the red lines represent stable states. (A) x_1 versus β_1. (B) G x_9 versus β_1. (C) x_1 versus β_9 and (D) x_9 versus β_9.

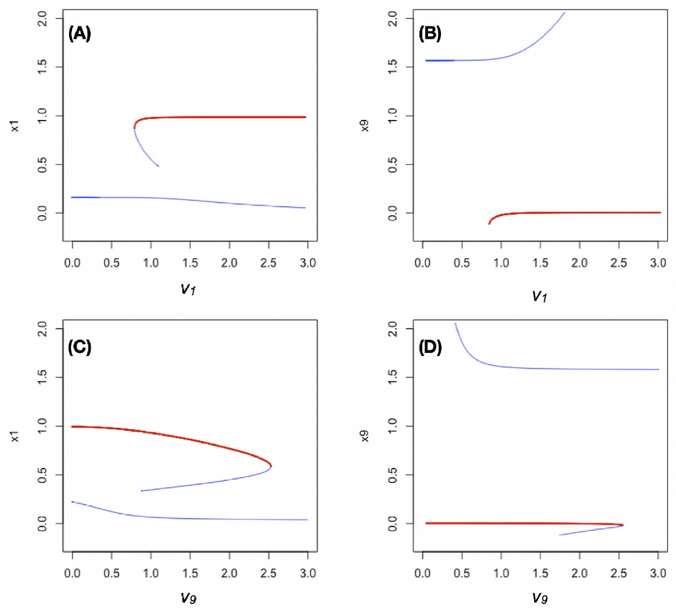

Fig 15. Multistability diagrams for the genetic states of the TP53(x_1)-P21(x_9) circuit for a normal phenotype. The blue lines indicate unstable states and the red lines represent stable states. (A) x_1 versus ν_1. (B) x_9 versus ν_1. (C) x_1 versus ν_9 and (D) x_9 versus ν_9.

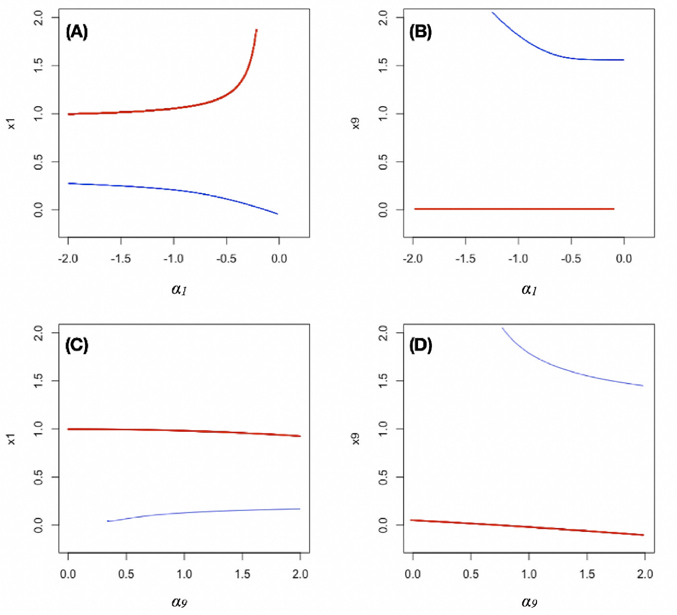

Fig 16. Multistability diagrams of the TP53(x_1)-P21(x_9) circuit for a normal phenotype. (A) Genetic state x_1 versus the interaction rate α_1. (B) Genetic state x_9 versus α_1. (C) x_1 versus α_9 and (D) x_9 versus α_9.

phenotypes. In addition, this analysis is in complete agreement with previous findings on the role of estrogen [19–22, 30] and give support to the model and results presented in this paper.

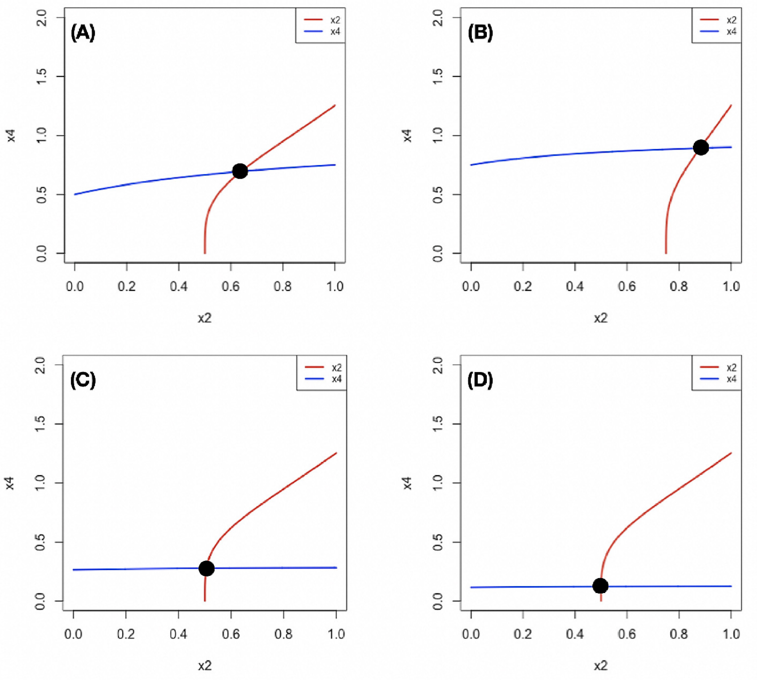

Fig 17. Positive feedback equilibrium states for ATR(x_2)-BRCA1(x_4) circuit. (A) 2D projection onto the plane $x_2 - x_4$ obtained by maintaining the other variables x_i at a zero constant value. The red and blue lines represent the x_2 and x_4 trajectories, respectively. The only stable state is represented by the circle. (B) Equilibrium points for the variables $x_2 - x_4$ when the other gene actions take nonzero constant values and led to a normal phenotype. (C) Equilibrium states of a precancer phenotype and (D) Equilibrium states of a cancer phenotype. In the four panels the values of microenvironmental concentrations and thresholds were held at $y_i = 0.2$ and $\eta_i = 0.5$, with $i = 1, ...10$.

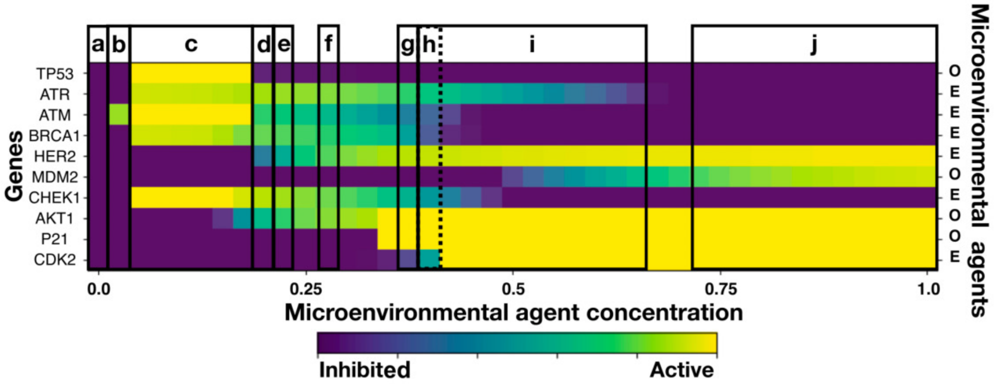

Fig 18. Microarrays showing gene expression and cell phenotypes. Normal phenotypes are represented by the states **a** and **c**. The states **b, d, e, f, g, h,** and **i** are identified with attractors in which some genes are Over-Expressed or Knocked-Out, suggesting a precancer phenotype. The state **j** is identified with the attractor that represents cancer phenotypes. The dotted line indicates an overlapping between states **h** and **i**. The initial threshold parameters for the set of ten genes were:
$\eta_{oi} = (0.3,\ 0.45,\ 0.15,\ 0.45,\ 0.15,\ 0.45,\ 0.3,\ 0.15,\ 0.3,\ 0.5)$.

Table 4. Wild-Type. Attractors with one state.

	Attr. 1	Attr. 2
**TP53**	0	0
**ATR**	0	1
**ATM**	0	0
**BRCA1**	0	1
**HER2**	0	0
**MDM2**	0	0
**CHEK1**	0	1
**AKT1**	0	0
**P21**	0	0
**CDK2**	0	0
Freq.	32.81%	1.56%

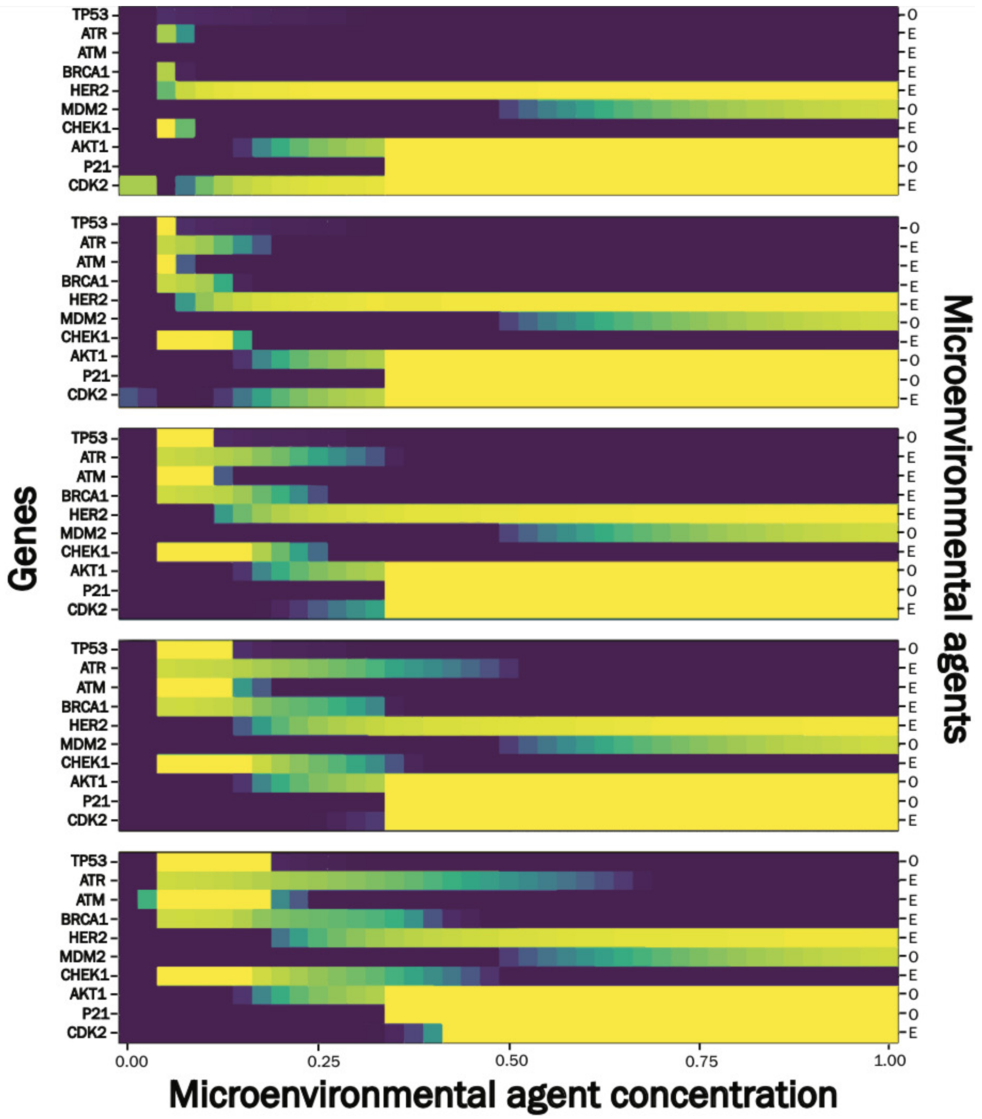

Fig 19. Microarrays showing the effects of increasing estrogen threshold values in the expression of all ten GRN genes. From top to bottom, the increase was as follows: 10%, 25%, 50%, 75% and 100%, in comparison with the values assigned to obtain the microarrays shown in Fig 18. The parameters and color bars are the same as in Fig 18

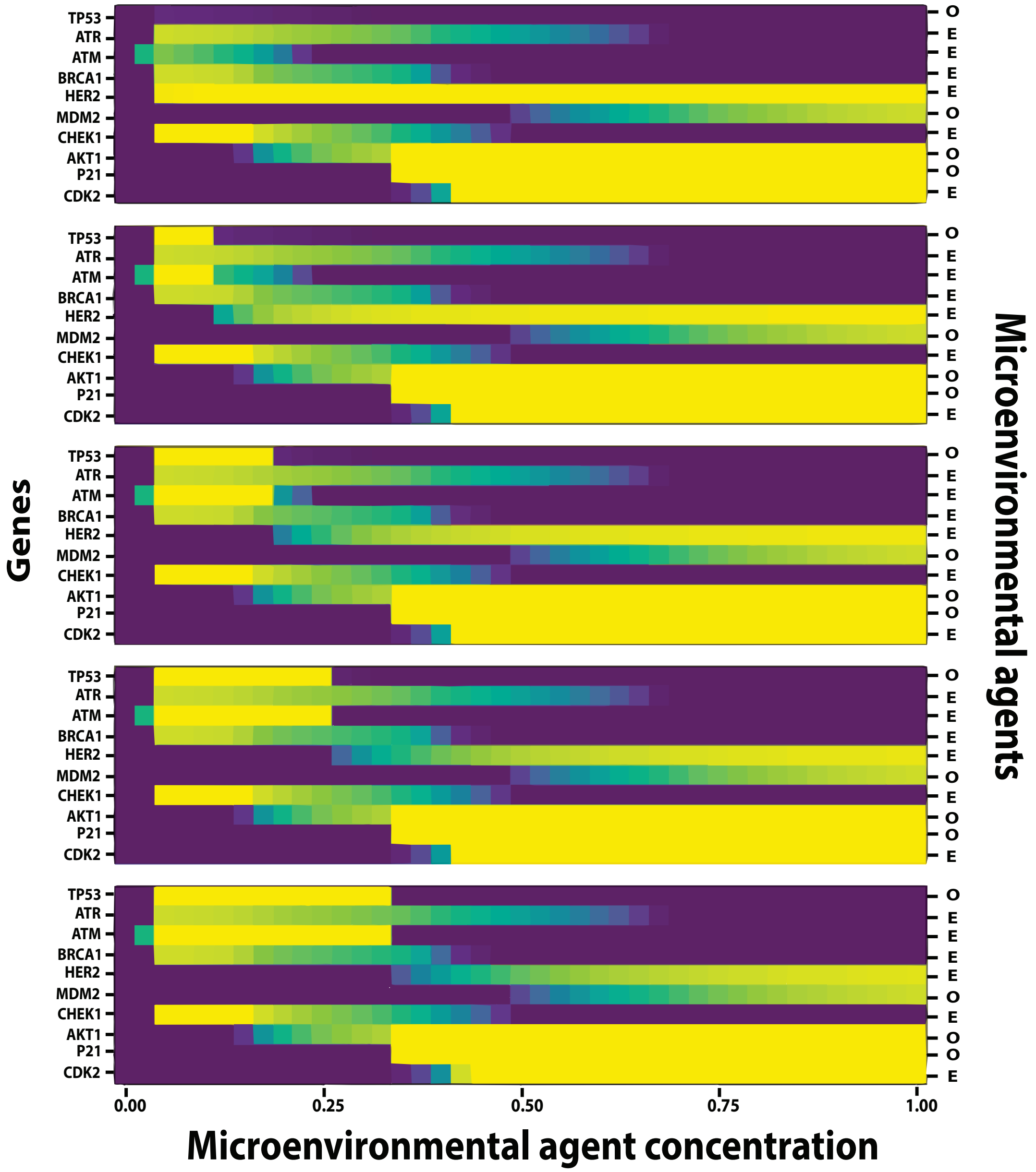

Fig 20. Microarrays showing the effects of increasing estrogen threshold value in gene HER2. From top to bottom, the increase was as follows: 0.1%, 50%, 100%, 150% and 200% as compared to the values assigned in Fig 18. The parameters and color bar are the same as in Fig 18

Table 5. Wild-Type. Attractors with two states.

	Attr. 3		Attr. 4	
**TP53**	0	0	0	0
**ATR**	1	0	0	0
**ATM**	0	0	0	0
**BRCA1**	0	1	0	0
**HER2**	0	0	1	1
**MDM2**	0	0	0	0
**CHEK1**	0	0	0	0
**AKT1**	0	0	1	1
**P21**	0	0	1	1
**CDK2**	0	0	0	1
Freq.	15.62%		50%	

Table 6. TP53 KO. Attractors with one state.

	Attr. 1	Attr. 2
**TP53**	0	0
**ATR**	0	1
**ATM**	0	0
**BRCA1**	0	1
**HER2**	0	0
**MDM2**	0	0
**CHEK1**	0	1
**AKT1**	0	0
**P21**	0	0
**CDK2**	0	0
Freq.	32.81%	1.56%

Table 7. TP53 KO. Attractors with two states.

	Attr. 3		Attr. 4	
**TP53**	0	0	0	0
**ATR**	1	0	0	0
**ATM**	0	0	0	0
**BRCA1**	0	1	0	0
**HER2**	0	0	1	1
**MDM2**	0	0	0	0
**CHEK1**	0	0	0	0
**AKT1**	0	0	1	1
**P21**	0	0	1	1
**CDK2**	0	0	0	1
Freq.	15.62%		50%	

Table 8. TP53 OE. Attractors with one state.

	Attr. 1	Attr. 2	Attr. 3	Attr. 4
**TP53**	1	1	1	1
**ATR**	0	1	0	1
**ATM**	1	1	1	1
**BRCA1**	0	1	0	1
**HER2**	0	0	1	1
**MDM2**	0	0	0	0
**CHEK1**	0	1	0	1
**AKT1**	0	0	0	0
**P21**	0	0	0	0
**CDK2**	0	0	0	0
Freq.	32.81%	1.56%	32.81%	1.56%

Table 9. TP53 OE. Attractors with two states.

	Attr. 5		Attr. 6	
**TP53**	1	1	1	1
**ATR**	1	0	1	0
**ATM**	1	1	1	1
**BRCA1**	0	1	0	1
**HER2**	0	0	1	1
**MDM2**	0	0	0	0
**CHEK1**	0	0	0	0
**AKT1**	0	0	0	0
**P21**	0	0	0	0
**CDK2**	0	0	0	0
Freq.	15.62%		15.62%	

Table 10. ATR KO. Attractors with one state.

	Attr. 1
**TP53**	0
**ATR**	0
**ATM**	0
**BRCA1**	0
**HER2**	0
**MDM2**	0
**CHEK1**	0
**AKT1**	0
**P21**	0
**CDK2**	0
Freq.	50%

Table 11. ATR KO. Attractors with two states.

	Attr. 2	
**TP53**	0	0
**ATR**	0	0
**ATM**	0	0
**BRCA1**	0	0
**HER2**	1	1
**MDM2**	0	0
**CHEK1**	0	0
**AKT1**	1	1
**P21**	1	1
**CDK2**	0	1
Freq.	50%	

Table 12. ATR OE. Attractors with one state.

	Attr. 1
**TP53**	0
**ATR**	1
**ATM**	0
**BRCA1**	1
**HER2**	0
**MDM2**	0
**CHEK1**	1
**AKT1**	0
**P21**	0
**CDK2**	0
Freq.	50%

Table 13. ATR OE. Attractors with two states.

	Attr. 2	
**TP53**	0	0
**ATR**	1	1
**ATM**	0	0
**BRCA1**	0	0
**HER2**	1	1
**MDM2**	0	0
**CHEK1**	0	0
**AKT1**	1	1
**P21**	1	1
**CDK2**	0	1
Freq.	50%	

Table 14. ATM KO. Attractors with one state.

	Attr. 1	Attr. 2
**TP53**	0	0
**ATR**	0	1
**ATM**	0	0
**BRCA1**	0	1
**HER2**	0	0
**MDM2**	0	0
**CHEK1**	0	1
**AKT1**	0	0
**P21**	0	0
**CDK2**	0	0
Freq.	32.81%	1.56%

Table 15. ATM KO. Attractors with two states.

	Attr. 3		Attr. 4	
**TP53**	0	0	0	0
**ATR**	1	0	0	0
**ATM**	0	0	0	0
**BRCA1**	0	1	0	0
**HER2**	0	0	1	1
**MDM2**	0	0	0	0
**CHEK1**	0	0	0	0
**AKT1**	0	0	1	1
**P21**	0	0	1	1
**CDK2**	0	0	0	1
Freq.	15.62%		50%	

Table 16. ATM OE. Attractors with one state.

	Attr. 1	Attr. 2
**TP53**	0	0
**ATR**	0	1
**ATM**	1	1
**BRCA1**	0	1
**HER2**	0	0
**MDM2**	0	0
**CHEK1**	0	1
**AKT1**	0	0
**P21**	0	0
**CDK2**	0	0
Freq.	32.81%	1.56%

Table 17. ATM OE. Attractors with two states.

	Attr. 3		Attr. 4	
**TP53**	0	0	0	0
**ATR**	1	0	0	0
**ATM**	1	1	1	1
**BRCA1**	0	1	0	0
**HER2**	0	0	1	1
**MDM2**	0	0	0	0
**CHEK1**	0	0	0	0
**AKT1**	0	0	1	1
**P21**	0	0	1	1
**CDK2**	0	0	0	1
Freq.	15.62%		50%	

Table 18. BRCA1 KO. Attractors with one state.

	Attr. 1
**TP53**	0
**ATR**	0
**ATM**	0
**BRCA1**	0
**HER2**	0
**MDM2**	0
**CHEK1**	0
**AKT1**	0
**P21**	0
**CDK2**	0
Freq.	50%

Table 19. BRCA1 KO.Attractors with two states.

	Attr. 2	
**TP53**	0	0
**ATR**	0	0
**ATM**	0	0
**BRCA1**	0	0
**HER2**	1	1
**MDM2**	0	0
**CHEK1**	0	0
**AKT1**	1	1
**P21**	1	1
**CDK2**	0	1
Freq.	50%	

Table 20. BRCA1 OE. Attractors with one state.

	Attr. 1
**TP53**	0
**ATR**	1
**ATM**	0
**BRCA1**	1
**HER2**	0
**MDM2**	0
**CHEK1**	1
**AKT1**	0
**P21**	0
**CDK2**	0
Freq.	50%

Table 21. BRCA1 OE. Attractors with two states.

	Attr. 2	
**TP53**	0	0
**ATR**	1	1
**ATM**	0	0
**BRCA1**	1	1
**HER2**	1	1
**MDM2**	0	0
**CHEK1**	0	0
**AKT1**	1	1
**P21**	1	1
**CDK2**	0	1
Freq.	50%	

Table 22. HER2 KO. Attractors with one state.

	Attr. 1	Attr. 2
**TP53**	0	0
**ATR**	0	1
**ATM**	0	0
**BRCA1**	0	1
**HER2**	0	0
**MDM2**	0	0
**CHEK1**	0	1
**AKT1**	0	0
**P21**	0	0
**CDK2**	0	0
Freq.	65.62%	3.12%

Table 23. HER2 KO. Attractors with two states.

	Attr. 3	
**TP53**	0	0
**ATR**	1	0
**ATM**	0	0
**BRCA1**	0	1
**HER2**	0	0
**MDM2**	0	0
**CHEK1**	0	0
**AKT1**	0	0
**P21**	0	0
**CDK2**	0	0
Freq.	31.25%	

Table 24. HER2 OE. Attractors with two states.

	Attr. 1	
**TP53**	0	0
**ATR**	0	0
**ATM**	0	0
**BRCA1**	0	0
**HER2**	1	1
**MDM2**	0	0
**CHEK1**	0	0
**AKT1**	1	1
**P21**	1	1
**CDK2**	0	1
Freq.	100%	

Table 25. MDM2 KO. Attractors with one state.

	Attr. 1	Attr. 2
**TP53**	0	0
**ATR**	0	1
**ATM**	0	0
**BRCA1**	0	1
**HER2**	0	0
**MDM2**	0	0
**CHEK1**	0	1
**AKT1**	0	0
**P21**	0	0
**CDK2**	0	0
Freq.	32.81%	1.56%

Table 26. MDM2 KO. Attractors with two states.

	Attr. 3		Attr. 4	
**TP53**	0	0	0	0
**ATR**	1	0	0	0
**ATM**	0	0	0	0
**BRCA1**	0	1	0	0
**HER2**	0	0	1	1
**MDM2**	0	0	0	0
**CHEK1**	0	0	0	0
**AKT1**	0	0	1	1
**P21**	0	0	1	1
**CDK2**	0	0	0	1
Freq.	15.62%		50%	

Table 27. MDM2 OE. Attractors with one state.

	Attr. 1	Attr. 2
**TP53**	0	0
**ATR**	0	1
**ATM**	0	0
**BRCA1**	0	1
**HER2**	0	0
**MDM2**	1	1
**CHEK1**	0	1
**AKT1**	0	0
**P21**	0	0
**CDK2**	0	0
Freq.	32.81%	1.56%

Table 28. MDM2 OE. Attractors with two states.

	Attr. 3		Attr. 4	
**TP53**	0	0	0	0
**ATR**	1	0	0	0
**ATM**	0	0	0	0
**BRCA1**	0	1	0	0
**HER2**	0	0	1	1
**MDM2**	1	1	1	1
**CHEK1**	0	0	0	0
**AKT1**	0	0	1	1
**P21**	0	0	1	1
**CDK2**	0	0	0	1
Freq.	15.62%		50%	

Table 29. CHEK1 KO. Attractors with one state.

	Attr. 1	Attr. 2
**TP53**	0	0
**ATR**	0	1
**ATM**	0	0
**BRCA1**	0	1
**HER2**	0	0
**MDM2**	0	0
**CHEK1**	0	0
**AKT1**	0	0
**P21**	0	0
**CDK2**	0	0
Freq.	32.81%	1.56%

Table 30. CHEK1 KO. Attractors with two states.

	Attr. 3		Attr. 4	
**TP53**	0	0	0	0
**ATR**	1	0	0	0
**ATM**	0	0	0	0
**BRCA1**	0	1	0	0
**HER2**	0	0	1	1
**MDM2**	0	0	0	0
**CHEK1**	0	0	0	0
**AKT1**	0	0	1	1
**P21**	0	0	1	1
**CDK2**	0	0	0	1
Freq.	15.62%		50%	

Table 31. CHEK1 OE. Attractors with one state.

	Attr. 1	Attr. 2
**TP53**	0	0
**ATR**	0	1
**ATM**	0	0
**BRCA1**	0	1
**HER2**	0	0
**MDM2**	0	0
**CHEK1**	1	1
**AKT1**	0	0
**P21**	0	0
**CDK2**	0	0
Freq.	32.81%	1.56%

Table 32. CHEK1 OE. Attractors with two states.

	Attr. 3		Attr. 4	
**TP53**	0	0	0	0
**ATR**	1	0	0	0
**ATM**	0	0	0	0
**BRCA1**	0	1	0	0
**HER2**	0	0	1	1
**MDM2**	0	0	0	0
**CHEK1**	1	1	1	1
**AKT1**	0	0	1	1
**P21**	0	0	1	1
**CDK2**	0	0	0	1
Freq.	15.62%		50%	

Table 33. AKT1 KO. Attractors with one state.

	Attr. 1	Attr. 2
**TP53**	0	0
**ATR**	0	1
**ATM**	0	0
**BRCA1**	0	1
**HER2**	0	0
**MDM2**	0	0
**CHEK1**	0	1
**AKT1**	0	0
**P21**	0	0
**CDK2**	0	0
Freq.	21.88%	3.12%

Table 34. AKT1 KO. Attractors with two states.

	Attr. 3		Attr. 4		Attr. 5	
**TP53**	0	0	0	0	0	0
**ATR**	1	0	0	0	1	0
**ATM**	0	0	0	0	0	0
**BRCA1**	0	1	0	0	0	1
**HER2**	0	0	1	1	1	1
**MDM2**	0	0	0	0	0	0
**CHEK1**	0	0	0	0	0	0
**AKT1**	0	0	0	0	0	0
**P21**	0	0	1	1	1	1
**CDK2**	0	0	0	1	0	1
Freq.	25%		31.25%		18.75%	

Table 35. AKT1 OE. Attractors with one state.

	Attr. 1
**TP53**	0
**ATR**	0
**ATM**	0
**BRCA1**	0
**HER2**	0
**MDM2**	0
**CHEK1**	0
**AKT1**	1
**P21**	0
**CDK2**	0
Freq.	50%

Table 36. AKT1 OE. Attractors with two states.

	Attr. 2	
**TP53**	0	0
**ATR**	0	0
**ATM**	0	0
**BRCA1**	0	0
**HER2**	1	1
**MDM2**	0	0
**CHEK1**	0	0
**AKT1**	1	1
**P21**	1	1
**CDK2**	0	1
Freq.	50%	

Table 37. P21 KO. Attractors with one state.

	Attr. 1	Attr. 2	Attr. 3	Attr. 4
**TP53**	0	0	0	0
**ATR**	0	1	0	1
**ATM**	0	0	0	0
**BRCA1**	0	1	0	1
**HER2**	0	0	1	1
**MDM2**	0	0	0	0
**CHEK1**	0	1	0	1
**AKT1**	0	0	0	0
**P21**	0	0	0	0
**CDK2**	0	0	0	0
Freq.	21.88%	3.12%	21.88%	3.12%

Table 38. P21 KO. Attractors with two states.

	Attr. 5		Attr. 6	
**TP53**	0	0	0	0
**ATR**	1	0	1	0
**ATM**	0	0	0	0
**BRCA1**	0	1	0	1
**HER2**	0	0	1	1
**MDM2**	0	0	0	0
**CHEK1**	0	0	0	0
**AKT1**	0	0	0	0
**P21**	0	0	0	0
**CDK2**	0	0	0	0
Freq.	25%		25%	

Table 39. P21 OE. Attractors with two states.

	Attr. 1		Attr. 2	
**TP53**	0	0	0	0
**ATR**	0	0	0	0
**ATM**	0	0	0	0
**BRCA1**	0	0	0	0
**HER2**	0	0	1	1
**MDM2**	0	0	0	0
**CHEK1**	0	0	0	0
**AKT1**	1	1	1	1
**P21**	1	1	1	1
**CDK2**	0	1	0	1
Freq.	50%		50%	

Table 40. CDK2 KO. Attractors with one state.

	Attr. 1	Attr. 2	Attr. 3
**TP53**	0	0	0
**ATR**	0	1	0
**ATM**	0	0	0
**BRCA1**	0	1	0
**HER2**	0	0	1
**MDM2**	0	0	0
**CHEK1**	0	1	0
**AKT1**	0	0	1
**P21**	0	0	1
**CDK2**	0	0	0
Freq.	28.12%	3.12%	50%

Table 41. CDK2 KO. Attractors with two states.

	Attr. 4	
**TP53**	0	0
**ATR**	1	0
**ATM**	0	0
**BRCA1**	0	1
**HER2**	0	0
**MDM2**	0	0
**CHEK1**	0	0
**AKT1**	0	0
**P21**	0	0
**CDK2**	0	0
Freq.	18.75%	

Table 42. CDK2 OE. Attractors with one state.

	Attr. 1	Attr. 2
**TP53**	0	0
**ATR**	0	0
**ATM**	0	0
**BRCA1**	0	0
**HER2**	0	1
**MDM2**	0	0
**CHEK1**	0	0
**AKT1**	0	1
**P21**	0	1
**CDK2**	1	1
Freq.	50%	50%

References

1. Schwab M. Encyclopedia of cancer. Springer Science & Business Media; 2008.

2. Yu C, Wang J. A Physical Mechanism and Global Quantification of Breast Cancer. PLOS ONE. 2016;11(7):1–20. doi:10.1371/journal.pone.0157422.

3. Barrera M, Hiriart M, Cocho G, Villarreal C. Type 2 diabetes progression: A regulatory network approach. Chaos. 2020;30(9):093132. doi:10.1063/5.0011125.

4. Hernández-Hernández V, Barrio RA, Benítez M, Nakayama N, Romero-Arias JR, Villarreal C. A physico-genetic module for the polarisation of auxin efflux carriers PIN-FORMED (PIN). Physical Biology. 2018;15(3):036002.

5. Barrio RÁ, Hernández-Machado A, Varea C, Romero-Arias J, Alvarez-Buylla E. Flower development as an interplay between dynamical physical fields and genetic networks. PLoS One. 2010;5(10):e13523. doi:10.1371/journal.pone.0013523.

6. Garraway LA, Sellers WR. Lineage dependency and lineage-survival oncogenes in human cancer. Nature Reviews Cancer. 2006;6(8):593–602. doi:10.1038/nrc1947.

7. Angeli D, Ferrell JE, Sontag ED. Detection of multistability, bifurcations, and hysteresis in a large class of biological positive-feedback systems. Proceedings of the National Academy of Sciences. 2004;101(7):1822–1827.

8. Zhao Y, Aguilar A, Bernard D, Wang S. Small-molecule inhibitors of the MDM2-p53 protein-protein interaction (MDM2 Inhibitors) in clinical trials for cancer treatment. Journal of medicinal chemistry. 2015;58(3):1038–1052. doi:10.1021/jm501092z.

9. Adler M, Mayo A, Zhou X, Franklin RA, Jacox JB, Medzhitov R, et al. Endocytosis as a stabilizing mechanism for tissue homeostasis. Proceedings of the National Academy of Sciences. 2018;115(8):E1926–E1935. doi:10.1073/pnas.1714377115.

10. RA W. The retinoblastoma protein and cell cycle control. Cell. 1995;81(3).

11. Darzacq X, Shav-Tal Y, de Turris V, Brody Y, Shenoy SM, Phair RD, et al. In vivo dynamics of RNA polymerase II transcription. Nature Structural & Molecular Biology. 2007;14(9):796–806. doi:10.1038/nsmb1280.

12. Larance M, Ahmad Y, Kirkwood KJ, Ly T, Lamond AI. Global subcellular characterization of protein degradation using quantitative proteomics. Molecular & cellular proteomics : MCP. 2013;12(3):638–650. doi:10.1074/mcp.M112.024547.

13. Eden E, Geva-Zatorsky N, Issaeva I, Cohen A, Dekel E, Danon T, et al. Proteome Half-Life Dynamics in Living Human Cells. Science. 2011;331(6018):764–768. doi:10.1126/science.1199784.

14. Chène P. Inhibiting the p53–MDM2 interaction: an important target for cancer therapy. Nature Reviews Cancer. 2003;3(2):102–109. doi:10.1038/nrc991.

15. Nag S, Qin J, Srivenugopal KS, Wang M, Zhang R. The MDM2-p53 pathway revisited. Journal of biomedical research. 2013;27(4):254–271. doi:10.7555/JBR.27.20130030.

16. Oliner JD, Saiki AY, Caenepeel S. The Role of MDM2 Amplification and Overexpression in Tumorigenesis. Cold Spring Harbor perspectives in medicine. 2016;6(6):a026336. doi:10.1101/cshperspect.a026336.

17. Hou H, Sun D, Zhang X. The role of MDM2 amplification and overexpression in therapeutic resistance of malignant tumors. Cancer cell international. 2019;19:216–216. doi:10.1186/s12935-019-0937-4.

18. Kung CP, Weber JD. It's Getting Complicated-A Fresh Look at p53-MDM2-ARF Triangle in Tumorigenesis and Cancer Therapy. Frontiers in cell and developmental biology. 2022;10:818744–818744. doi:10.3389/fcell.2022.818744.

19. Russo J, Russo IH. The role of estrogen in the initiation of breast cancer. The Journal of Steroid Biochemistry and Molecular Biology. 2006;102(1):89–96. doi:https://doi.org/10.1016/j.jsbmb.2006.09.004.

20. Huang B, Warner M, Gustafsson JÅ. Estrogen receptors in breast carcinogenesis and endocrine therapy. Molecular and Cellular Endocrinology. 2015;418:240–244. doi:https://doi.org/10.1016/j.mce.2014.11.015.

21. Sun Y. Tumor microenvironment and cancer therapy resistance. Cancer Letters. 2016;380(1):205–215. doi:https://doi.org/10.1016/j.canlet.2015.07.044.

22. Gupta PB, Pastushenko I, Skibinski A, Blanpain C, Kuperwasser C. Phenotypic Plasticity: Driver of Cancer Initiation, Progression, and Therapy Resistance. Cell Stem Cell. 2019;24(1):65–78. doi:10.1016/j.stem.2018.11.011.

23. Gérard C, Goldbeter A. From quiescence to proliferation: Cdk oscillations drive the mammalian cell cycle. Frontiers in Physiology. 2012;3. doi:10.3389/fphys.2012.00413.

24. Giono LE, Manfredi JJ. Mdm2 is required for inhibition of Cdk2 activity by p21, thereby contributing to p53-dependent cell cycle arrest. Molecular and cellular biology. 2007;27(11):4166–4178. doi:10.1128/MCB.01967-06.

25. Schwanhäusser B, Busse D, Li N, Dittmar G, Schuchhardt J, Wolf J, et al. Global quantification of mammalian gene expression control. Nature. 2011;473(7347):337–342. doi:10.1038/nature10098.

26. Zhou X, Franklin RA, Adler M, Jacox JB, Bailis W, Shyer JA, et al. Circuit Design Features of a Stable Two-Cell System. Cell. 2018;172(4):744–757. doi:10.1016/j.cell.2018.01.015.

27. Briat C, Gupta A, Khammash M. Antithetic Integral Feedback Ensures Robust Perfect Adaptation in Noisy Biomolecular Networks. Cell Syst. 2016;2(1):15–26. doi:10.1016/j.cels.2016.01.004.

28. Shah NA, Sarkar CA. Robust network topologies for generating switch-like cellular responses. PLoS Comput Biol. 2011;7(6):e1002085. doi:10.1371/journal.pcbi.1002085.

29. Burstein HJ. The distinctive nature of HER2-positive breast cancers. The New England Journal of Medicine. 2005;353(16):1652 –1554.

30. Mobley JA, Brueggemeier RW. Estrogen receptor-mediated regulation of oxidative stress and DNA damage in breast cancer. Carcinogenesis. 2004;25(1):3–9. doi:10.1093/carcin/bgg175.
